# Expert consensus statement on venovenous extracorporeal membrane oxygenation ECMO for COVID-19 severe ARDS: an international Delphi study

**DOI:** 10.1186/s13613-023-01126-9

**Published:** 2023-05-02

**Authors:** Ahmed A. Rabie, Alyaa Elhazmi, Mohamed H. Azzam, Akram Abdelbary, Ahmed Labib, Alain Combes, Bishoy Zakhary, Graeme MacLaren, Ryan P. Barbaro, Giles J. Peek, Marta Velia Antonini, Kiran Shekar, Abdulrahman Al‐Fares, Pranay Oza, Yatin Mehta, Huda Alfoudri, Kollengode Ramanathan, Mark Ogino, Lakshmi Raman, Matthew Paden, Daniel Brodie, Robert Bartlett

**Affiliations:** 1 Critical Care Department-ECMO care Unit (ECU), Riyadh Region Cluster1, King Saud Medical City, Riyadh, Saudi Arabia; 2 Internal Medicine Department, King Faisal University, Riyadh, Saudi Arabia; 3 Adult Critical Care Department, Dr. Sulaiman Alhabib Medical Group, Jeddah, Saudi Arabia; 4 Critical Care Department, Cairo University, Cairo, Egypt; 5 Hamad Medical Corporation, Weill Cornell Medical College, Doha, Qatar; 6 INSERM, UMRS_1166-ICAN, Institute of Cardiometabolism and Nutrition, Sorbonne Université, 75013 Paris, France; 7 Service de Médecine Intensive-Réanimation, Institut de Cardiologie, APHP Sorbonne Université Hôpital Pitié-Salpêtrière, 75013 Paris, France; 8 Oregon Health and Science University, Portland, OR USA; 9 Cardiothoracic ICU, National University Hospital, Singapore, Singapore; 10 Division of Pediatric Critical Care and Susan B. Meister Child Health Evaluation and Research Center, University of Michigan, Ann Arbor, MI USA; 11 Congenital Heart Center, University of Florida, Gainesville, FL USA; 12 General Intensive Care Unit, University Hospital of Parma, Parma, Italy; 13 Adult Intensive Care Services, The Prince Charles Hospital, Brisbane, QLD Australia; 14 Faculty of Medicine, Institute of Health and Biomedical Innovation, Queensland University of Technology, Brisbane, Australia; 15 Department of Anesthesia, Critical Care Medicine and Pain Medicine, Ministry of Health, Kuwait City, Kuwait; 16 Al‐Amiri Hospital Center for Respiratory and Cardiac Failure, Kuwait Extracorporeal Life Support Program, Ministry of Health, Kuwait City, Kuwait; 17 Riddhi Vinayak Multispecialty Hospital, Mumbai, India; 18 Medanta Institute of Critical Care and Anesthesiology, Medanta The Medicity, Sector-38, Gurgaon, 122001 Haryana India; 19 Department of Anaesthesia, Critical Care, and Pain Management, Al‐Adan Hospital Ministry of Health, Hadiya, Kuwait; 20 Chief Partnership Officer, Nemours Children’s Health, Delaware Valley, USA; 21 Division of Paediatric Critical Care, University of Texas, Southwestern Medical Center, Dallas, TX USA; 22 Division of Paediatric Critical Care, Emory University, Atlanta, GA USA; 23 Department of Medicine, Columbia University College of Physicians & Surgeons, and Center for Acute Respiratory Failure, New York-Presbyterian/Columbia University Medical Center, New York, USA; 24 University of Michigan, Ann Arbor, MI USA

**Keywords:** ECMO, COVID-19, Consensus, Delphi, Statement

## Abstract

**Background:**

The high-quality evidence on managing COVID-19 patients requiring extracorporeal membrane oxygenation (ECMO) support is insufficient. Furthermore, there is little consensus on allocating ECMO resources when scarce. The paucity of evidence and the need for guidance on controversial topics required an international expert consensus statement to understand the role of ECMO in COVID-19 better. Twenty-two international ECMO experts worldwide work together to interpret the most recent findings of the evolving published research, statement formulation, and voting to achieve consensus.

**Objectives:**

To guide the next generation of ECMO practitioners during future pandemics on tackling controversial topics pertaining to using ECMO for patients with COVID-19-related severe ARDS.

**Methods:**

The scientific committee was assembled of five chairpersons with more than 5 years of ECMO experience and a critical care background. Their roles were modifying and restructuring the panel’s questions and, assisting with statement formulation in addition to expert composition and literature review. Experts are identified based on their clinical experience with ECMO (minimum of 5 years) and previous academic activity on a global scale, with a focus on diversity in gender, geography, area of expertise, and level of seniority. We used the modified Delphi technique rounds and the nominal group technique (NGT) through three face-to-face meetings and the voting on the statement was conducted anonymously. The entire process was planned to be carried out in five phases: identifying the gap of knowledge, validation, statement formulation, voting, and drafting, respectively.

**Results:**

In phase I, the scientific committee obtained 52 questions on controversial topics in ECMO for COVID-19, further reviewed for duplication and redundancy in phase II, resulting in nine domains with 32 questions with a validation rate exceeding 75% (Fig. 1). In phase III, 25 questions were used to formulate 14 statements, and six questions achieved no consensus on the statements. In phase IV, two voting rounds resulted in 14 statements that reached a consensus are included in four domains which are: patient selection, ECMO clinical management, operational and logistics management, and ethics.

**Conclusion:**

Three years after the onset of COVID-19, our understanding of the role of ECMO has evolved. However, it is incomplete. Tota14 statements achieved consensus; included in four domains discussing patient selection, clinical ECMO management, operational and logistic ECMO management and ethics to guide next-generation ECMO providers during future pandemic situations.

**Supplementary Information:**

The online version contains supplementary material available at 10.1186/s13613-023-01126-9.

## Introduction

As the coronavirus disease 2019 (COVID‐19) pandemic persists, increasing data help guide the management of COVID-19-related acute respiratory distress syndrome (ARDS). Unfortunately, there is minimal high-quality evidence or consensus on managing COVID-19 patients requiring extracorporeal membrane oxygenation (ECMO) support. Furthermore, there is little consensus on allocating ECMO resources when scarce. The paucity of evidence and the need for guidance spurred the scientific committee to create an international expert consensus statement on the topic. Five international ECMO experts who constituted the scientific committee. Their roles are the expert composition of the panel, interpretation of the most recent findings of the evolving published research, validation of questions on controversial topics, and statement formulation. The expert consensus aimed to provide ECMO practitioners worldwide with guidance on tackling controversial topics pertaining to the use of ECMO for patients with severe COVID-19-related ARDS during the next generation of future pandemics.

## Methods

The ECMO center in King Saud Medical City (KSMC) in Riyadh, Kingdom of Saudi Arabia (KSA), through the Saudi Arabia Ministry of Health (MOH) National ECMO program, is the coordinating center that supervised and coordinated the consensus process and conducted anonymous voting on the statements. This project was conducted in compliance with the Conducting and Reporting of Delphi Studies (CREDES) standards [1] and the Standards for Quality Improvement Reporting Excellence (SQUIRE) reporting guidelines [2]. A mix of modified Delphi technique rounds and nominal group technique (NGT) through three face-to-face meetings were used [3, 4]. The consensus principle followed cooperative behavior, and members were urged to adopt a stand-aside position whenever practicable. On December 15, 2021, the scientific committee was assembled of five chairpersons with more than 5 years of ECMO experience and a critical care background. The scientific committee’s role was to modify and restructure the panel's questions and formulate statements when necessary in addition to expert composition and literature review.

### Expert composition

Experts, including guest authors, are identified based on their clinical experience with ECMO (minimum of 5 years) and previous academic activity on a global scale, with a focus on diversity in gender, geography, area of expertise, and level of seniority; two experts, at least from each continent demonstrating divergent perspectives on particular conflicting topics addressed in their publications. The total number of invited experts is 32. The faculty includes 22 panelists representing both sexes, various countries, and ethnicities; half of the experts are not active members of ELSO; they represent different societies in addition to non-physician ECMO providers and one methodologist. The remaining experts will be brought in as “guest authors”, contributing to validating questions and creating statements but not voting on them in Delphi rounds. The entire process was scheduled to take place over 8 months, during which five phases were to be completed and planned as the following:

*Phase I* (December 15, 2021–January 15, 2022). The scientific committee works to identify gaps in knowledge, educate the faculty and ensure that they understand the consensus technique. The panel is then asked to identify areas of uncertainty.

*Phase II* (February 1, 2022–February 27, 2022). Two tasks are needed: domain creation and a survey for validation. During domain creation, the scientific committee review questions from the previous phase for appropriateness (lack of clear evidence or recommendation) and value (important aspects faced frequently). Duplications and redundancy are to be excluded. A web-based survey using google forms is conducted to validate the questions. A validation rate > 75% means three-quarters of the panel agrees about the appropriateness of the question to be addressed, and all demonstrate controversial topics in the literature.

*Phase III* (February 28, 2022–May 6, 2022). This phase includes four consecutive meetings, hybrid face-to-face, and virtual open discussions, on the survey results of validated questions, and the experts’ opinions are recorded. The main objective is to formulate the draft of the statements. During this phase, the ten international guest authors are invited to join in-person meetings to share their expertise and participate in statement formulation. Nevertheless, they do not vote on the formulated statements.

The first meeting is on February 28, 2022, in Riyadh The second meeting is on April 15, 2022, in Riyadh. The third meeting is on May 6, 2022, in London. The fourth meeting is conducted virtually on May 28, 2022, and all statements are to be finalized in preparation for voting.

*Phase IV* (June 25, 2022, and July 12, 2022). The coordinating KSMC ECMO center research team conducts two rounds of voting using a voting platform appropriate for Delphi exercises to implement the voting process independently and anonymously complete the required analysis. The results will then be sent to the expert panel after each round. The coordinating team communicates with the scientific committee to review the statements that do not reach the quorum in the first round for possible modification.

### Voting rules

The voting process was anonymous to avoid any reciprocal influence and dredging effects. The panel members voted on a 10-point scale ranging from totally disagree to totally agree. The responses were grouped and stratified into low (score 1–3), intermediate (score 4–7), and high (score 8–10) levels of agreement. Intermediate answers were defined as ‘I do not agree or disagree’ and not ‘I do not have an opinion’. In addition to providing the vote, each panel member was invited to comment and provide suggestions to modify statement content and improve the wording, following an iterative process. After the first round, those questions for which there was only weak or no consensus was resubmitted in a second round after being modified based on the panel comments and suggestions. Responses were analyzed with correspondence analysis (CA) and multiple correspondence analysis (MCA) to identify specific polarization patterns at the statement or voter level. If such patterns were found at the voter level, the coordinating team individually contacted the voter to rule out the misunderstanding and incorrect interpretations of the question, thus providing an opportunity to amend the question.

### Decision rule

The degree of consensus on the statements was established as follows: votes with a high score (> 80%) or a mean greater than eight were required to provide strong consensus, and votes with an intermediate score (70–80%) or mean 7–8 provided weak consensus, while a low score (less than 70%) or mean rating 6 provided no consensus, assuming all panel members understood the statement and after at least two rounds [1].

*Phase V* (July 15, 2021–August 25, 2022) was the final stage, where the writing committee assembled, wrote, and reviewed the final manuscript before approval by all faculty.

### Statistical analysis

Simple descriptive statistics (response rates, level of agreement for each statement, and mean levels of the scores) were used to describe approval rates between rounds. The same measures were used to evaluate consensus stability across rounds.

## Results

**Fig. 1:**
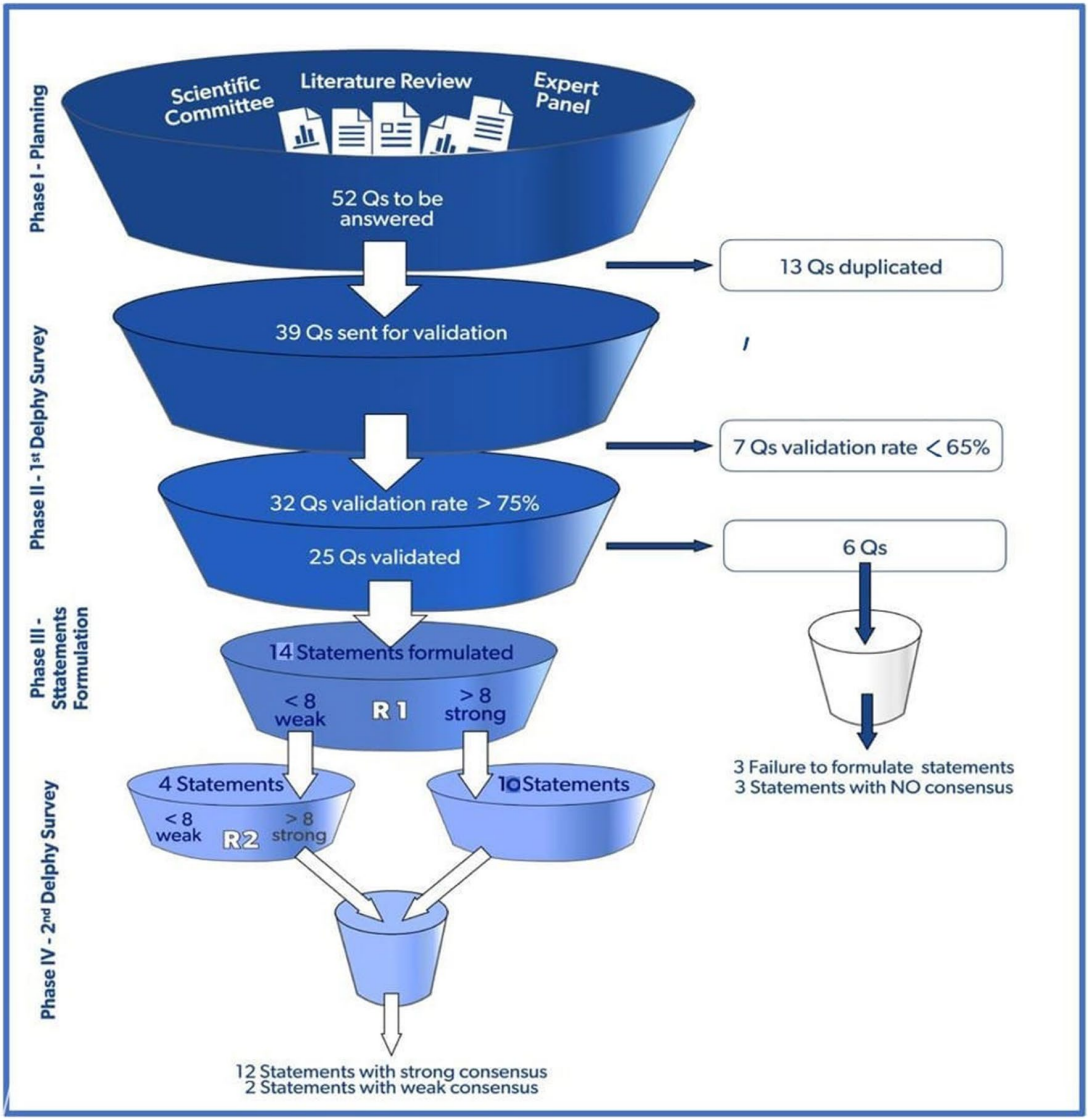
The flowchart depicted a timeline of questions and statements created during the consensus

In phase I, the scientific committee obtained 52 questions about controversial topics in ECMO for COVID-19 (Fig. 1) which was furtherly reviewed for duplication and redundancy in phase II, resulting in 32 questions with a validation rate exceeding 75% (Fig. 1). In phase III 25 questions were used to formulate 14 statements, and six questions achieved no consensus on the statements. In phase IV, the first voting round, R1, 10 statements reached a strong consensus. Four statements reached a weak consensus, modified according to the participants’ comments in R1 in preparation for voting in the second round. In the second voting round, R2, only two statements out of four reached a strong consensus. The final 14 statements that reached consensus after voting rounds are as the following (Table 1).

**Table 1:**
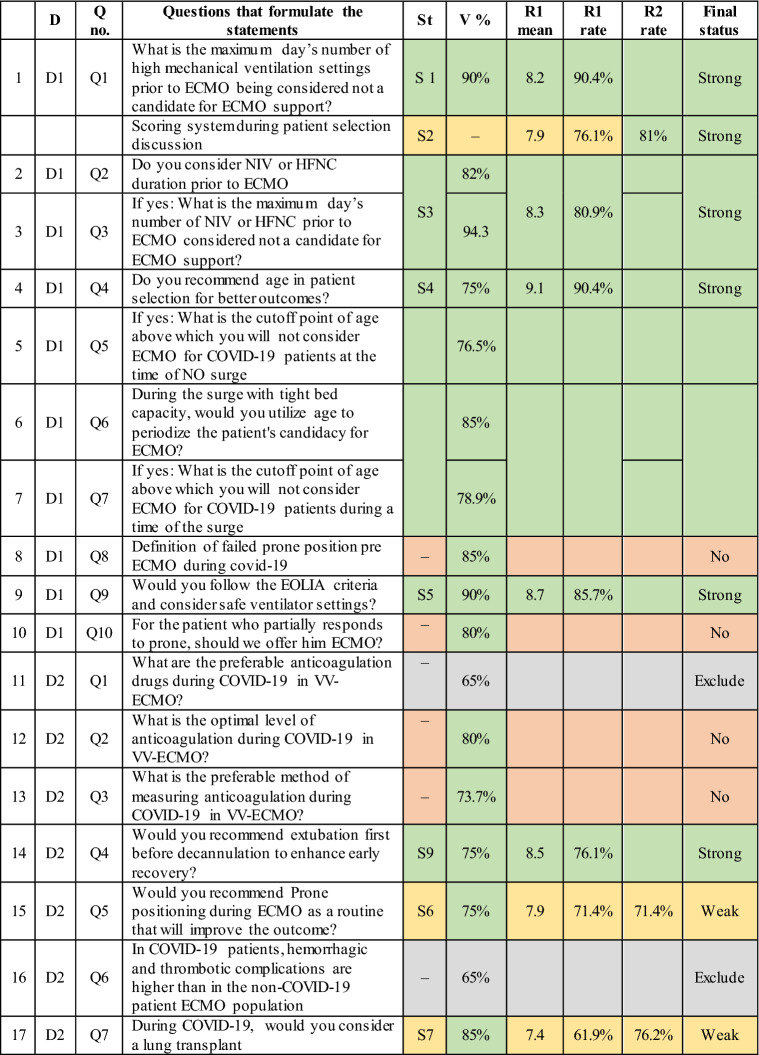

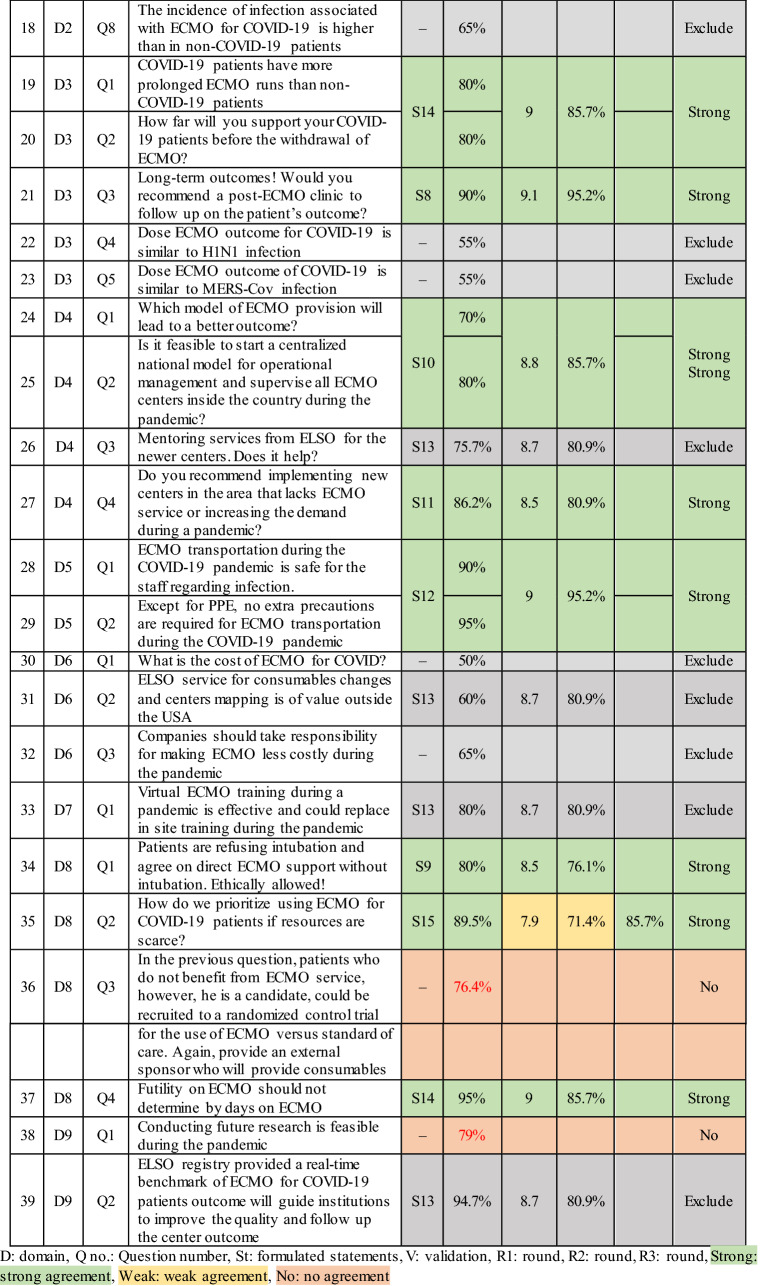
Questions included to formulate statements of the consensus showed validation rate and voting rounds result

### Domain 1: patient selection

***Statement 1.*** (Strong consensus, mean score 8.2, score rate 90.4%, round 1 (R1) had high score)

The duration of invasive mechanical ventilation (IMV) before considering ECMO should not be used as a primary determinant for ECMO candidacy. However, a longer IMV duration with high settings may help decision-making if concurrently considered with other factors, indicating a worse outcome.

*Rationale: The association between pre-ECMO ventilator days and patient outcomes is controversial. Prior to COVID-19, most research indicated a direct association between days of IMV prior to ECMO and outcome, considering it as a prognostic criterion incorporated into multiple outcome prediction scores, e.g., RESP score* [5, 6]. *During the COVID-19 pandemic, studies have shown a variable association between days of IMV prior to ECMO and patient outcomes *[7–9]*. Nevertheless, the evidence in both cases is based on observational data, which is subject to bias. Therefore, further prospective, high-quality research is needed.*

***Statement 2.*** (Strong consensus, mean score 7.9, R1. Score rate 76.1% and R2. 81% R1 had moderate and R2 high scores)

There is no validated evidence-based scoring system to predict the outcome for COVID-19 patients receiving ECMO. Therefore, available scoring systems previously used for non-COVID-19 patients should not be used for COVID-19 patients as a prognostic tool.

*Rationale: To date, limited data for the scoring system are available to predict survival for COVID-19 ARDS patients receiving ECMO. Investigators seeking to validate existing scoring systems on COVID-19 patients found poor discrimination* [10, 11]. *Only the RESP score in the COVID-19 population demonstrated reasonable discrimination and slightly better calibration; however, further adaptation of the RESP score to the COVID-19 population may be needed. It should not be used as a substitute for clinical judgment* [12].

***Statement 3.*** (Strong consensus, mean score 8.3, score rate 80.9%, R1 had high score)

Days on noninvasive mechanical ventilation (NIMV) or high-flow nasal cannula (HFNC) prior to endotracheal intubation should not be considered alone when selecting COVID-19 patients for ECMO unless it is used along with other patient conditions that are unfavorable to ECMO candidacy.

*Rationale: The panel agrees that the number of days on NIMV or HFNC may be related to patient outcome with ECMO, based on clinical experience and some evidence suggesting an association* [13]. *However, the quality of evidence is weak, and we suggest that future studies be conducted to answer this important question* [14].

***Statement 4.*** (Strong consensus, mean score 9.1, score rate 90.4%, R1 had high score)

During a surge in patient volume, the cutoff age could vary worldwide depending on ECMO capacity and resource availability. As a result, we advise setting the cutoff age in a national or regional policy, if needed, due to limited resources.

*Rationale:* Age is strongly associated with patient outcomes and should be considered during patient selection. *All cohorts of ECMO in COVID-19 that studied predictors of mortality retained age in their regression analysis model, and the median age or age grouping was approximately 45–55 years. It is crucial to distinguish between numerical age and biological age, which is determined by the patient’s baseline function state, in addition to the fact that rationing based on age may be deemed inadmissible discrimination in jurisdictions of certain places* [15–17].

***Statement 5.*** (Strong consensus, mean score 8.7, score rate 85.7%, R1 had high score)

The panel recommends compliance with ECMO to Rescue Lung Injury in Severe ARDS (EOLIA) trial EOLIA criteria as indications for ECMO [18], without requiring deviations from the regular practice prior to COVID-19.

*Rationale: The randomized controlled (RCT) EOLIA trial *[16]* with subsequent *post hoc* Bayesian analysis *[19]* provided solid evidence on ECMO utilization, although it was statistically negative. Since then, ECMO practitioners have incorporated the trial's inclusion criteria, especially those that depend on Po*_*2*_*:Fio*_*2*_* and its duration, into their ECMO candidacy protocols. The practice of utilizing ECMO as a salvage therapy only for cases in which gas exchange cannot be maintained regardless to the safety of the mechanical ventilator settings due to resource constraints is clinically suboptimal and may be related to increased ECMO duration, and mortality associated with COVID-19 in regions that utilize this practice*. *Other factors may also contribute to these patients’ worsened outcomes* [20].

### Domain 2: ECMO management

***Statement 6.*** (Weak consensus, mean score 7.9, R1. Score rate 71.4% and R2. 71.9% R1 and R2 had moderate scores)

Prone positioning may be considered during ECMO management in experienced centers if there are sufficient resources.

*Rationale: Prone positioning during ECMO may improve outcomes in non-COVID-19 patients, with only limited evidence and experience in COVID-19 *[21, 22]*. There is no clear consensus on the timing, patient selection, and duration of prone positioning. On the other hand, prone positioning was safe and feasible when conducted at centers experienced in both ECMO and prone positioning* [23–25].

***Statement 7.*** (Weak consensus, mean score 7.4, R1. Score rate 61.9% and R2. 76.2% R1 had low score and R2 moderate score)

Lung transplantation for late COVID-19-related respiratory failure is feasible. However, it should be considered for ECMO patients supported for prolonged durations (months) without evidence of potential lung recovery and who are otherwise appropriate candidates for lung transplantation.

*Rationale: For patients with a positive COVID-19 test in the bronchial secretions, It is not advisable to undertake transplantation early during ECMO, although preparations may begin prior to full consideration. Unexpected lung recovery for patients on long-run ECMO that lasted months has been reported on numerous occasions; thus, patient candidacy for lung transplant should be assessed carefully* [26].

***Statement 8.*** (Strong consensus, mean score 9.1, score rate 95.2%, R1 had high score)

We suggest post-ECMO follow-up as part of a clinic or study to be initiated to assess long-term outcomes.

*Rationale: It is essential to understand the long-term mental, physical, and social impacts of ICU and ECMO care on these patients. Despite the partial recovery of the lung function tests at 1 year, some populations’ physical and psychological function remains impaired. Poor mental and physical health may be more related to COVID-19 than ECMO, although this needs confirmation, and persisting long-term symptoms suggest that dedicated post-ECMO follow-up programs are needed *[27, 28]*. When patients are discharged from the intensive care unit (ICU), the main objective should be reintegrating them into their previous societal roles. Patient follow-up after ICU discharge is a part of finishing what started as a critical illness* [29, 30].

***Statement 9.*** (Strong consensus, mean score 8.5, score rate 76.1%, R1 had high score)

A weaning strategy from ECMO, including liberation from IMV and allowing spontaneous breathing during ECMO, is feasible.

*Rationale: Early application of awake VV-ECMO without IMV in COVID-19 patients with severe ARDS and evidence of Macklin-like radiological signs on chest CT may result in barotrauma events, low intubation, and mortality rates *[31]*. Allowing spontaneous breathing during ECMO support could be challenging and requires close observation with more specific care and meticulous attention to arterial blood gases and respiratory patterns to protect against patient self-inflicted lung injury (P-SILI) *[32]*. These ECMO management requirements are feasible in a nonintubated, spontaneously breathing patient, as we realized that spontaneous breathing is of high success rate during ECMO in the case of lung transplant and COPD but only 50% of severe ARDS *[33]*. However, they are difficult to implement during a surge.*

### Domain 3: Operational and logistics

***Statement 10.*** (Strong consensus, mean score 8.7, score rate 85.7%, R1 had high score)

It is feasible to start a new centralized ECMO service in preparation for a pandemic, nationwide or regionally, at least at the level of patient selection and allocation of resources.

*Rationale: Regional centralization and organization of ECMO services have been shown to optimize resource utilization and proper patient selection in the SWAAC ELSO region.* ECMO *provision in countries that utilized this model avoided inappropriate utilization of ECMO and facilitated adequate prioritization for patients who may benefit from the service, favorable outcomes were achieved, and resource allocation was optimized* [34–38].

***Statement 11.*** (Strong consensus, mean score 8.5, score rate 80.9%, R1 had high score)

Establishing a new ECMO program in an area lacking ECMO services is feasible with restricted precautions, sufficient training, coordination with expert clinicians, centers, or societies, and careful patient selection.

*Rationale: It is recommended to collaborate with centralized ECMO services that will provide the required support if possible. Otherwise, maintain a partnership with ELSO by joining the registry to improve quality, enable benchmarking, and compare outcomes across centers* [39, 40].

***Statement 12.*** (Strong consensus, mean score 9, score rate 95.2%, R1 had high score)

Transportation of patients with COVID-19 on ECMO does not pose an appreciable risk to a well-trained mobile ECMO team using appropriate personal protective equipment.

*Rationale: A hub-and-spoke model and/or regional centralization of services is advocated by ELSO, especially during times of surge* [41]. *This allows for better resource allocation and utilization but requires robust transport systems. Adequate training, appropriate infection prevention control measures, personal protective equipment (PPE), and ambulance disinfection are essential for patient and medical personnel safety. Other transport safety suggestions may include adequate ventilation of the transport platform, the use of dedicated routes for patients with COVID-19 during intrahospital patient transport, and an additional filter on the expiratory limb of the transport ventilator* [41, 42].

### Domain 4: Ethics

***Statement 13***. (Strong consensus, mean score 9, score rate 85.7%, R1 had high score)

ECMO futility should not be determined solely by the duration of the ECMO run. Therefore, we advise against withdrawing ECMO due to prolonged ECMO until recovery, transplantation, or irreversible multiorgan failure.

*Rationale: The moral complexities of maintaining a patient on ECMO for an extended period of time and the anecdotal data and small observational studies demonstrated recovery of native lung function after prolonged ECMO runs exceeding 60 days* [43, 44]. *Conversely, the pandemic situation has resource constraints. It is wise to preserve resources for patients with better outcomes. Accordingly, to weigh both concerns, a patient is considered futile during a pandemic if scarce resources and a combination of factors such as multiorgan failure with ECMO duration days unless the lung has recovered or transplantation was planned but never utilized ECMO duration alone to consider the futility* [44, 45].

***Statement 14.*** (Strong consensus, mean score 7.9, R1. score rate 71.4% and R2 85.7%, R1 had moderate score and R2. high score)

Currently, there is no evidence-based scoring system to guide ECMO prioritization during resource limitations. However, factors predicting poor outcomes may be utilized to prioritize patients for ECMO.

*Rationale: Due to a lack of resources, ethical dilemmas regarding patient prioritization for ECMO pose unique challenges. Patients may be prioritized for ECMO treatment based on factors that predict poor outcomes. Increasing age, multiple comorbidities, and cumulative organ failures are the most common predictors of mortality and may determine which patients receive priority for ECMO. Which of these factors are used first to be prioritized for patient selection is difficult to define and cannot be generalized to all regions or clinical conditions* [46] (Additional file 1).

## Discussion

The COVID-19 pandemic regional caseloads, resource limitations, treatment advancements, and vaccination influenced ECMO utilization, leading to heterogeneity in ECMO provision among countries. Consequently, the evidence lags beyond reality, and guidelines lose relevance or support over time [47]. This study was designed to provide practical clinical guidance in approaching the debatable and controversial topics of ECMO for COVID-19 patients based on the best available evidence, expert opinion, and their interpretation of the most recent findings of increasingly published research.

Many societies and scientific organizations have played a major role during the pandemic [48–50]. ELSO’s role during the pandemic was noteworthy; it guided ECMO providers globally by releasing the initial guidance document [51] and publishing early guidelines [52] followed by updated guidelines [38]. It provided a valued multidirectional platform and played an essential role during the COVID-19 pandemic for healthcare workers using ECMO in the following ways: first, real-time registry of COVID-19 cases; second, providing virtual training, especially when travel and in-person meetings were limited; third, mentoring new ECMO programs and providing consultation, supervision, and assistance; fourth, establishing communication networks for sharing supplies and consumables and enabling ECMO center coordinators to compare the performance of their centers to that of other centers regionally and internationally [53].

During the first 2009 H1N1 pandemic, the annual sharp increase in ECMO-supported patients lasted for a decade. Subsequently, the COVID-19 pandemic was accompanied by a rise in ECMO utilization by practitioners who provide the service [54]. Likewise, we should anticipate an increase in the use of ECMO following the COVID-19 pandemic, as was the case after the first pandemic. Following these two pandemics, the efficacy of ECMO in providing respiratory support to patients with severe ARDS and refractory hypoxia is indisputable [55]. However, challenges in patient selection and clinical and operational management existed with a lack of solid evidence to support one regimen regardless of the significantly higher number of research publications compared to the H1N1 pandemic [56]. As a result, this consensus of expert opinion revealed all areas of uncertainty regarding the role of ECMO, which may encourage researchers to conduct high-quality research in the coming years to fill this knowledge gap.

The faculty in this study successfully reached a strong consensus on the most important debatable topics; however, no consensus was achieved on some topics, such as anticoagulation, pregnancy, and immunocompromised cases, despite the high validation rate exceeding 75% (Table 1); this could be due to the uncertainty raised from the lack of research and the absence of data to support one regimen or drug over the other. The panel discussion ended with no recommendation and concluded that no deviation from the standard of care is needed as recommended by published guidelines on anticoagulation management to define the best anticoagulant drugs or follow-up tests to be utilized [38].

The cost of ECMO services is another highly validated subject that has yet to reach a consensus (Table 1). It is one of the primary factors limiting the expansion of ECMO, particularly in low- and middle-income countries. Previous attempts to decrease costs were made by reducing staffing or shifting to a nurse-based ECMO model rather than a perfusionist approach, first thought to be more expensive [57]. The market price of consumables should decrease as more console models and more variations of consumables become available; at the very least, regional applications of economically based consumables costs will be affordable [58]. Judicious distribution of ECMO supply worldwide was a concern since many patients died from hypoxia despite having high ventilatory settings in the lack of ECMO supply capacities [45].

The Delphi and NGT methods utilized in this work are structured, systematic procedures for creating consensus recommendations; each has its advantages; in the Delphi, the participants vote anonymously, whereas NGT is typically a face-to-face process [59]; and each has its limitations. The Delphi technique’s most delicate methodological issue is the consensus definition. The investigators must determine how participant agreement will be measured and, if the agreement rate is employed, what threshold would be utilized to reach a consensus. Both strategies offer the chance to use the knowledge of experts to help clinical decision-making in problematic situations [3]. They are commonly used in medical research, especially when it is difficult to provide high-quality evidence. However, in most studies, the methods used to create a consensus are poorly reported and lack clarity, such as failing to define consensus or describing how consensus group panelists were selected. This poor reporting might erode credibility in this type of research and limit its repetition [60].

Consequently, in this study we strengthen the completeness, transparency, and consistency of reporting the consensus technique to enhance the credibility of the recommendations created [4]. In addition, addressing the role and experience of ECMO after the end of the COVID-19 pandemic provides a chance to discuss the topics appropriately without uncertainty, unlike research published during the pandemic. Additionally, mixed methods of the modified Delphi and NGT were interesting and strengthened the work.

Limitations: First, this study has the limitation of acknowledging consensus as an expert opinion tool that does not replace guidelines, randomized trials, meta-analyses, or large nonrandomized trials [61]. However, it is not intended to replace clinical judgment or clear evidence from the literature. Second, the panel failed to make recommendations on important topics in the field of ECMO for COVID-19, such as anticoagulation, cost of the service, solutions for the chain of supplies during a pandemic, pregnancy, immunocompromised cases, ECMO provider’s team models during the lack of resources, and ECMO in pediatric and neonatal patients for COVID-19. Third, the scientific committee lacked proper faculty diversity to include global ECMO communities, and some of the meetings were conducted at ELSO-related conferences, which may be received as a conflict of interest.

## Conclusion

Three years after the onset of COVID-19, our understanding of the role of ECMO during pandemic situations has evolved. However, it is incomplete. Tota14 statements reached a consensus; represent the expert opinion and available evidence in the literature, included in four domains discussing patient selection, clinical ECMO management, operational and logistic ECMO management and ethics to guide the current and next-generation ECMO providers during future pandemic situations.

## Supplementary Information

**Additional file 1:** Expert consensus statements’ meeting minutes, validation of the questions, statement formulation and analysis of the faculty's responses.

## Data Availability

Data from this study are available upon request. Dr. Rabie had full access to all the data in the study and took responsibility for the integrity of the data and accuracy of the data analysis.
