# Supplementary material for: Expert consensus statement on venovenous extracorporeal membrane oxygenation ECMO for COVID-19 severe ARDS: an international Delphi study
Source: Ann Intensive Care. 2023 May 2;13:36. doi: 10.1186/s13613-023-01126-9 (PMC10152433; doi:10.1186/s13613-023-01126-9)

**Supplementary 1**

**ECMO for the COVID-19 pandemic: Phase I. Preliminary validation of questions obtained from experts in response to a request from the scientific committee.**

**Expert panel (consensus on ECMO for COVID-19)**

**1^st^ phase questionnaire selection**

**Introduction**

Extracorporeal membrane Oxygenation ECMO is a lifesaving modality recommended by many societies that released guidelines during the current pandemic. However, ECMO provision is a challenge, especially with the constraint of resources and increases in the demand that necessitates the judicious allocation of resources which, in turn, has its impact on patient selection. The SWAAC-ELSO chapter conducted round table meetings of experts as phase III face-to-face and lived conf for optimum utilization and proper utilization of unique, labor-intensive, and costly services. Web-based at SWAAC-ELSO conference 2022 will discuss debatable concerns and questions that need to be answered about ECMO for COVID-19.

Please review the following list of proposed questions gathered from all authors by email and rate their priority for the consensus discussion using a 1-to-5-point scale that indicates the importance of the question to be included in the consensus. "1" means the highest priority, "5" means the lowest priority, for example, addressing a more frequent and/or more severe problem, an area of high care variability through greater uncertainty/controversy, or where unnecessary interventions are resulting in increased cost. Your scores will help identify the most important questions to include. The main objective of this document is to highlight the most important topics that need to be discussed. Your opinion and clinical practice experience are very important for the success of this consensus.

**Patient selection**

1. What is the maximum day’s number of mechanical ventilation high settings prior to ECMO? 1 2 3 4 5
2. Do you consider NIV or HFNC duration prior to ECMO

If yes, how many days is a cut-off? 1 2 3 4 5

1. Do you consider age on patient selection for better outcomes?

If yes, what is the cut-off? 1 2 3 4 5

1. In case of the surge with tight bed capacity, what is the cut-off for age?

If yes, what is the cut-off? 1 2 3 4 5

1. During the surge with tight bed capacity, would you utilize age to periodized patients?
2. 1 2 3 4 5
3. Prone position hours prior to ECMO? 1 2 3 4 5
4. Should we follow EIOLIA criteria and consider safe ventilators settings (or) utilize ECMO as rescue therapy to maintain oxygen saturation regardless of ventilator settings?

1 2 3 4 5

1. For the patient who partially responds to prone, should we offer him ECMO? when

1 2 3 4 5

1. Should immunomodulators or any other different medical interventions for COVID affect the decision-making for patient selection? 1 2 3 4 5
2. As mortality is increasing, how to adjust our criteria for selection and contraindication? 1 2 3 4 5
3. Awake ECMO use prior to intubation. Feasible or not feasible.

1 2 3 4 5

**ECMO management**

1. Role of steroids and immunomodulators after ECMO initiation.

1 2 3 4 5

1. What is the optimal level of anticoagulation, optimal anticoagulation, optimal way of measuring? 1 2 3 4 5
2. Would you consider extubation first and awake ECMO to enhance early recovery?

1 2 3 4 5

1. Prone positioning during ECMO as a routine will improve the outcome.

1 2 3 4 5

1. Need for conversion from VV to other modes( VAV or VA ECMO). 1 2 3 4 5
2. Tracheostomy on ECMO. 1 2 3 4 5
3. Best cannulation strategy fem-fem, fem-jag. Or fem-protek duo.

1 2 3 4 5

1. Nutrition and surveillance for fungal infections while on ECMO.

1 2 3 4 5

1. Early rehabilitation – how early? 1 2 3 4 5
2. Should we screen for DVT/PE during ECMO? 1 2 3 4 5
3. Are circuit clots more common? Oxygenator changes more frequently necessary?
4. 1 2 3 4 5
5. hemorrhagic and thrombotic complications are the same compared to the non-COVID-19 patients ECMO population. 1 2 3 4 5
6. difficulties in basic management of ECMO patients with an increasing number of non-ICU/ECMO experienced personnel working in the intensive care environment.

1 2 3 4 5

1. Lung transplant for COVID-19, Time and criteria? Early as lung will not recover or late considering late native lung recovery. 1 2 3 4 5

**Post ECMO care**

1. How do the outcomes differ? Not just mortality but disability psychological outcomes. 1 2 3 4 5
2. How are the family/surrogates impacted? 1 2 3 4 5
3. Prolonged run on ECMO. with or against. 1 2 3 4 5
4. Long-term outcomes! Would you recommend a post-ECMO clinic to follow up with the patients? 1 2 3 4 5
5. Should we screen for DVT/PE routinely after ECMO? 1 2 3 4 5
6. Would you recommend a post-ECMO clinic to follow up with the patients?

1 2 3 4 5

**Operational management**

1. Aspects of centralized model vs. institutional hub and spoke model vs. ECMO in any ICU model. 1 2 3 4 5
2. Mentoring services from ELSO for newer centers. Does it help?

1 2 3 4 5

1. Should we encourage new centers? Yes, no 1 2 3 4 5

**ECMO transportation**

1. ECMO transportation is feasible and safe for the staff regarding infection? Yes, no 1 2 3 4 5
2. What are recommendations for safe transportation for both the staff and patient?

1 2 3 4 5

**Logistic and Supply Resources.**

1. What is the cost of ECMO for COVID, especially because the runs are longer?

1 2 3 4 5

1. How do we help our staff with psychological and other support?

1 2 3 4 5

1. What is society’s responsibility to hospital workers? 1 2 3 4 5
2. How is the staff impacted? 1 2 3 4 5

**Training**

- Education/training issues during a pandemic: strategies to acquire/maintain competence and stay up to date in the field despite limitations imposed by the pandemic.

1 2 3 4 5

**Ethical dilemma**

1. Patients are refusing long-term ICU admission and being noncompliant to HFNO/NIV + refusing intubation has a high impact on the outcome once on ECMO!

1 2 3 4 5

1. If resources are scarce, how do we weigh the use of ECMO against other treatment that needs to be delivered? 1 2 3 4 5
2. When should we provide ECMO in contingency or crisis standards of care, and when should we withhold it? 1 2 3 4 5
3. Duration of ECMO support. 1 2 3 4 5
4. Definition of futility. 1 2 3 4 5

**Research**

1. Feasibility during the pandemic. 1 2 3 4 5
2. How to facilitate better data collection. 1 2 3 4 5
3. Aspects of research that need focus- are anticoagulation, prone positioning, etc.

1 2 3 4 5

Author name: ______________________________

Comments:

**
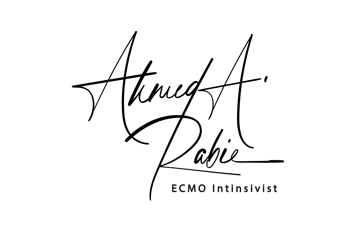
**Regards

**Supplementary 2**

**Phase II: Revalidation of the questions and analysis of the faculty's responses.**

**D1: Patient selection**


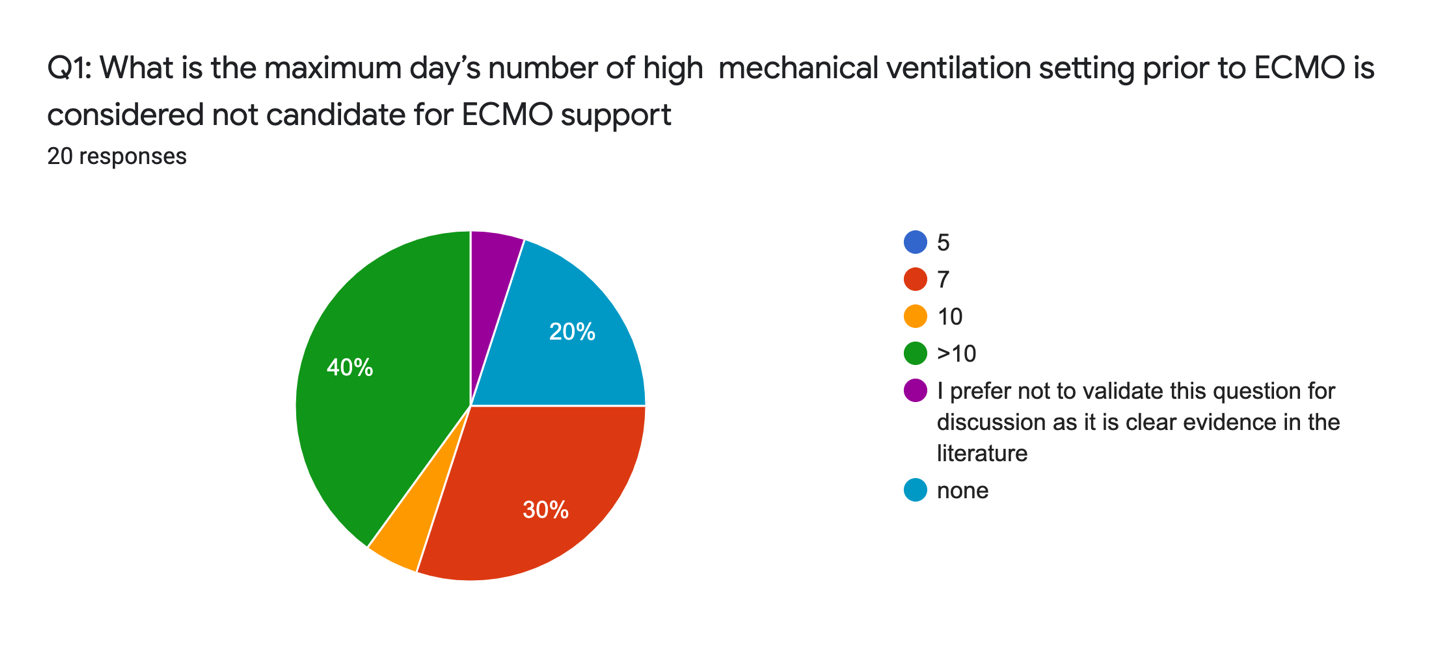


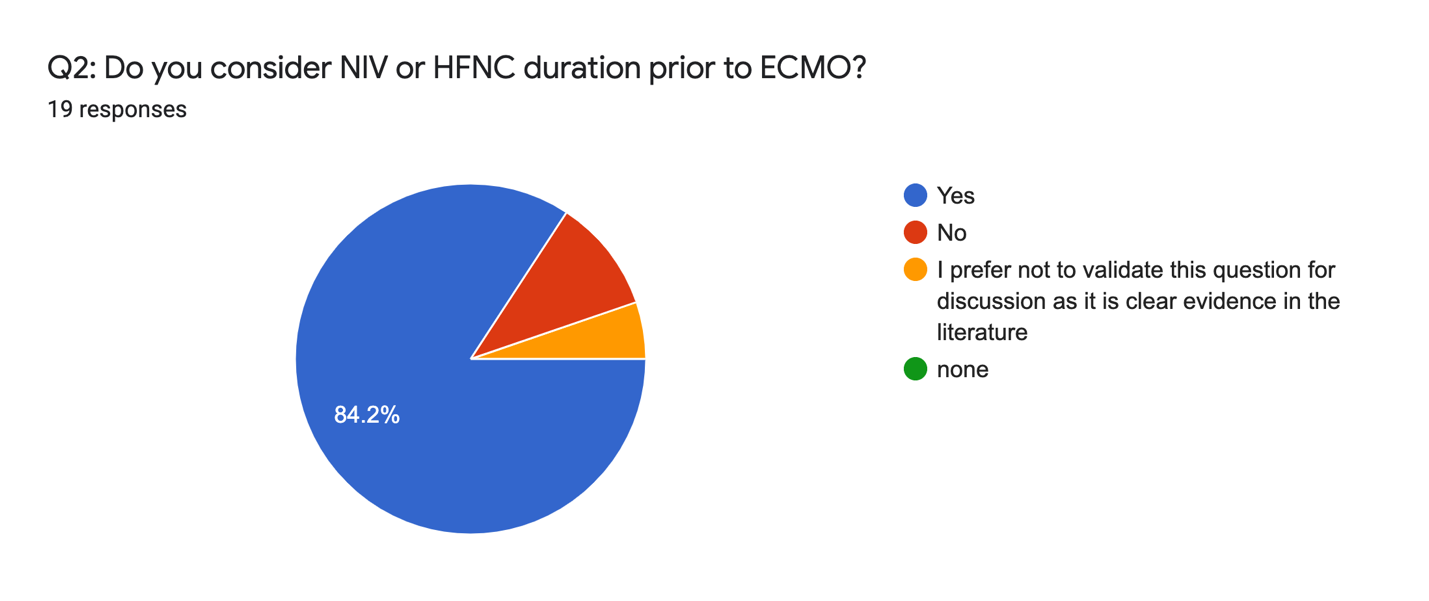


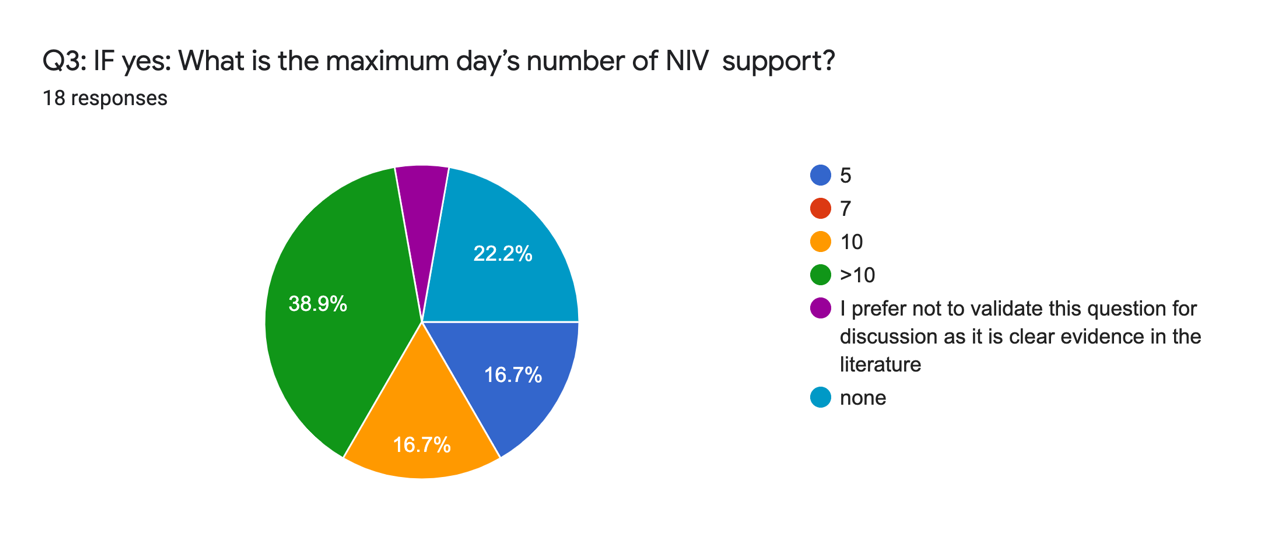


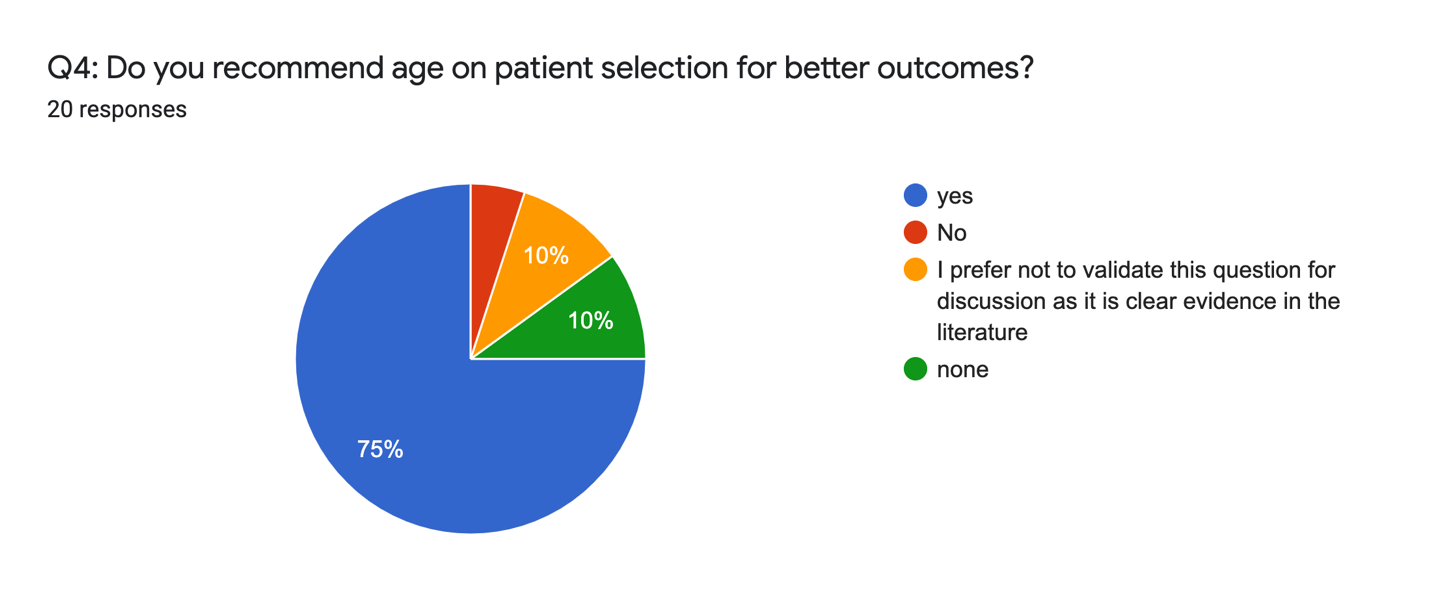


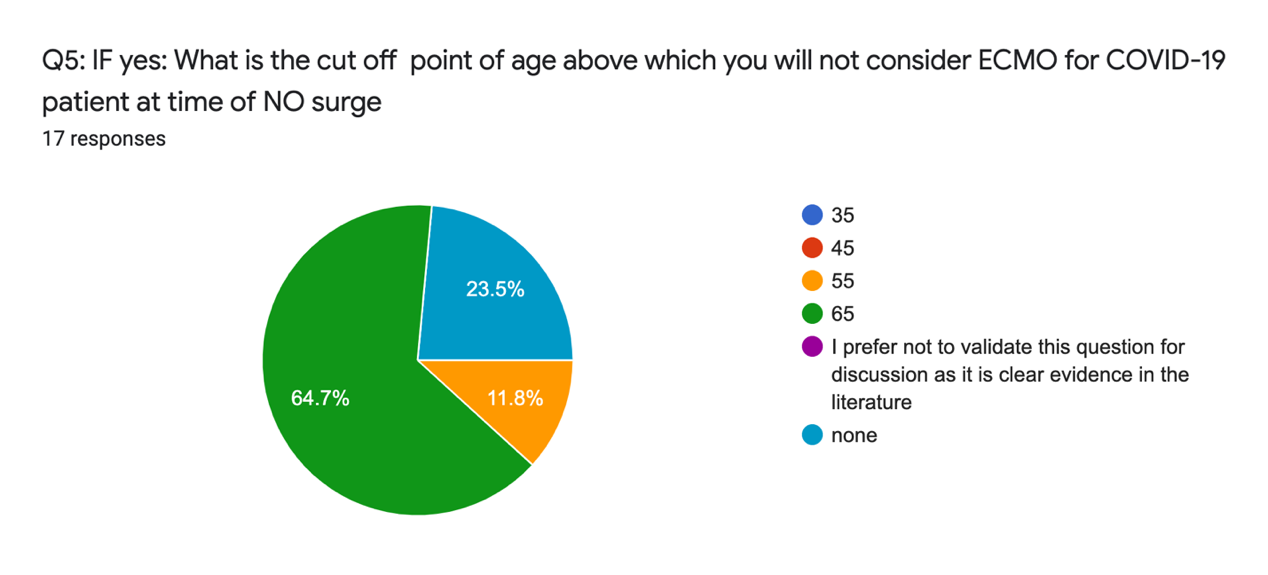


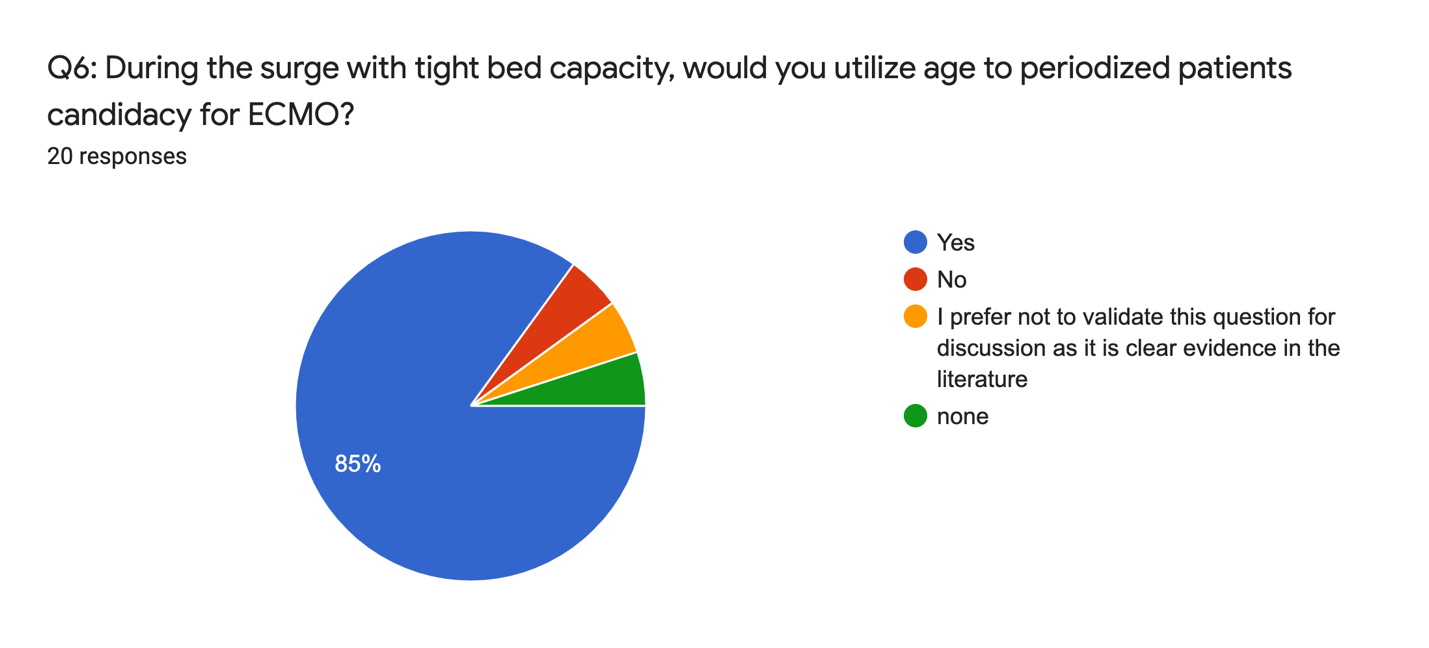


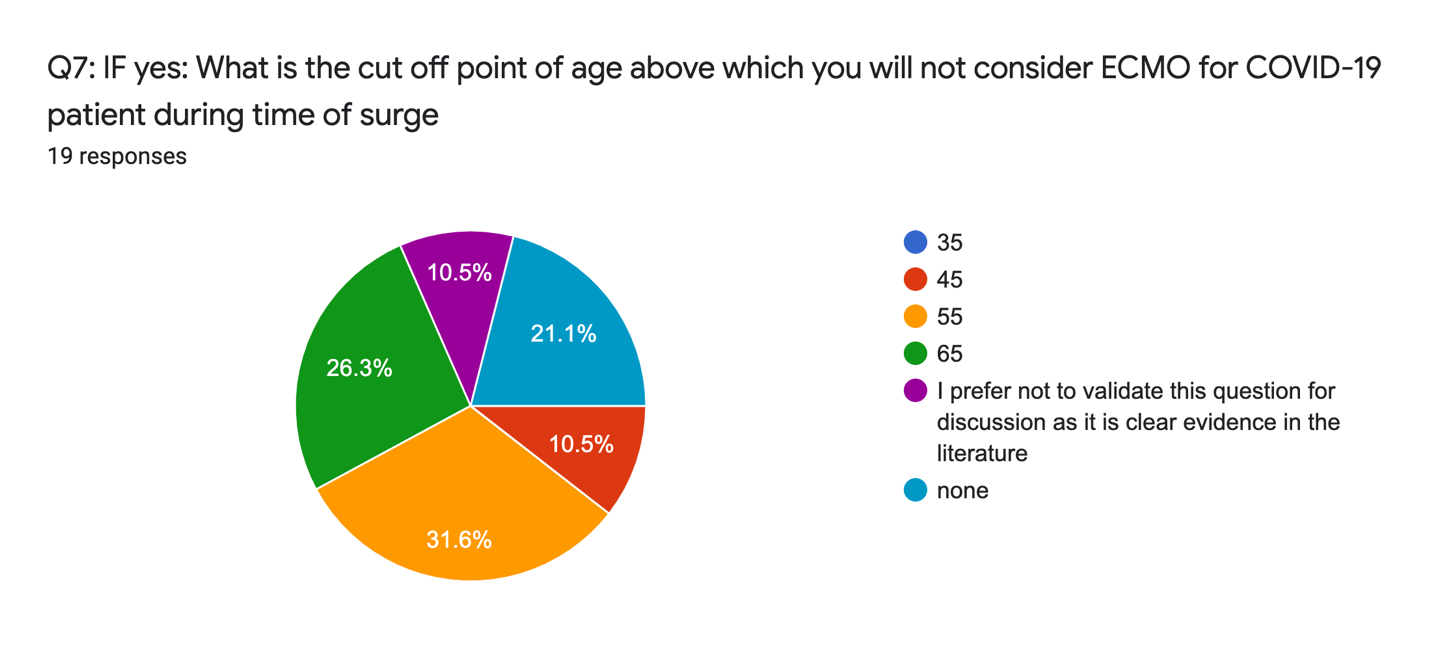


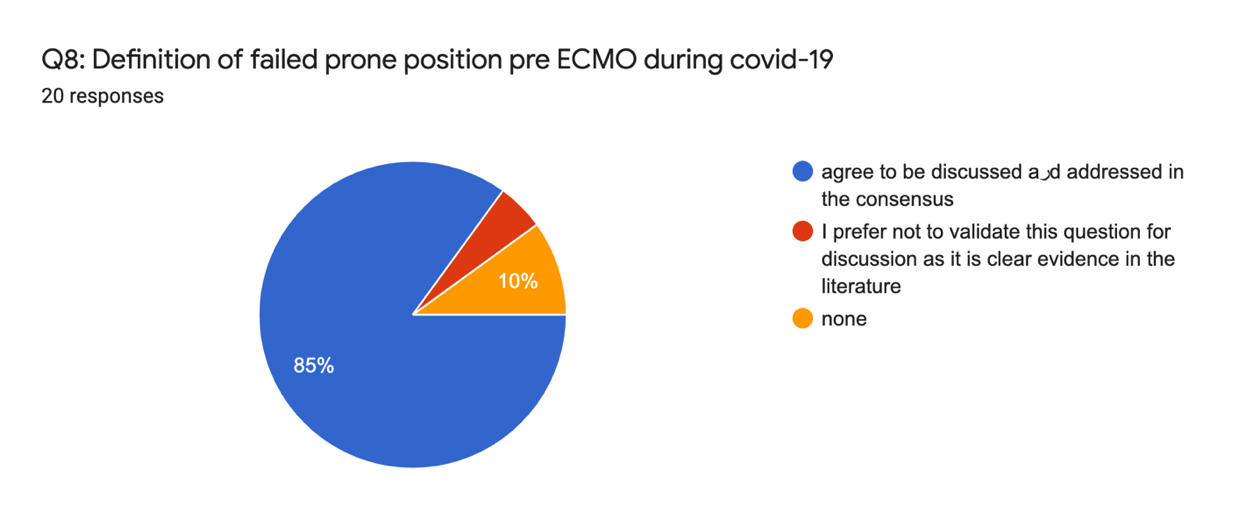


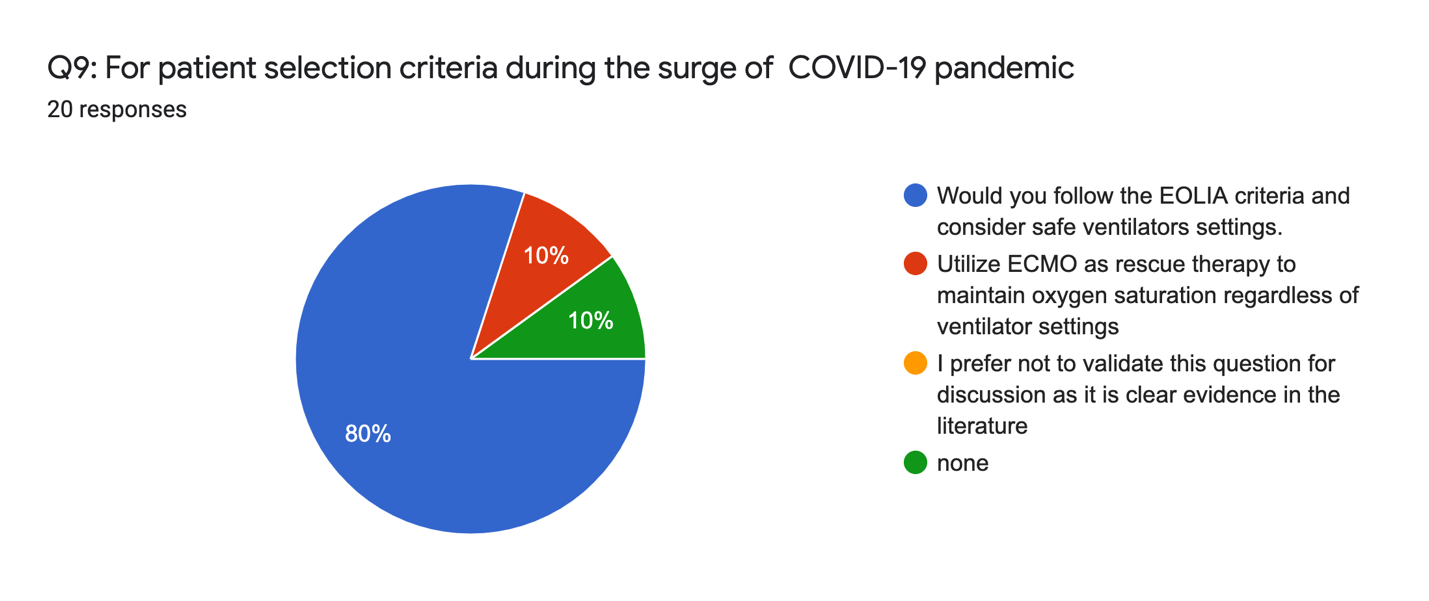


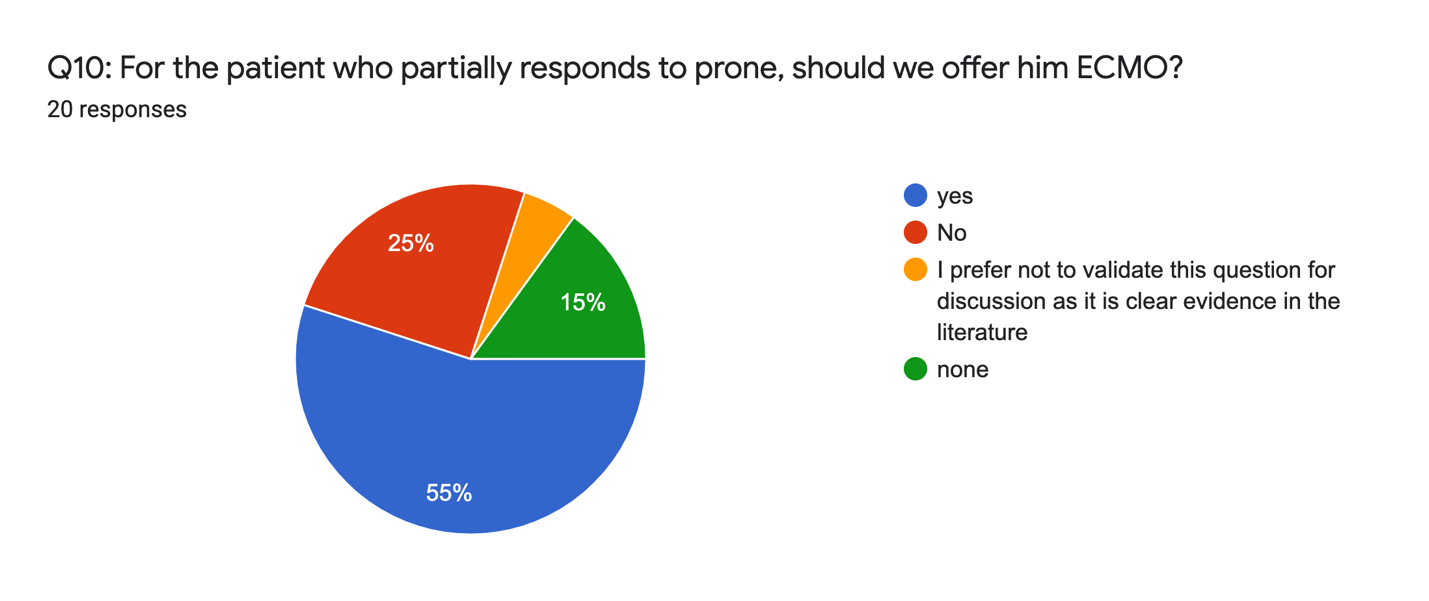


**D2: ECMO management**


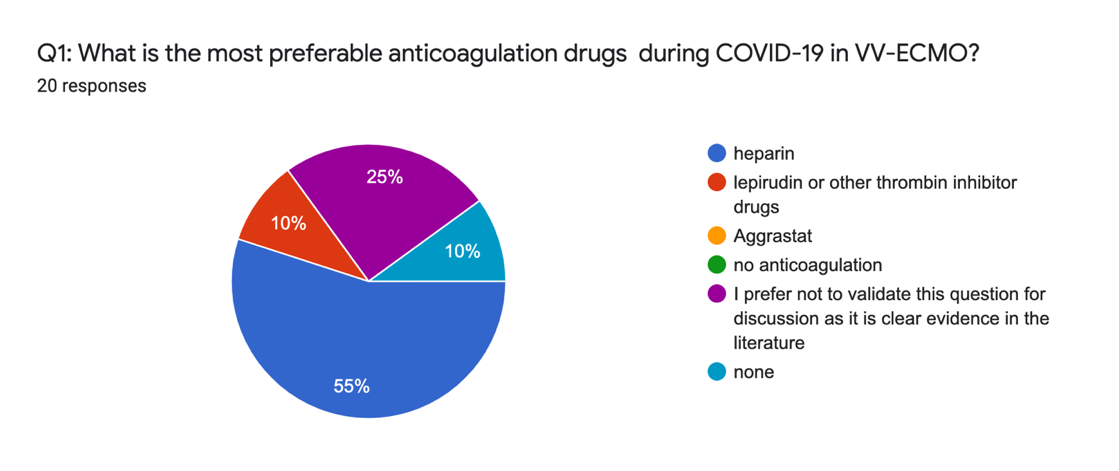


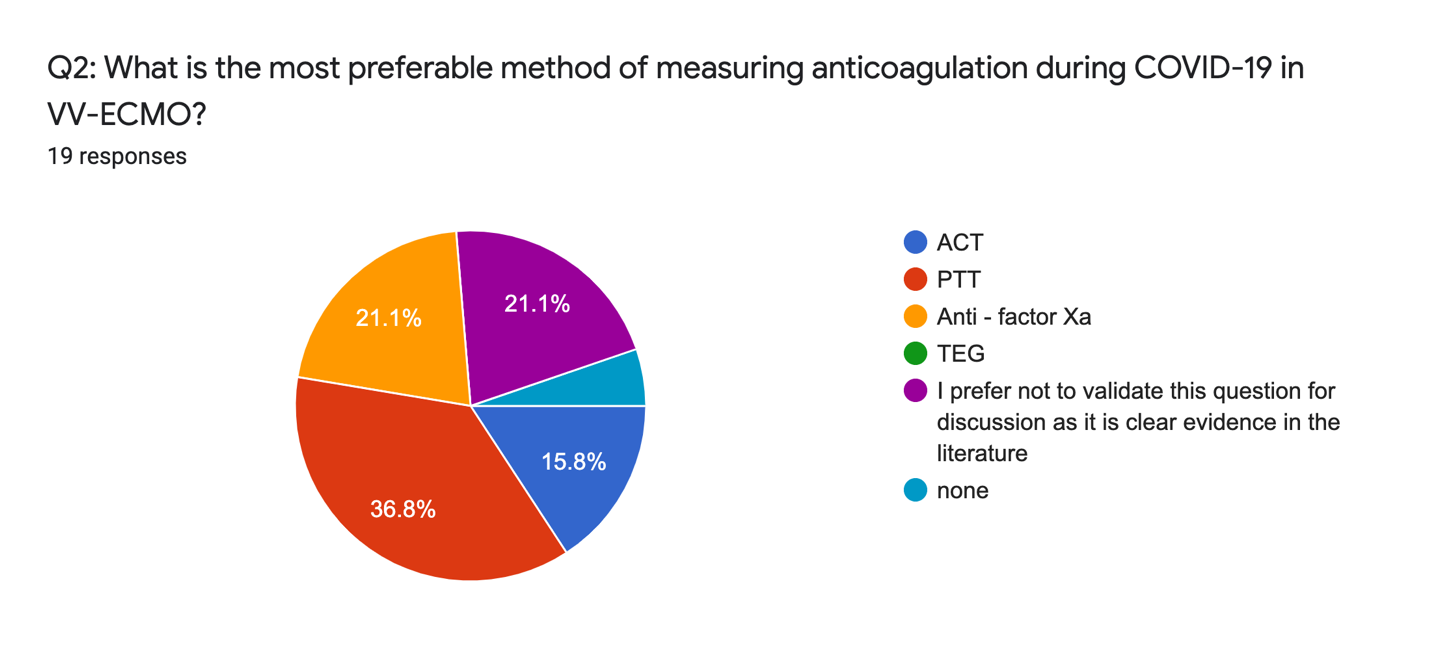


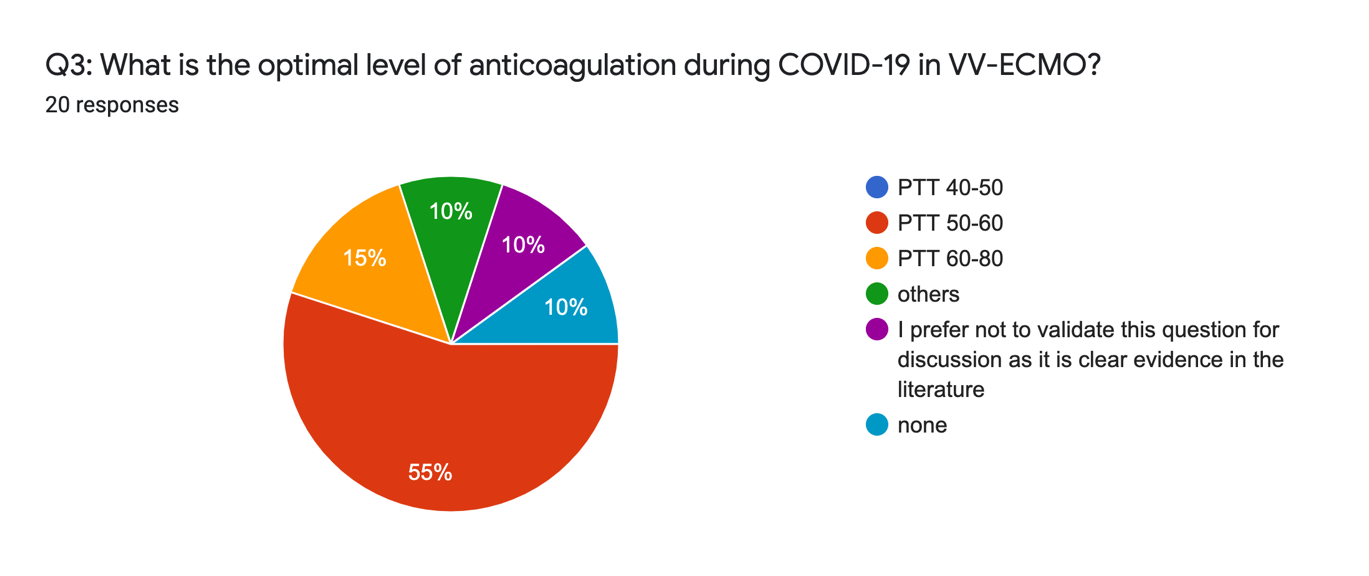


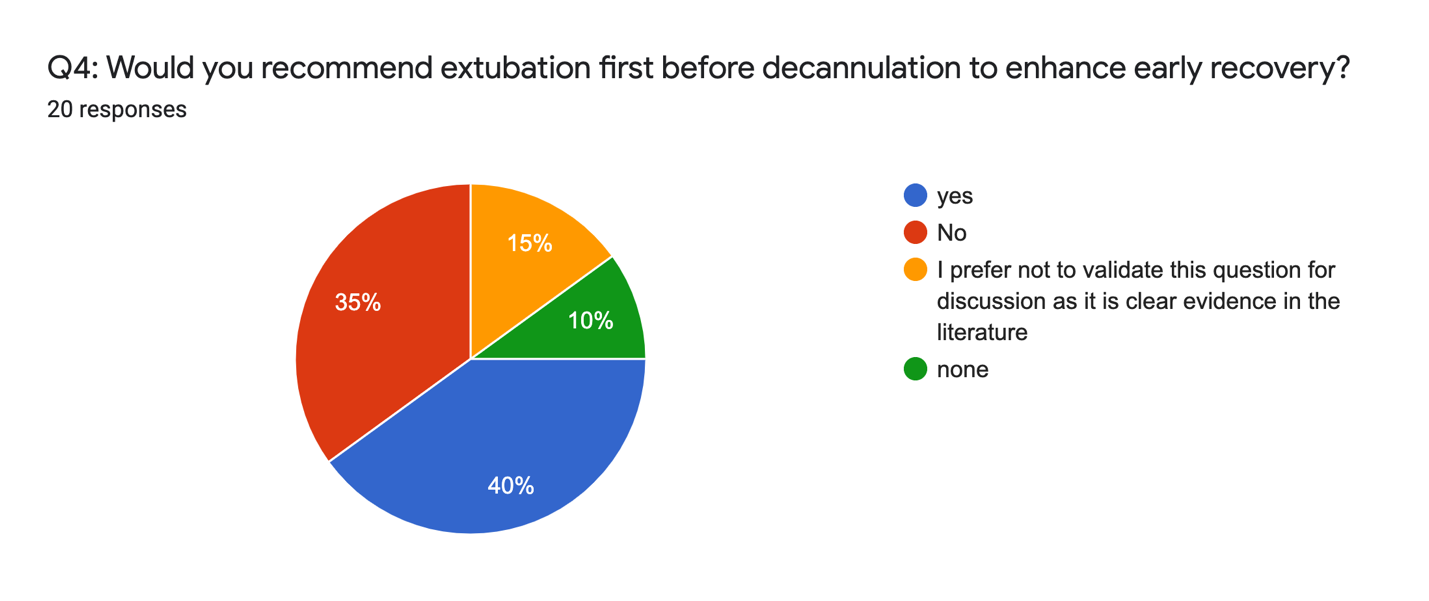


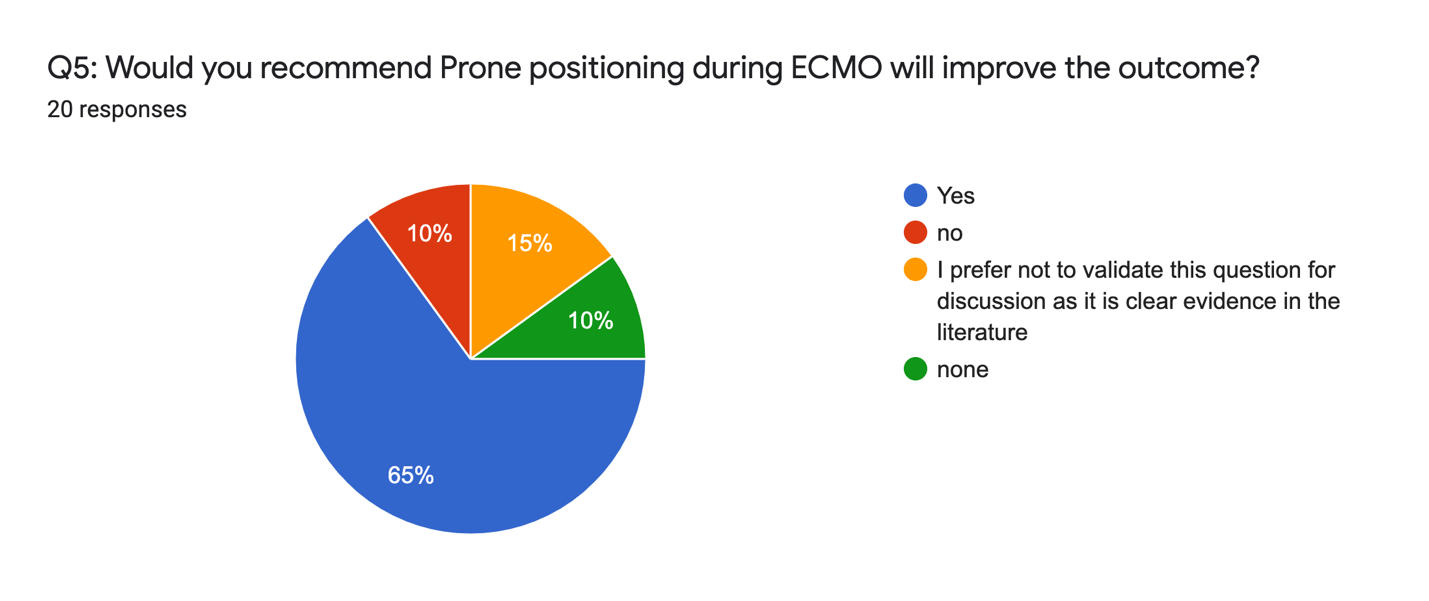


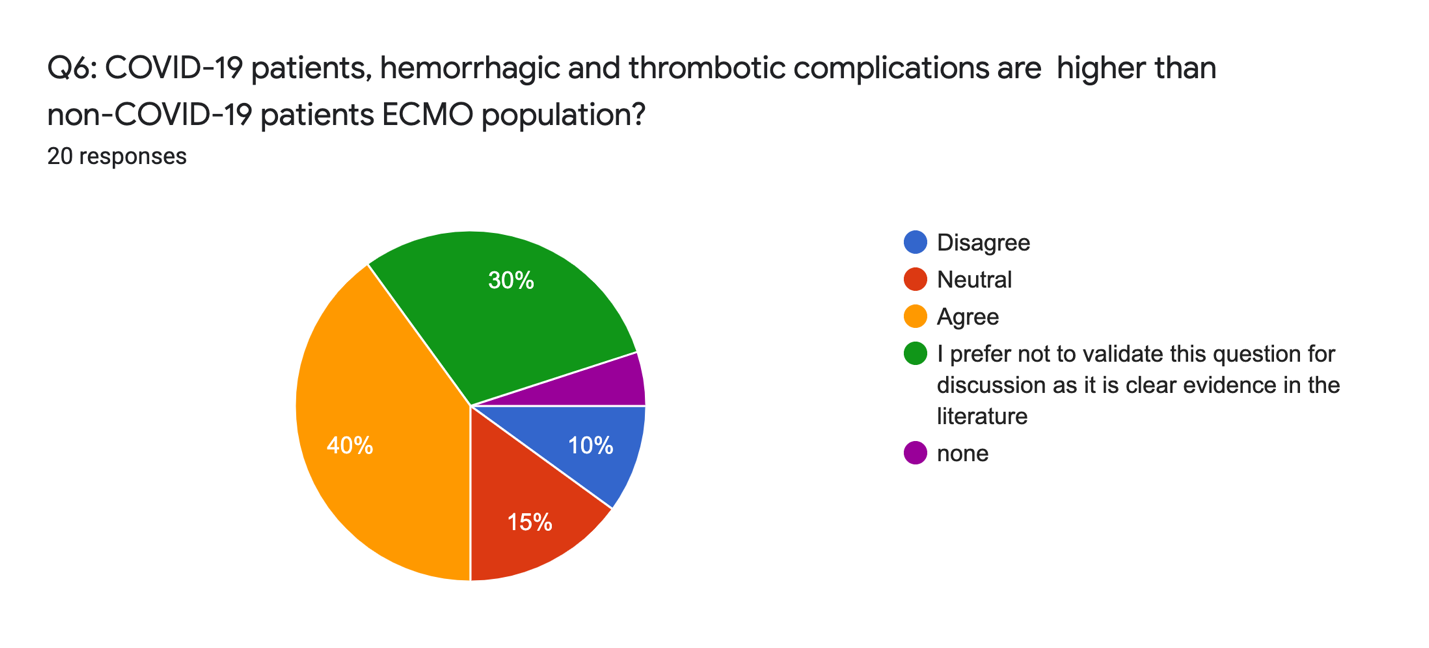


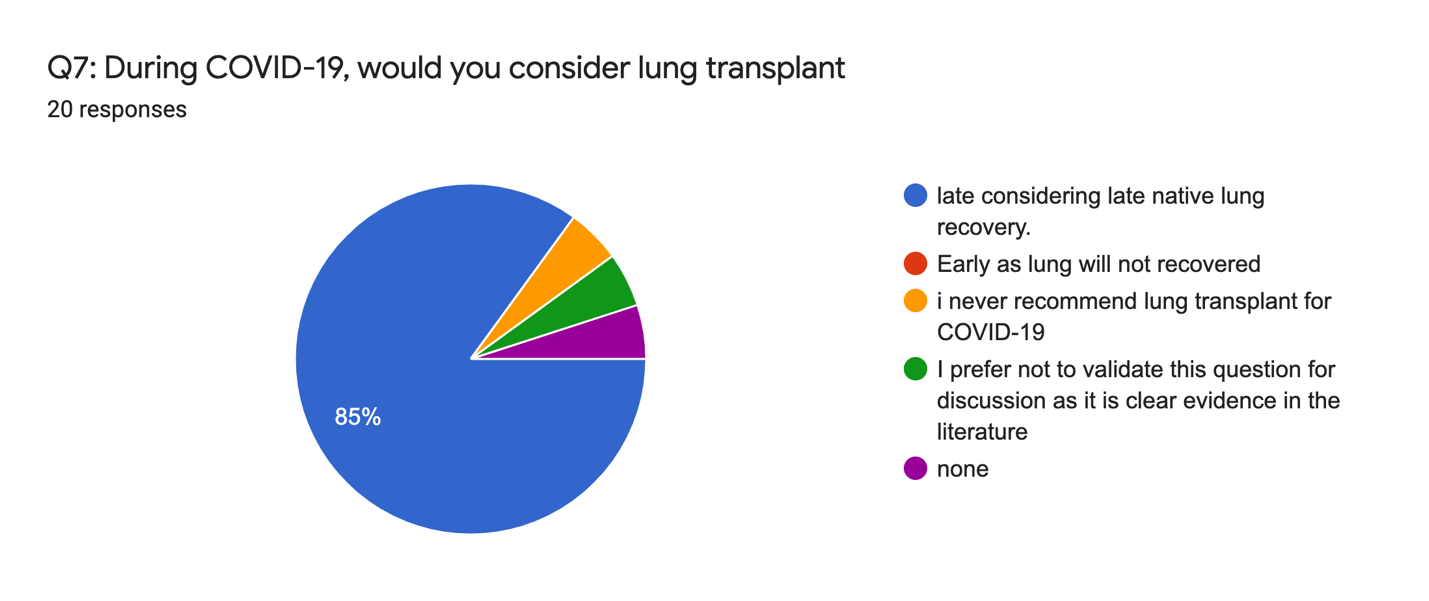


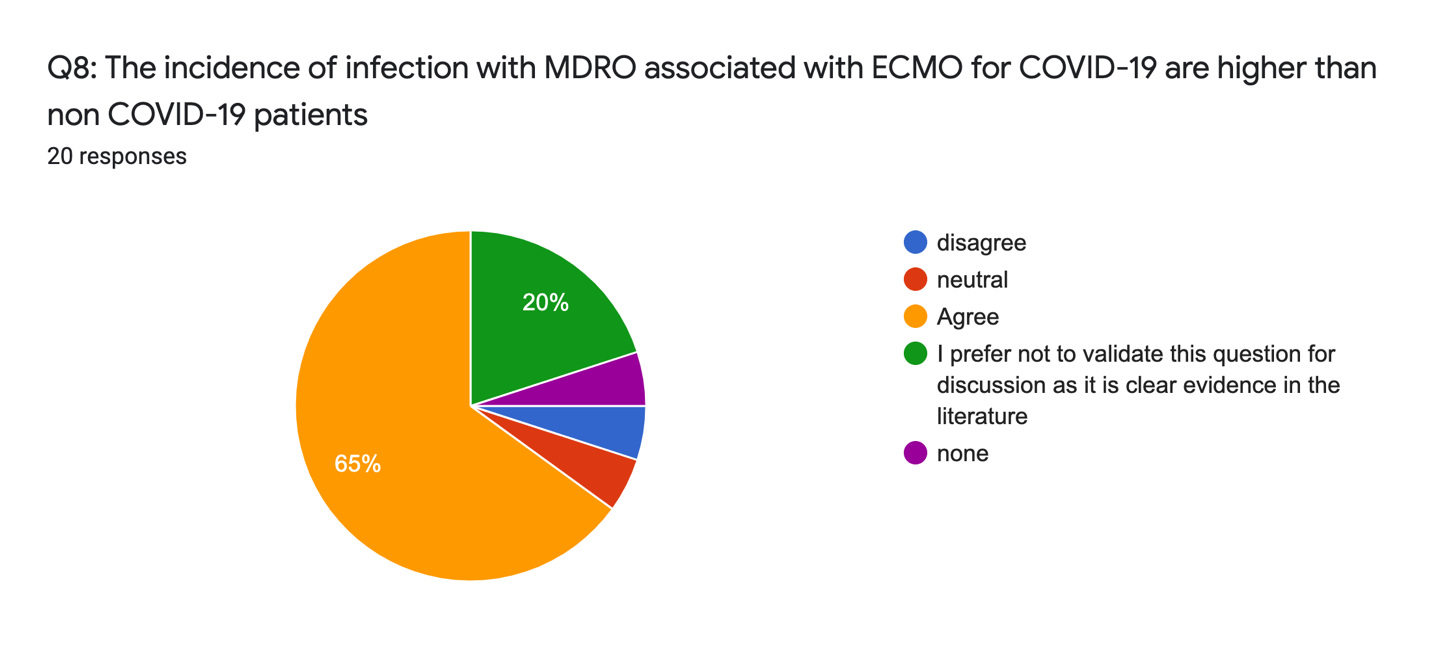


**D3: Post ECMO care**


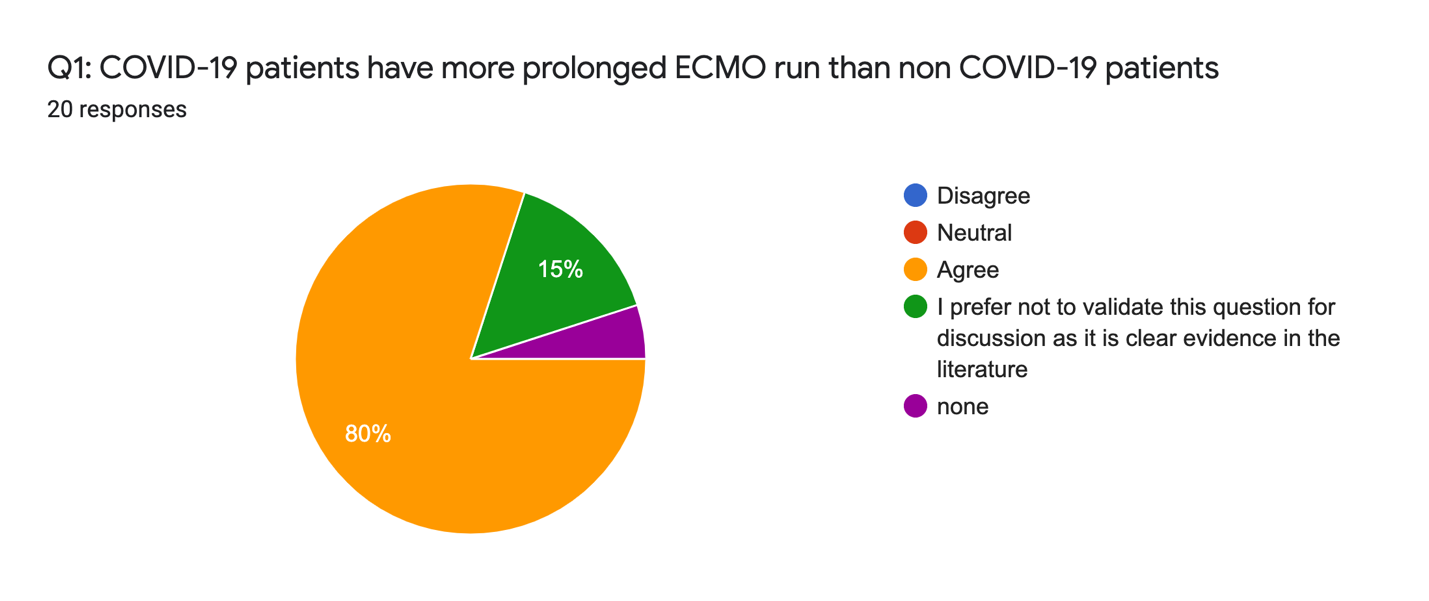


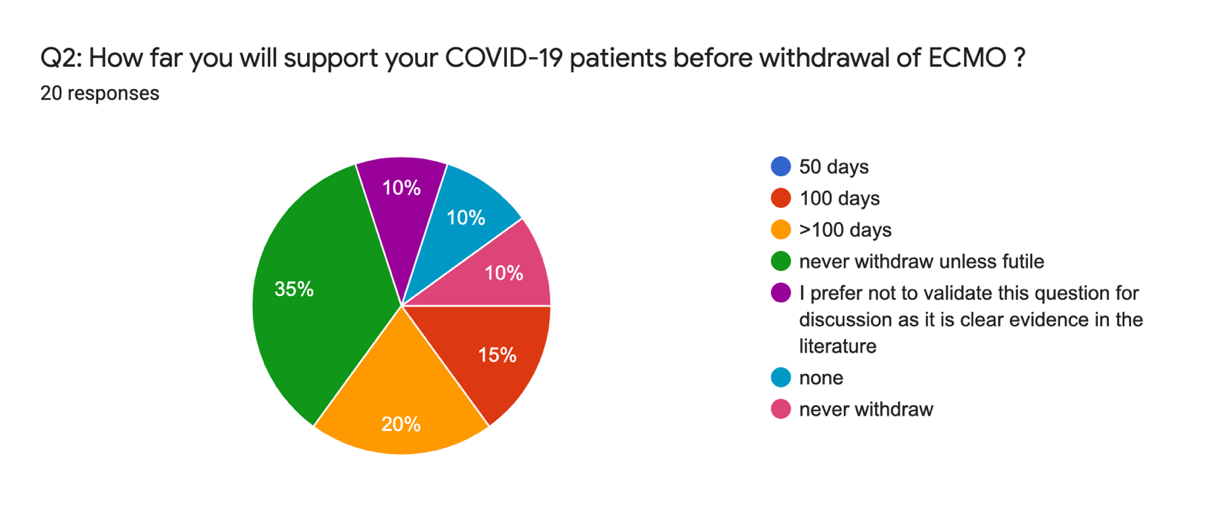


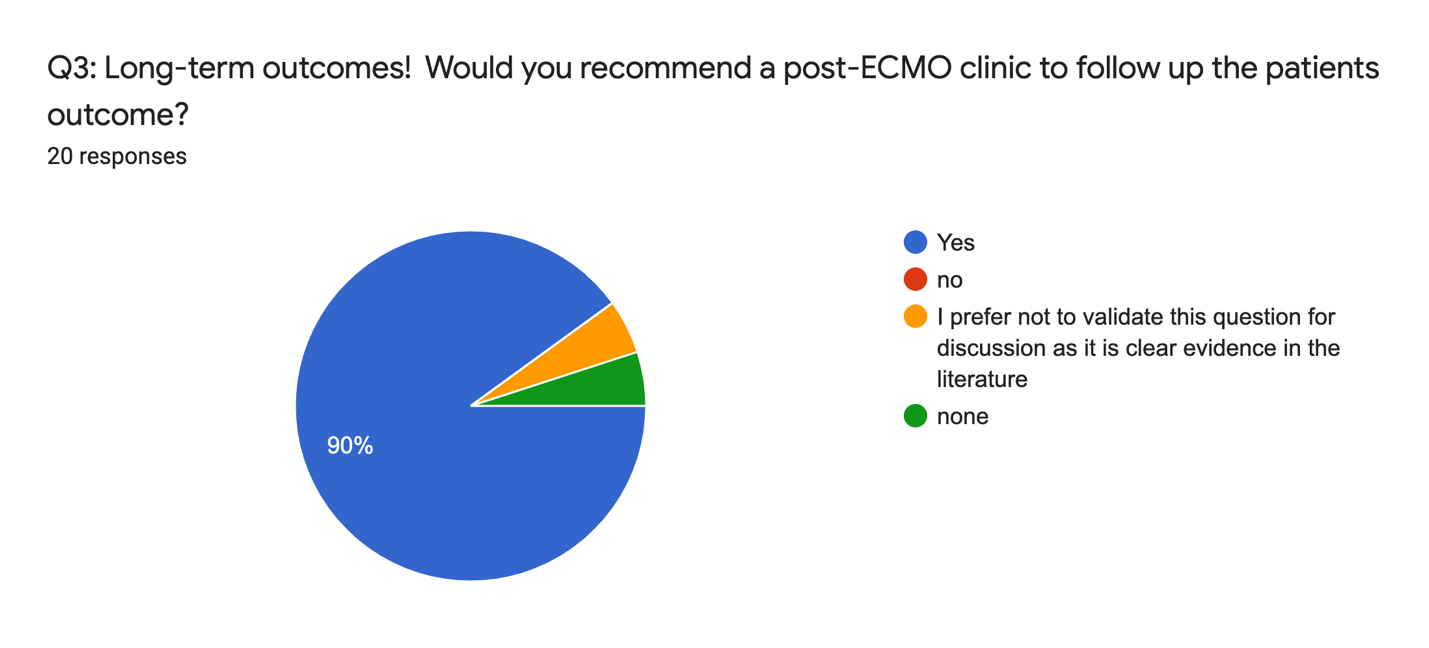


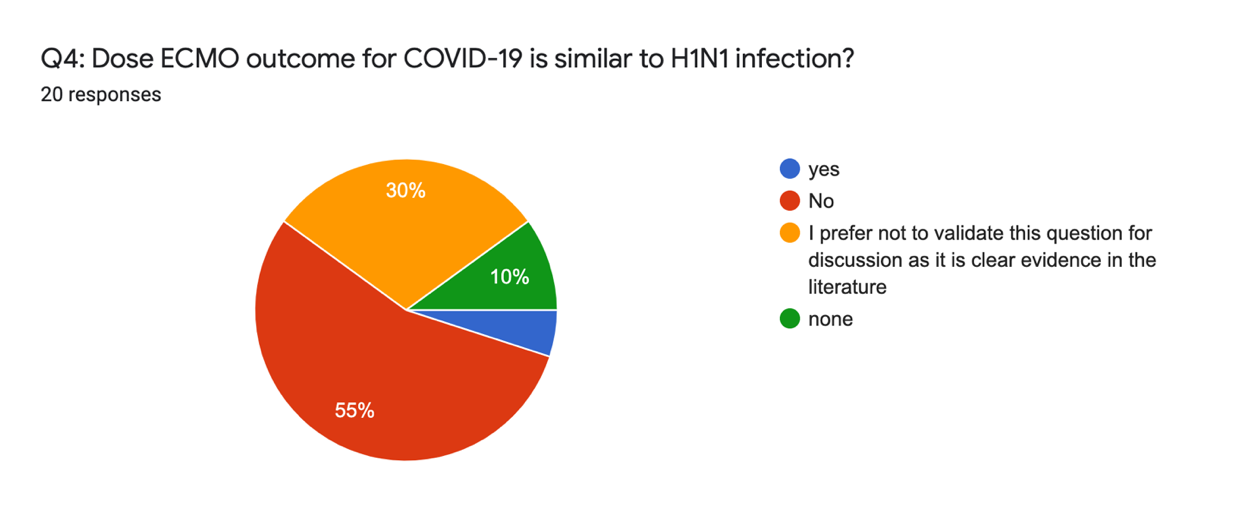


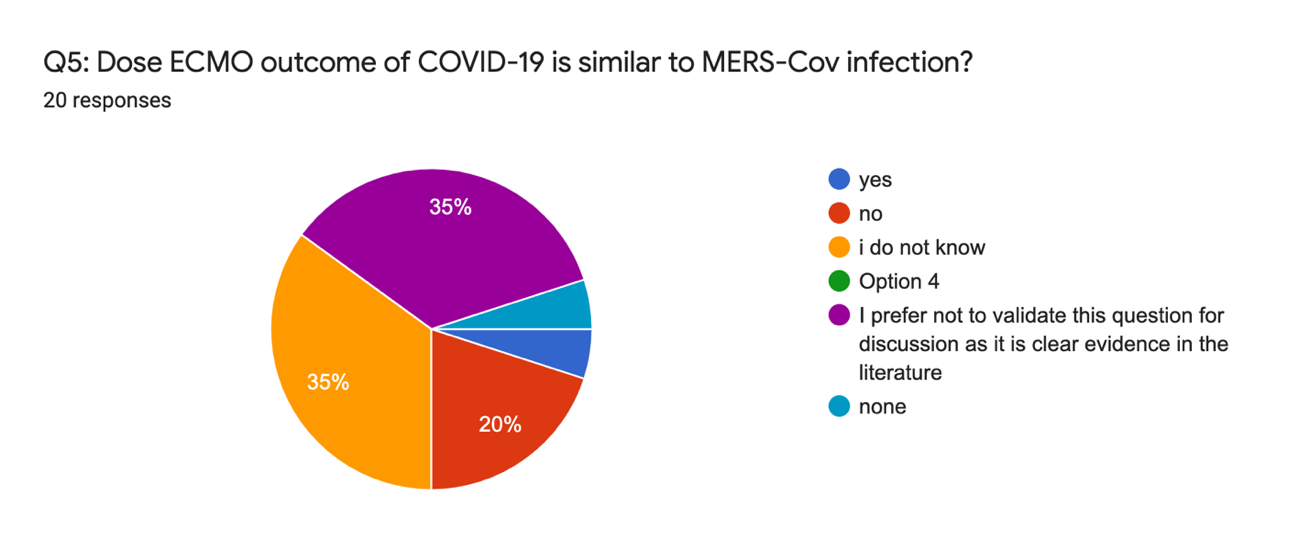


**l management**


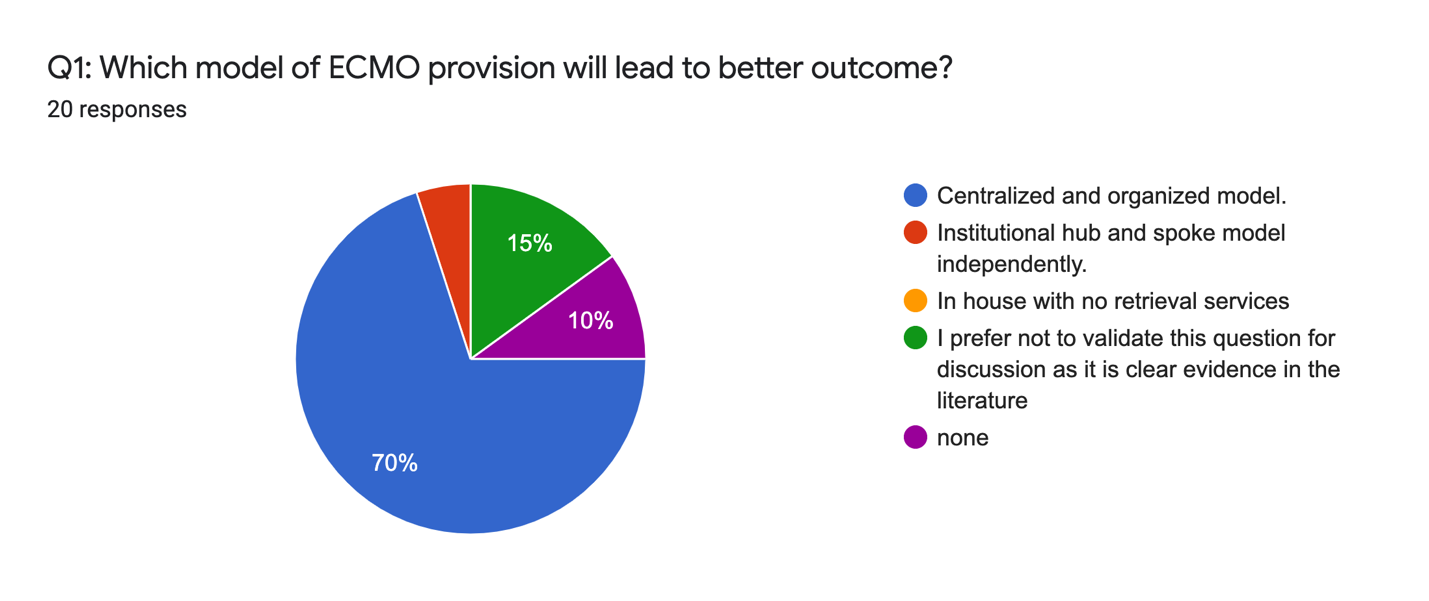


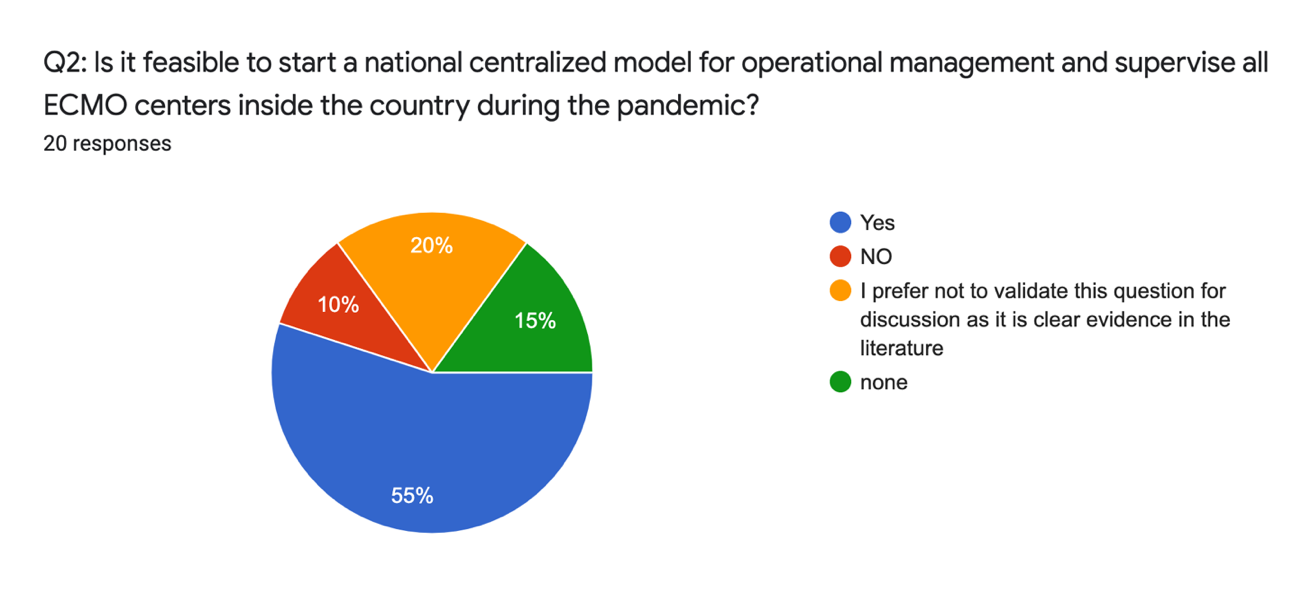


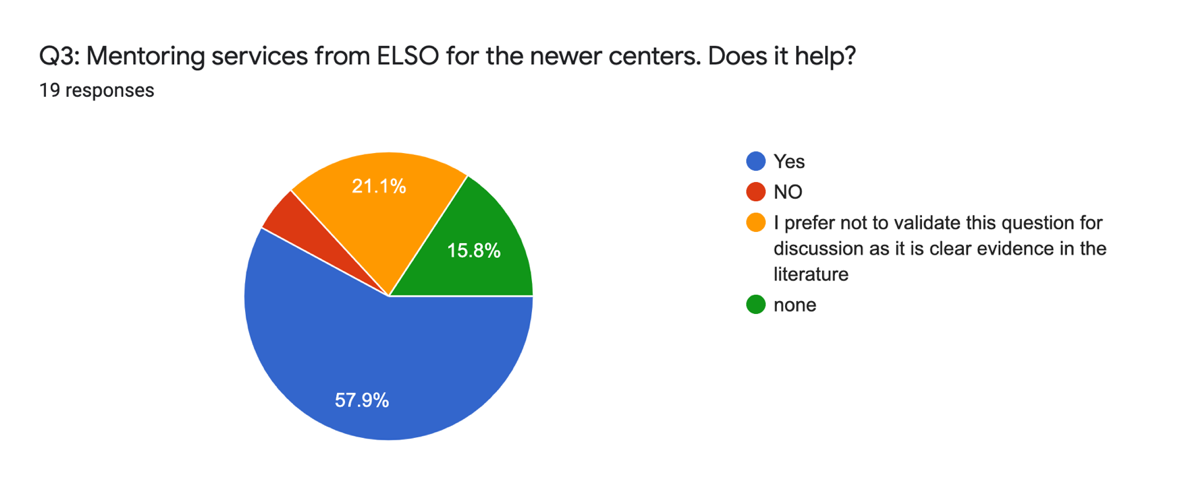


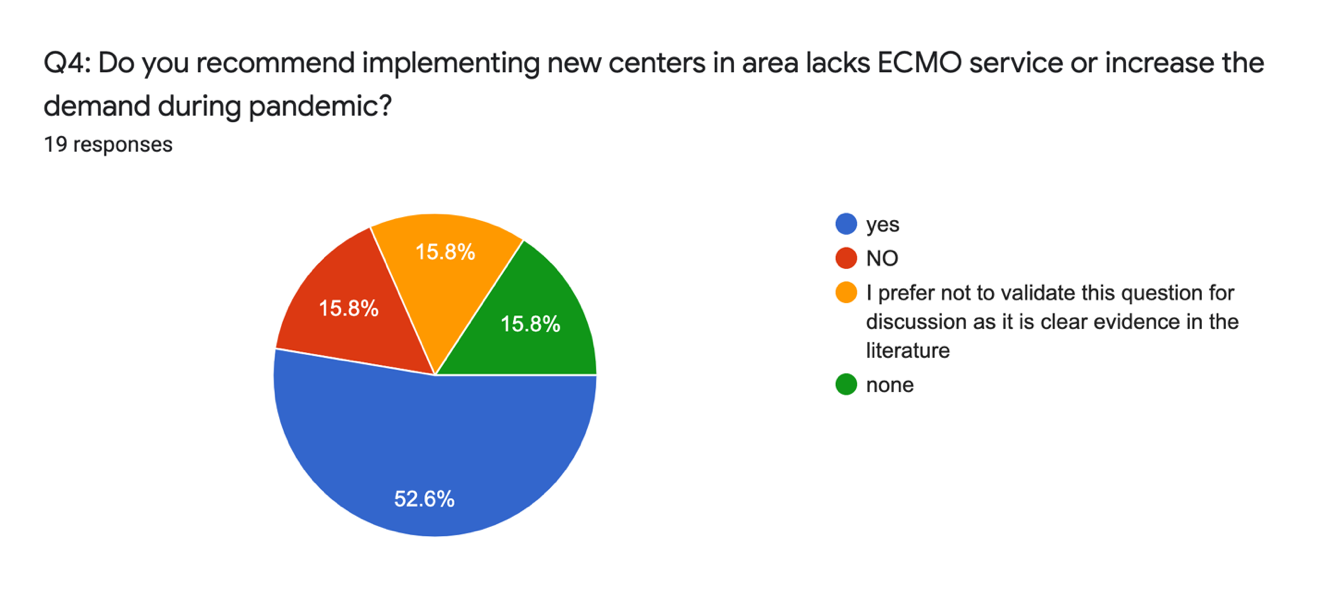


**D5: ECMO transportation**


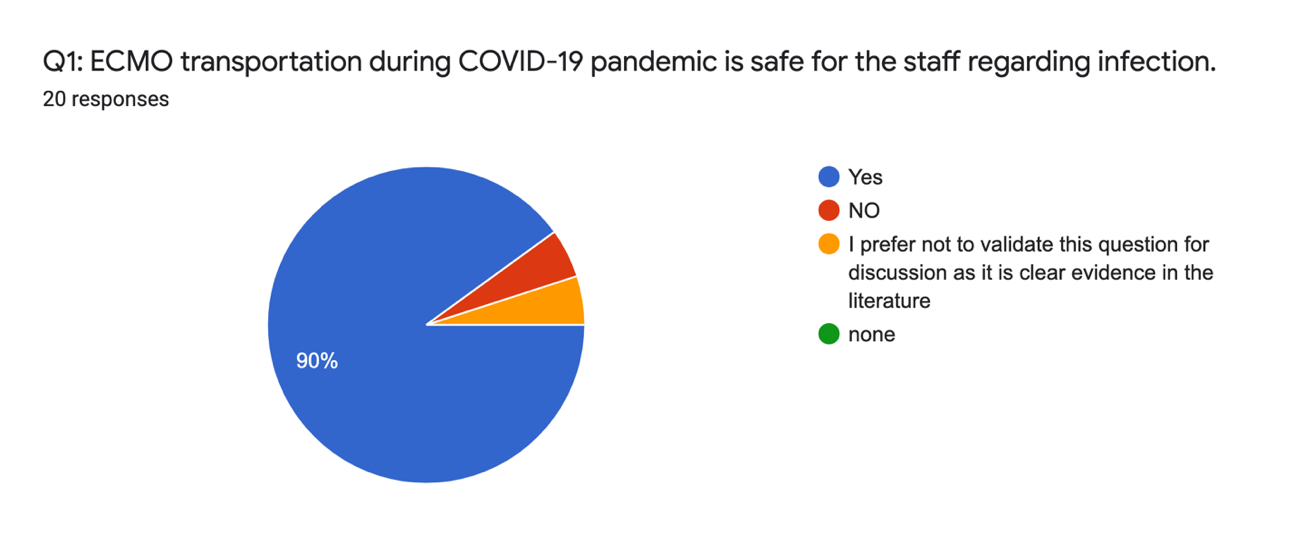


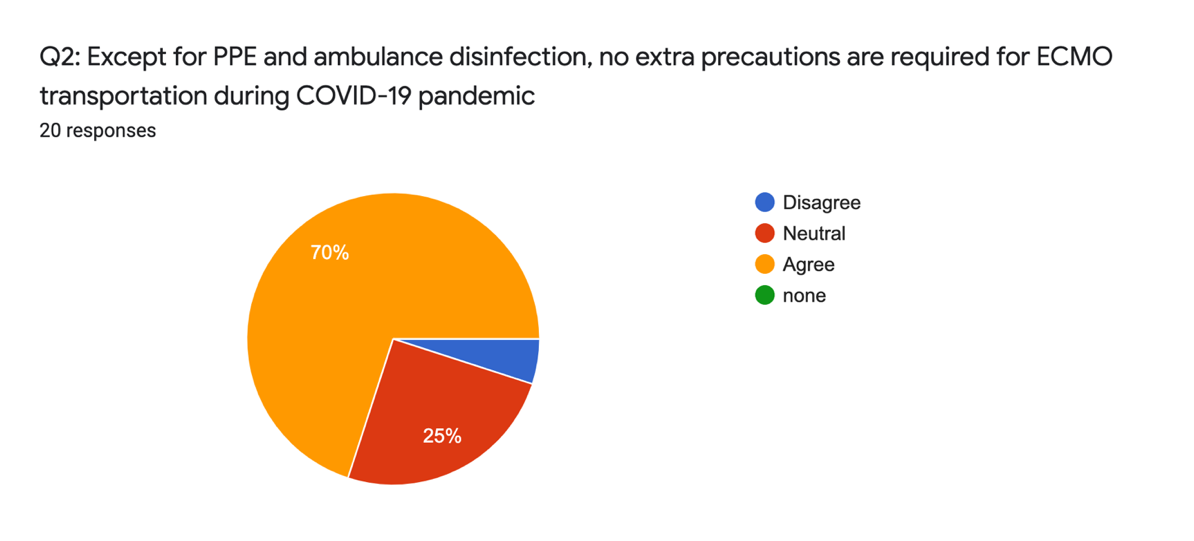


**D6: Logistic and Supply Resources.**


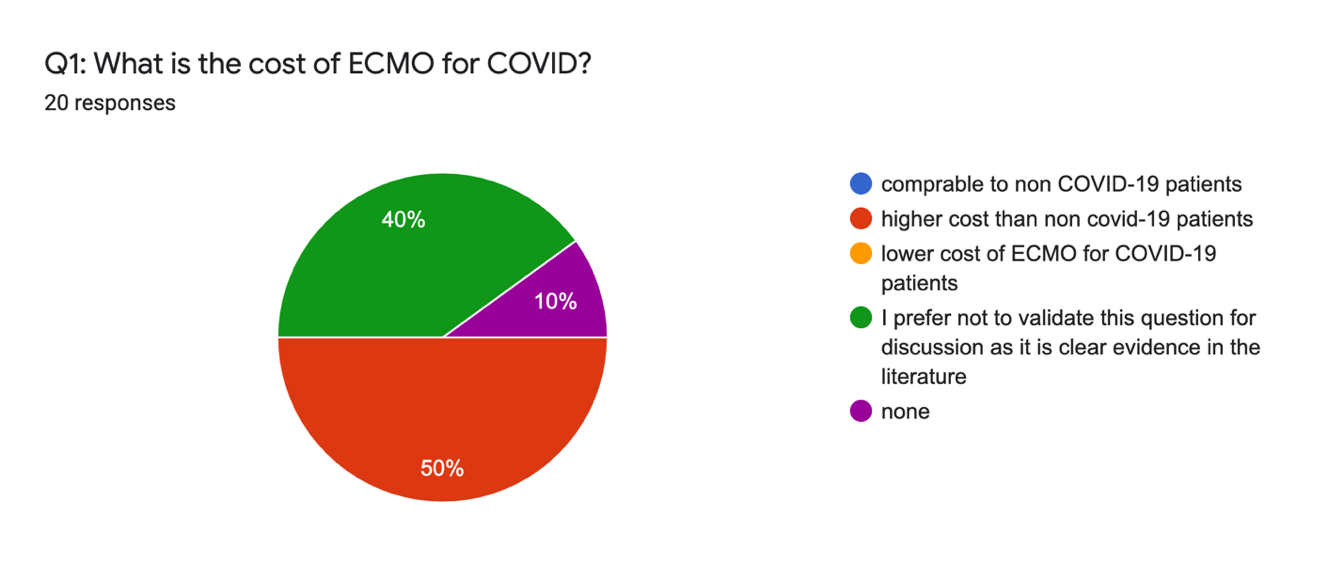


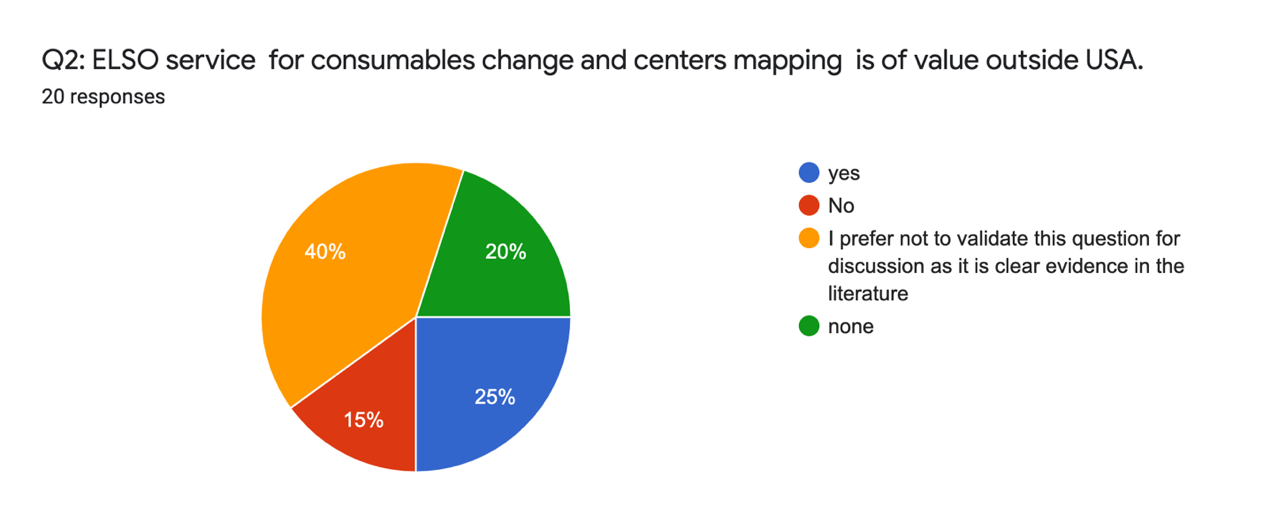


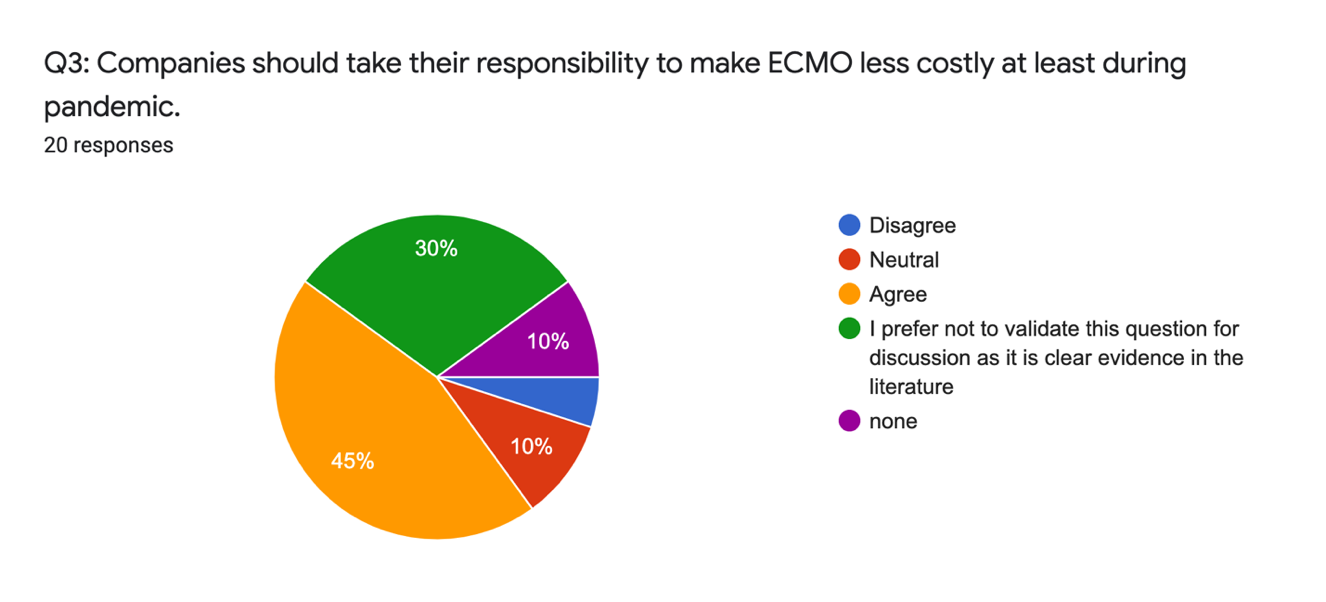


**D7: ECMO Training**


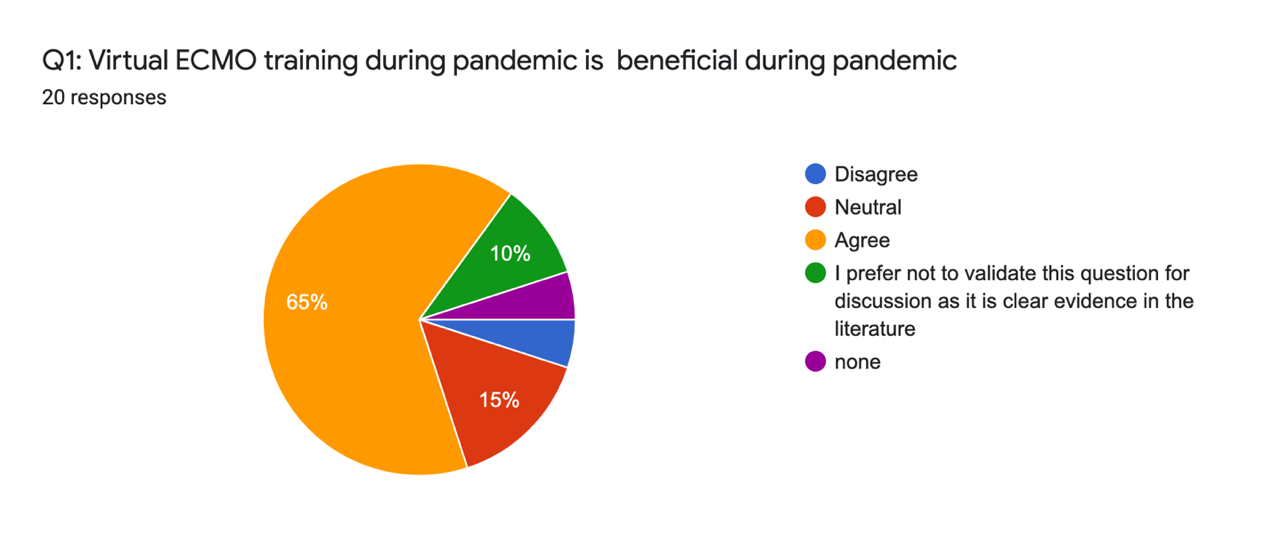
**D8: Ethics**


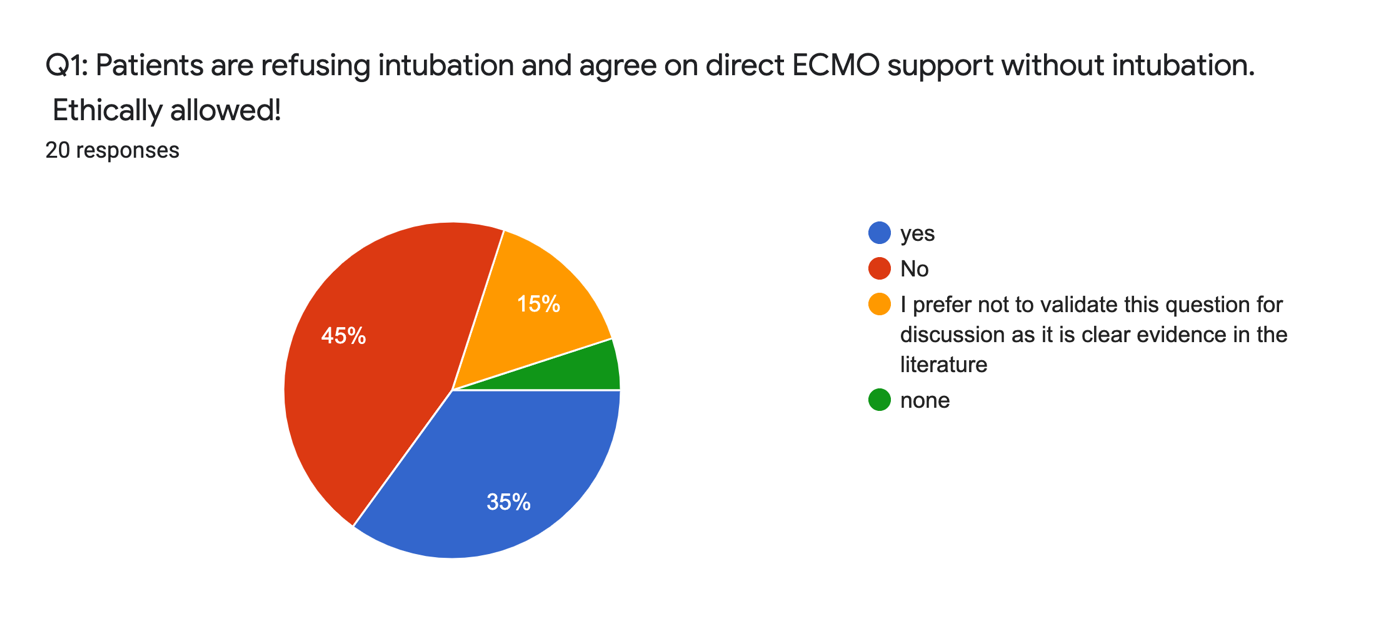


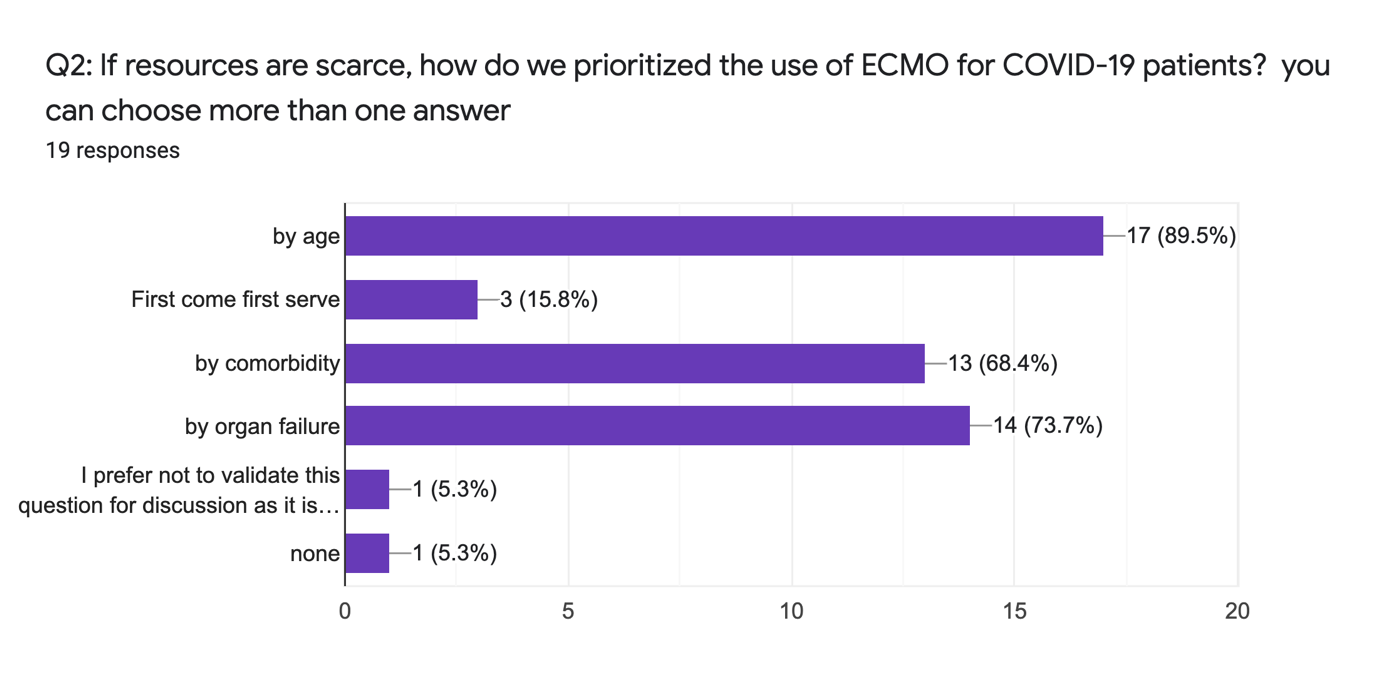


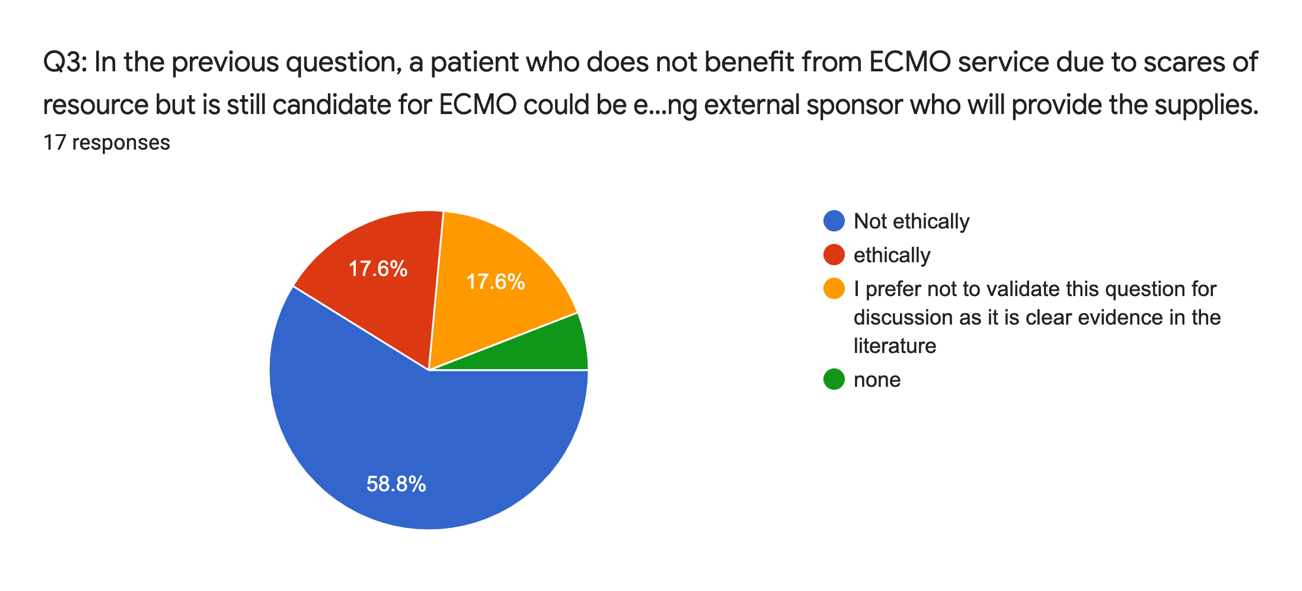


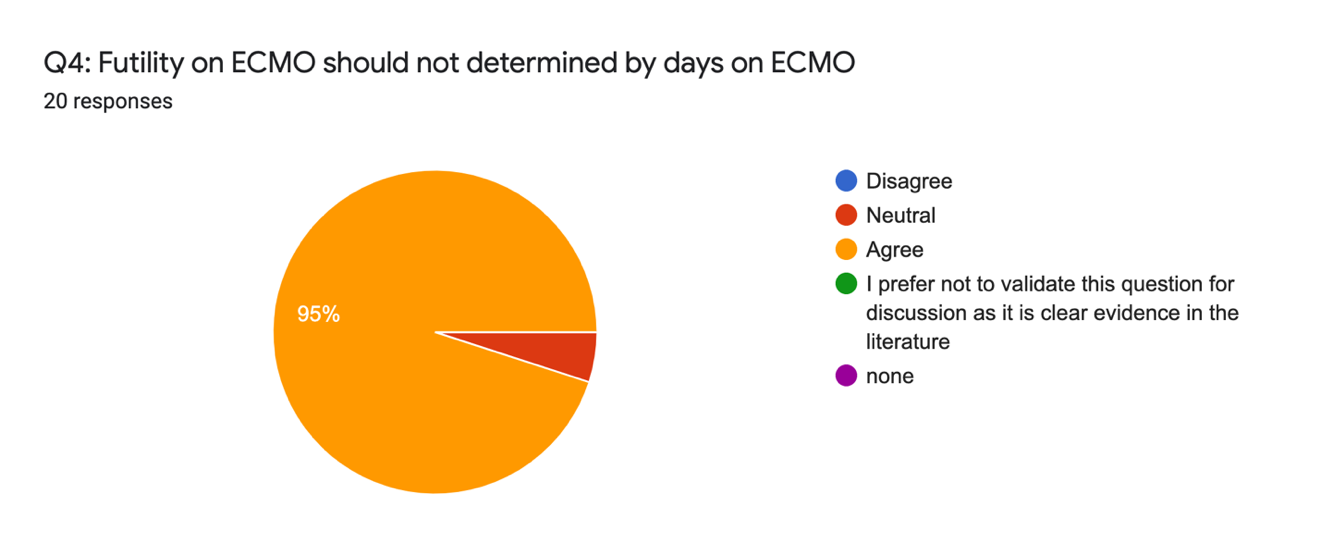


**D9: Research**


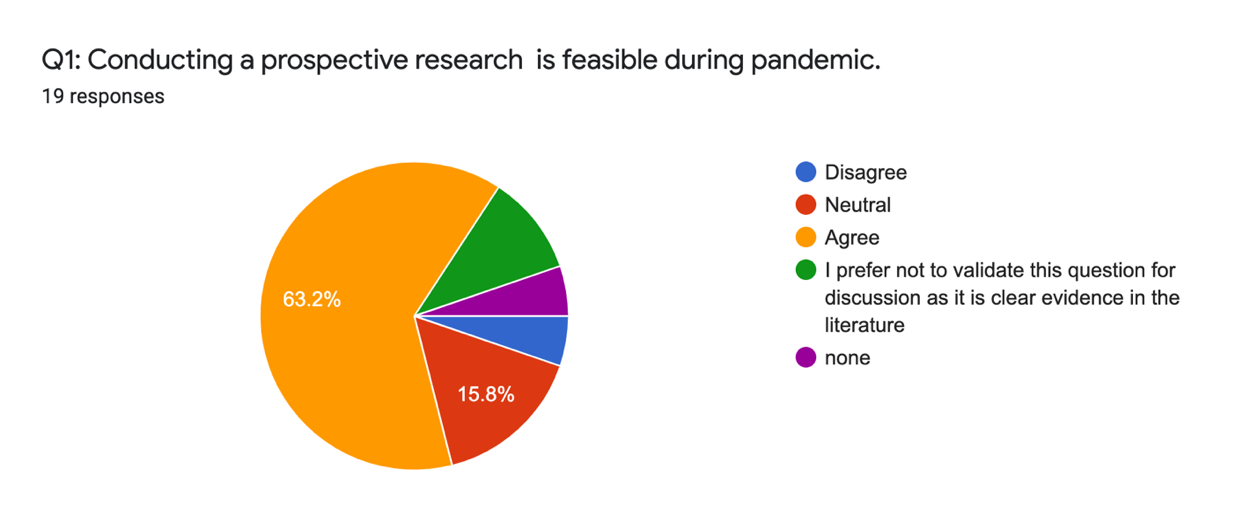


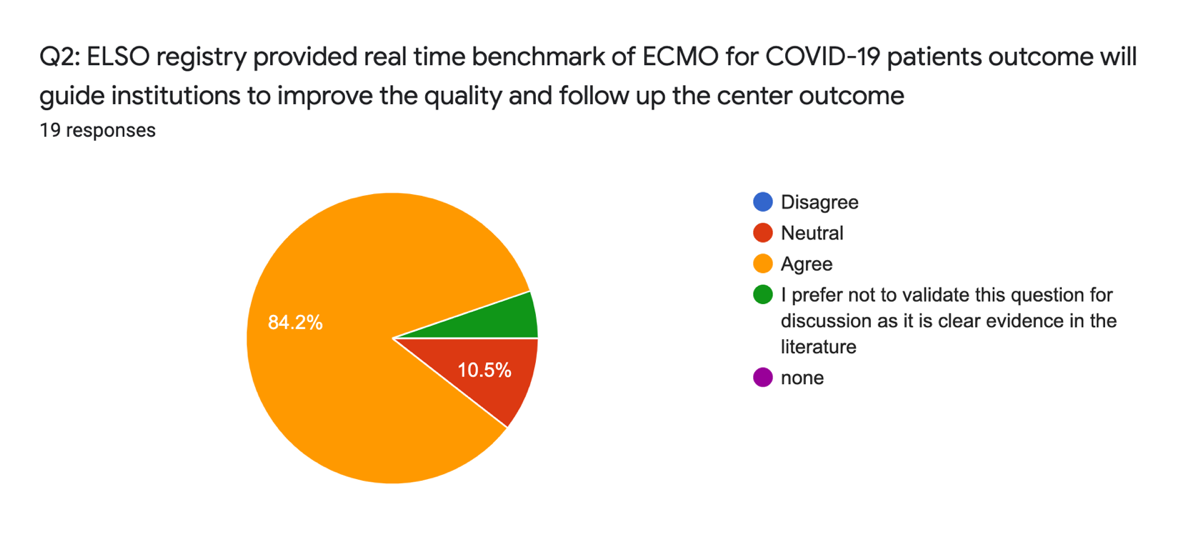


**Supplementary 3**

**Phase IIIa: A summary of the participant comments, discussion, and statement formulation proposals during the first face-to-face meeting.**


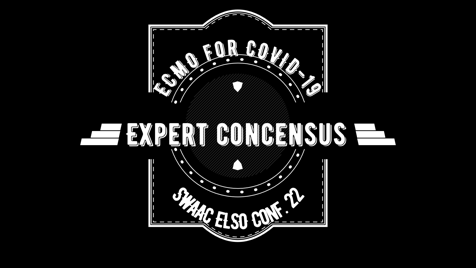


**Phase IIIa expert opinion consensus**

**ECMO for COVID-19**

**Where?**

The meeting was conducted during the SWAAC ELSO conference 2022 in Riyadh, Saudi Arabia.

**How?**

The meeting was face-to-face by invited faculty of ECMO experts who attended the conference in person. Live zoom broadcasting also allowed other faculty to attend and participate in the discussion.

**Who?**

- *Mentor:*

Dr. Mathew Paden & Dr. Mohamed Azzam.

- *Moderator:*

Dr. Ahmed Rabie.

- *Participants in person:*

Dr. Mark Ogino, Dr. Aliaa Abdelfattah, Dr. Yatin Mattha, Dr. Pranay Oza, Dr. Huda Alfoudri, Dr. Alyaa Elhazmi, Dr. Abdulrahman Alfares, Dr. Mohamed Almaan, Dr. Hussam Bahaldeen, Dr. Mostafa Rajab, Dr. Grace van Leeuwen, Dr. Ayed Asiri, Dr. Ali Albashabshi, Mrs. Velia Marta Antonine, Mrs., Monika Tulkas.

- *Participants virtually:*

Alain Combes, Akram Abdelbary, Ahmed Labib, Matthew Paden, Lakshmi Raman, Graeme MacLaren.

**Why?**

The meeting objectives are to discuss the survey results conducted in phase II and come up with recommendations that help institutions and decision makers in countries included in SWAAC-ELSO region put their protocols of ECMO utilization during current pandemic.

**Faculty of the consensus:**

*SWAAC Authors,*

Ahmed Rabie, Akram Abdelbary, Alyaa Elhazmi, Yatin Mattha, Pranay Oza, Huda Alfoudri, Abdulrahman Alfares, Ahmed Labib. Mohamed Azzam.

*Non-SWAAC Authors,*

### Robert Bartlett, Mark Ogino, Alain Combes, Daniel Brodie, Lakshmi Raman, Giles Peek, Ryan Barbaro, Kiran Shekar, Bishoy Zakhary, Graeme MacLaren, Ram Ramanathan

Velia Marta Antonine, Matthew Paden.

*Guest Authors,*

Aliaa Abdelfattah, Mohamed Almaan, Hussam Bahaldeen, Mostafa Rajab،Ayed Asiri, Ali Albashabshi, Grace van Leeuwen, Monika Tulkas.

**The start of the meeting:**

**Mathew Paden:**

Open the meeting with greeting and thank the attendants.

**Domain 1 (D1) Patient selection**


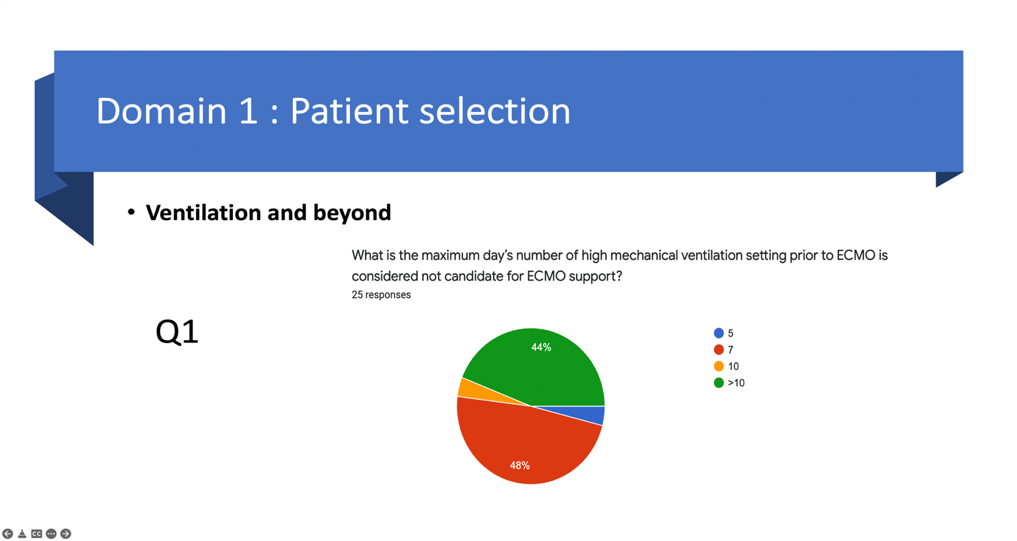
**D1 Q1:**

**Dr. Ahmed Rabie:**

Read the results of the survey analysis, which reflect the opinion of 25 responders of the consensus faculty.

**Dr. Alain Combes:**

I think it's very difficult to answer this question now isolated from the context because obviously, more than ten days may be feasible in young patients without comorbidities, may be not in other patients, so it is very hard to answer those questions for sure isolated from the context and well, I think we should mention that somewhere in the results of that discussion.

**Dr. Mohamed Azzam:**

Thank you, Alain, and all. The success of this conference is because of you and your cooperation, so I really appreciate your being with us. But, again, it is a controversial topic because no evidence guides us to one side or another. I am with the less time for mechanical ventilation to start because of what we saw in Saudi during COVID-19; from our experience, we are retrieving patients from remote hospitals. They did a great job, but we do not have consultants in each hospital. Some of them are internist or specialist level managing these patients, and maybe from my lecture; you notice that we had 700 patients we were following. Maybe 10 or 15% of these patients actually needed ECMO, others need just good management, and they improve, so the longer they are on the ventilator in these places, the more complication we have seen. I asked Mark yesterday if you had seen a lot of surgical emphysema and pneumothorax and things that we saw a lot in Saudi Arabia; I do not have evidence about this. Still, I believe that COVID's common major problem was ventilator management, not well paralyzed, not well sedated high settings on the ventilator. Still, people are not doing the lung-protective strategies or a lung-protective strategy the way it has supposed to be done, so maybe I am biased, but I think having fewer days on the ventilator before initiating ECMO is a good idea, and that also would push people to call us early or to retrieve the patient earlier.

**Dr. Pranay Oza:**

I think there are two things. One is inclusion criteria definitely will prefer to keep the person as early as possible. Now what we are discussing, the exclusion criteria for the person is more than seven days or more than ten days, and then you execute them. Usually, in COVID, what we see is a lot of patients remain on NIV and HFNC before they go on a ventilator; already, they waste a lot of days before we put them on ventilators almost late. So in the pandemic situation where we have a resource limitation, we prefer to put them earlier on ECMO; that is the reason we say more than seven days the chances of survival are going to be less and let the resource be used for better business so you can keep days as an excuse.

**Dr. Yatin Mehta:**

There is no doubt that the earlier you put ECMO, the better it is. We are not discussing inclusion criteria. We are discussing exclusion criteria when you would say you will not put ECMO, so I would say ten days would be more reasonable because, in many of our hospitals in Asian countries, the patients are paying from their own pocket, so that is also a reservation from the family before they say you go ahead or whatever I would think COVID would be a more practical or you can give a 7 to 10 days.

**Dr. Alia Abdelfattah:**

Damage is already there; therefore, not just the days all mechanical ventilation that we have to decide on seven or ten but must be combined with other factors such as age, smoker nonsmoker complications, or patient is infected or not.

**Dr. Graeme MacLaren:**

I was just going to add that I think it is important that people see this not as dichotomous this is not black and white; this is a spectrum, so echoing some of what the other speakers have said, you know, if I am looking after a 65-year-old diabetic hypertensive president who has been on a ventilator for two weeks that I am going to be much more reluctant to offer ECMO more than a 21-year-old who has been on a ventilator for two weeks so again this is not about exclusion or inclusion it is about nuances and saying 07 days I am done, or ten days I am done I think to rob the argument of the appropriate nuance, but we know that the longer the patient has been on the ventilator, the worse they do particularly high-pressure ventilation, but that does not mean we should always say no.

**Dr. Akram Abdelbary:**

Days of ventilation are very important, and it is a very important decision-making factor that we have seen, especially in COVID patients who have peripheral involvement of the lungs that positive pressure ventilation directs to the mainstream of air into the centrally less affected parts of the lungs which increases the possibility of a ventilator-induced lung injury. In these normal parts and hence the delay of recovery, especially in the world where there is no transplantation, I think the important thing is not only invasive mechanical ventilation, because a lot of patients stay on CPAP, for example, 2-3 weeks. I have seen it in our country; they refrain from mechanical ventilation while treating intubating patients because of the bad results of mechanical ventilation, so it depends on the duration and timing of invasive ventilation starting very early in the disease with a short duration has a better outcome.

**Dr. Ahmed Labib:**

So, my concern is that if we stick to our figure like the seven or ten days, we fall into the same trap of septic shock MAP arterial blood pressure of 65. So they have drawn strives for that which has no evidence basis no sound knowledge behind it, and as everyone said, it is a trump, and this is the precise medicine individualized medicine to each patient is a unit which has to be evaluated in the context age, comorbidities, length of stay with the patients coming from. I mean small hospital or a central teaching hospital, all these factors being put together, and I believe if this holistic approach is taken all together, it is much better than sticking to a certain figure, and the way you phrase this in the document could be like Alain said this has to be considered in the context of the patient and or do we recommend not to have a very prolonged mechanical ventilation prior to ECMO however, this can be excluded or can be waived for patients who meet other criteria, so you know you did not break the rules because I am not sure that the seven days rule came from at the moment we are doing their time to ignore initiation as part of the physical care consortium and this is a very interesting study. I am working with a fantastic team, and hopefully, this study will shed some light on this; you know, sort of the issue as well, you think honey.

**Dr. Mathew Paden:**

Continuing to talk about this, but I would say this is going to be something that is you are going to have a very difficult time getting consensus on because I do think it is going to be very patient-specific, and so I might encourage you to move forward a little in this and look at some of these other questions but thank you got some other ones here.

***Conclusion,***

**Dr. Ahmed Rabie:**

Debates to have any decision. We will gather all the comments and then try to figure out three options for one to be chosen again. I will send you a quick survey of which decision you are with, then we will inform you about the result. I think this is the best way to go.

****Recommendation 1***

*While it is clear from the literature and clinical experience that the more days intubated on the ventilator, the worse the outcome and the higher the mortality rate, there is increasing evidence in the literature reporting against this finding. Nevertheless, no certain number of days indicating a poor outcome could be defined in patient selection, there was no consensus amongst panel members, and the votes were equivocal. Therefore, ventilator days should not be used alone as exclusion criteria in discussing patient selection for ECMO. However, it may help in decision-making if concurrently considered with other factors indicating a worse outcome.*

**Scoring system for COVID-19 patient selection (***suggestion raised by Dr. Akram Abdelbary***)**

***Comment***s

**Dr. Akram Abdelbary:**

Can it be used for patient selection apart from the scoring system? Ask for input from Dr. Lakshmi.

**Dr. Lakshmi Raman:**

There is no scoring system for COVID-19 patients. If we have something published, people will likely be looking to that number, which is always a worry. I favor giving a scoring system, but again, are we going to say that we are all the experts and then validate that, or how are we going to come up with what is going to be the other point?

**Dr. Graeme MacLaren:**

A lot of the discussion panels are using scoring systems, and they're mentioning Save and Resp and others. There are interesting studies that are useful in studying patient populations but saying, oh, somebody's got to resp score, and then, I am not going to put them on it. I do not think that is good medicine, and I think we have to be very careful to be dichotomous about this and say, " Oh, look, this is the number, so we are not going to put this patient on ECMO. I do not think that is appropriate. No scoring system, to my knowledge, has ever been available.

**Dr. Alain Combes:**

I agree with what has been said by Graham and Matt basically on the scoring system, so they are very useful to benchmark global activity to adjust for confounders when evaluating the results of different groups and different cohorts but at the patient level. it is reasonable not to take it as a tool to decide; it may help in the decision.

**Dr. Pranay Oza:**

RESP score is used after putting the patient on ECMO; what Yatin means is rather than just putting one recommendation, we can put it in a class where something is recommended, something can be suggested, and something can be against it, so rather than just putting one plus none of the criteria is going to fit into 100%.

***Conclusion,***

**Dr. Ahmed Rabie**

The scoring system suggestion by Dr. Akram as a tool for patient selection of ECMO for COVID-19 was discussed and showed no acceptance from the panel.

***Recommendation 2**

*To date, no scoring system is available to predict survival for severe COVID-19 ARDS patients who require ECMO. The available scoring systems for non-COVID-19 should not be considered a tool that could be used at the bedside; it may help but not guide decision-making.*

**D1 Q2,3**


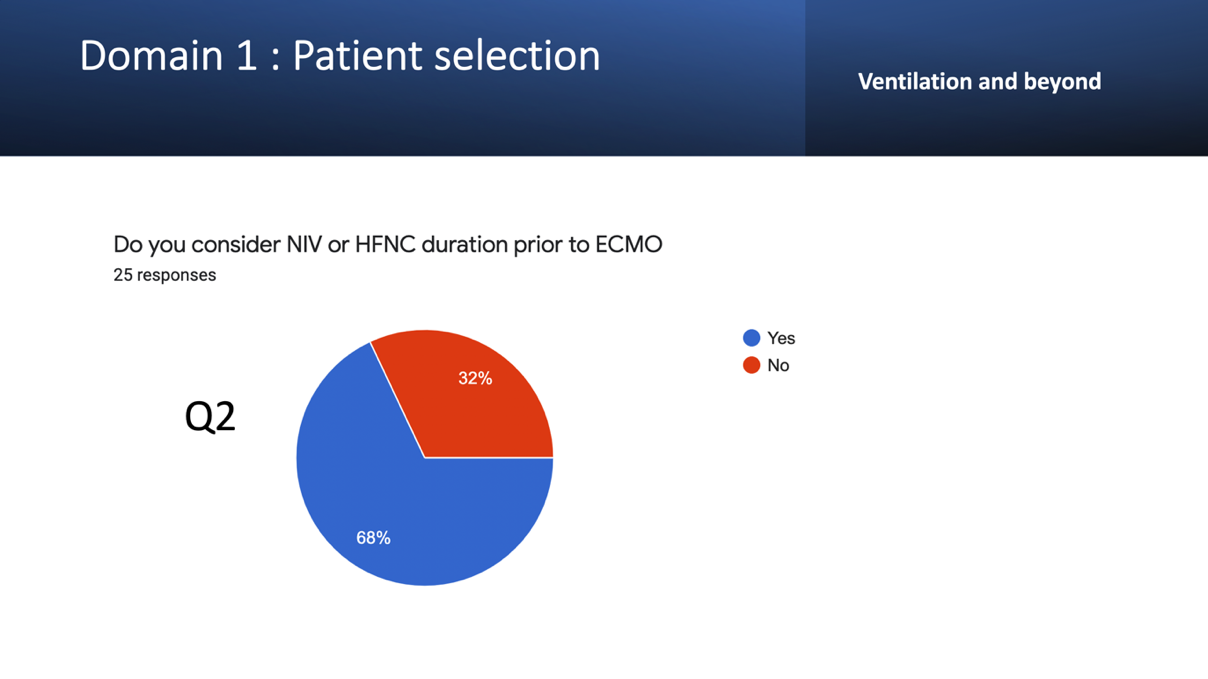


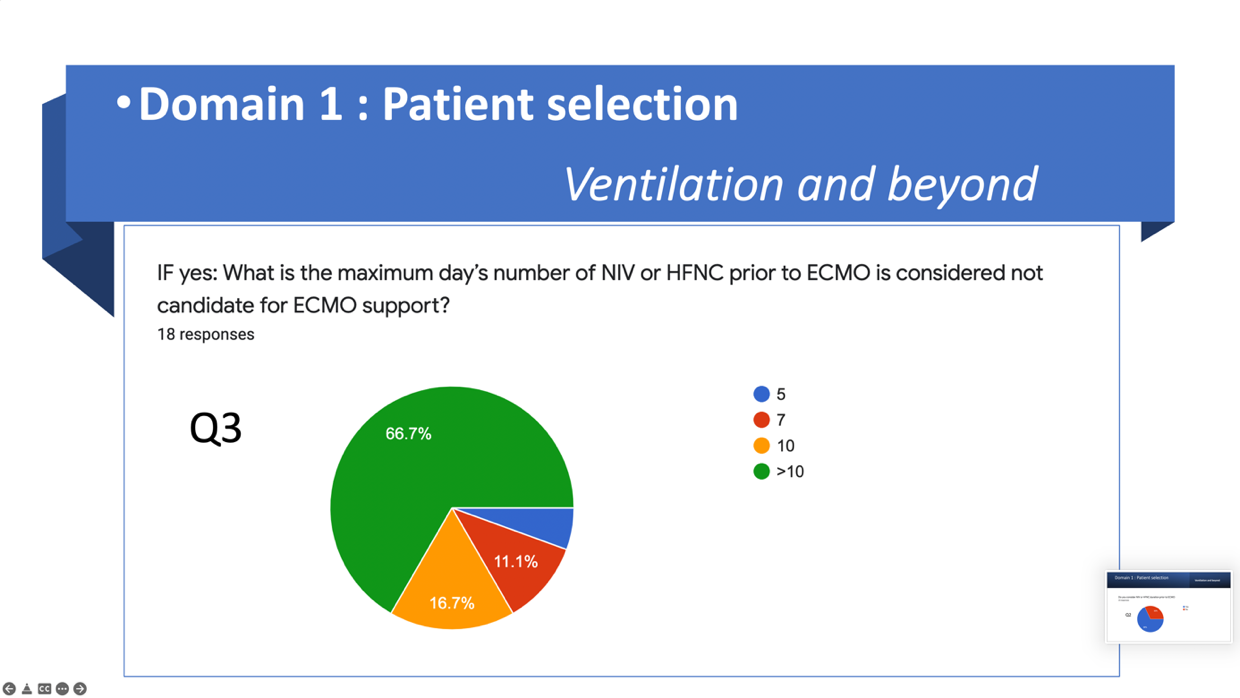


***Comments,***

**Dr Abdulrahman Alfares:**

We are trying to mitigate ventilator-induced lung injury, and we know mechanical ventilation can contribute to that. I think it would be difficult if you say you would give maximum days, especially for COVID patients where there is wide use across the world of the high-flow nasal cannula and in those patients, so I am not sure that it would be so wise to combine these two modalities of NIV as well it depends how you use it whether you use a mask helmet or whatever interface and each has pluses and minuses in hypoxemic respiratory failure so, I think this is even more controversial.

***Conclusions,***

*68 % for considering pre-intubation noninvasive support is fair to be accepted, and it is better to keep further discussion for more controversial questions. However, it is worth differentiating between noninvasive mechanical ventilation and HFNC in discussing this concern or future research investigations.*

****Recommendation 3***

*The panel agrees that the number of days on NIMV or HFNC may be related to the patient outcome on ECMO; however, no evidence is available to date supporting its use in ECMO patient selection. Therefore, it should not be considered individually in discussing COVID-19 patient selection for ECMO at the meantime. Instead, we recommend that future study designs discriminate between NIMV and HFNC and conduct more prospective or observational studies to answer this critical question.*

**D1Q4**

**
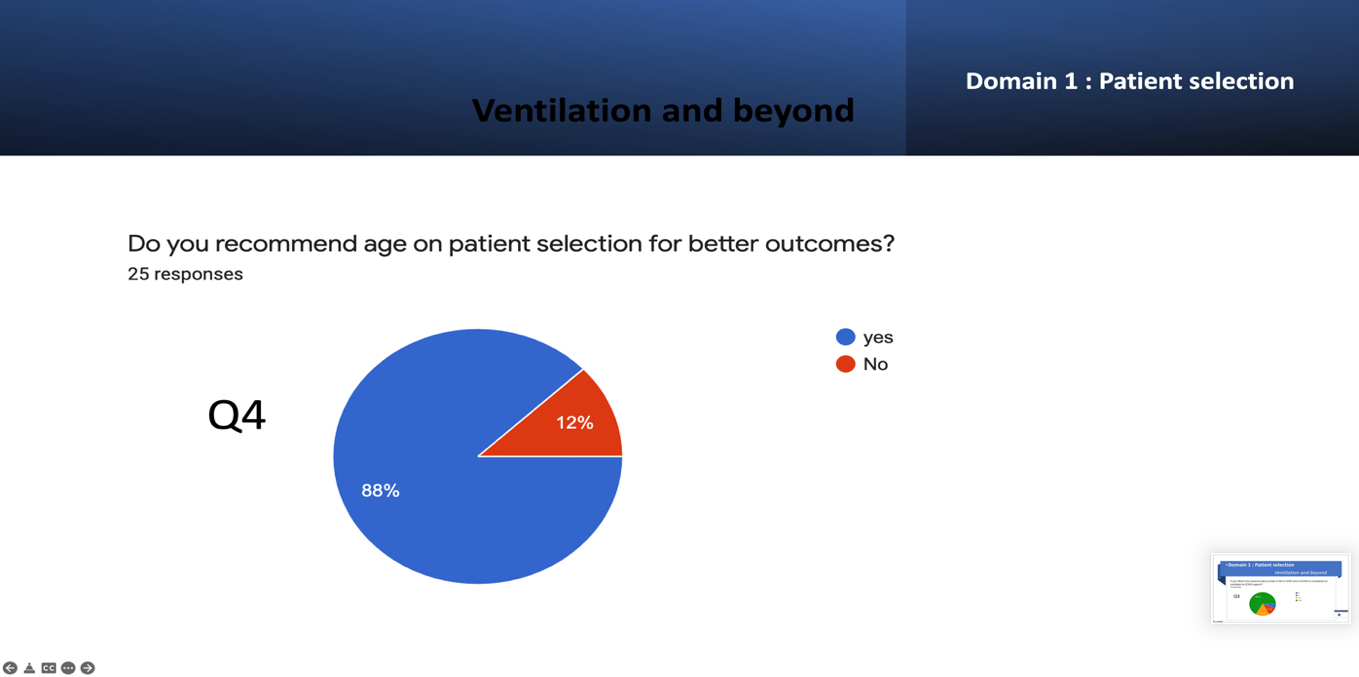
**

***Comments,*** No comments

***Conclusion,***

All accepted the survey result that age could guide patient selection for a better outcome.

****Recommendation 4 Age*** *has a relation to patient outcomes and may be considered during patient selection for a better outcome.*

**D1Q5,6,7**


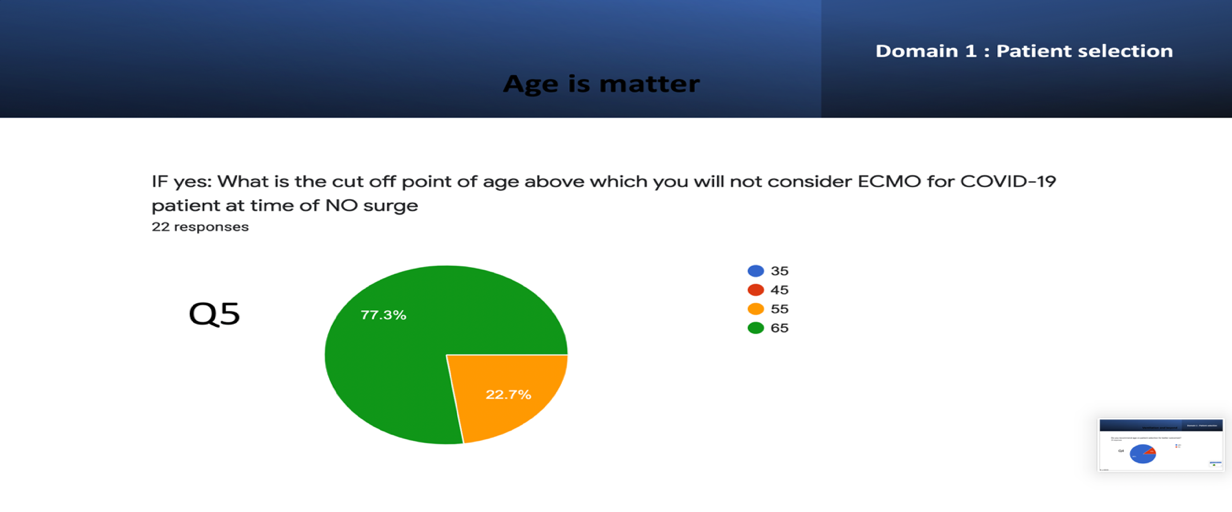


**
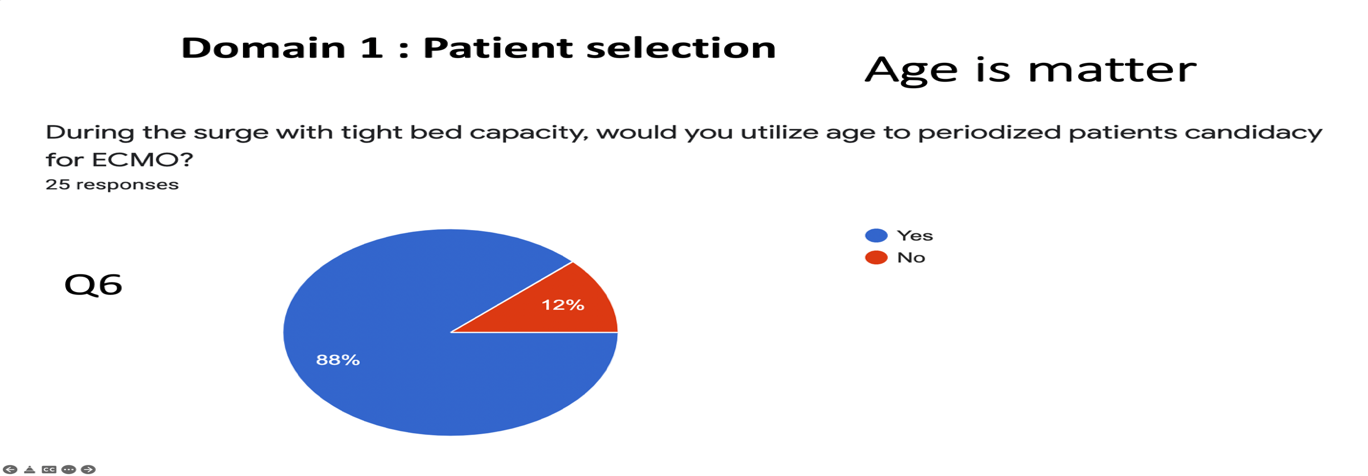
**

***
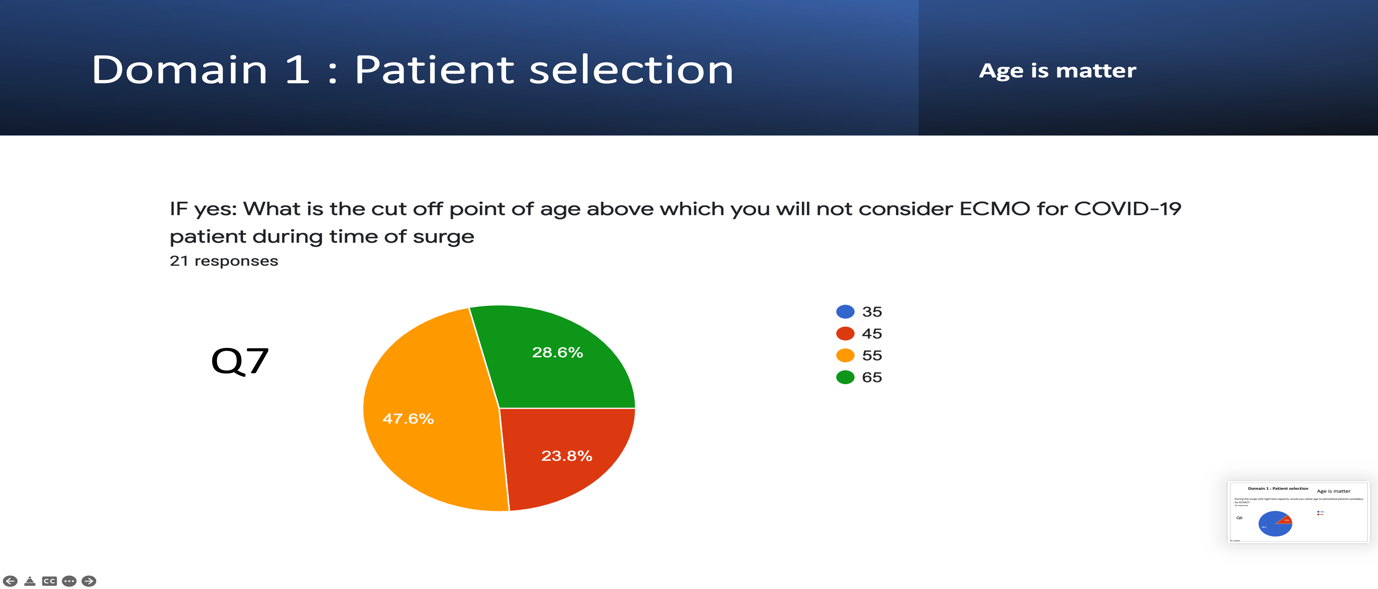
***

***Comments,***

**Dr Abdulrahman Alfares:**

Data for the past two years tells us and this is doctor Combes very elegantly demonstrated in his work as well and from Paris, that younger people do better, so I think it won't be wise that we say we would do ten years older, as SAWAAC-ELSO organization I'm not sure that this would be a wise statement that we would have the evidence that younger people do better, so I think that question maybe has to be revisited or revised because I disagree, to be honest with 77%.

**Dr. Yatin Mehta:**

If you differentiate between Surge and non-surge, I think it does make sense to have a cutoff of 55 years, so if you circulated that paper to this group, the number percentage would go up. So, who would agree with 55? So, if you're taking 65, I would agree that maybe it should be lower, but if it is 65 in no surge, it has to be lower for Surge, and 55 does make sense.

**Dr. Huda Alfoudri:**

I think both age and number of days on the ventilator cannot be judged individually. So if we can combine them together in a score which we have, like the Resp score, then at least this can guide us rather than just the individual number because we have seen patients, you know, with older age groups who are very fit and 60 or 65 years old. We've seen patients who did not survive in their 30s, but they came from whatever other factors, so it's difficult to judge by the number.

**Dr. Ahmed Rabie:**

This is actually what we will try to write and deliver every question we have answered is not to be used individually for proper patient selection; it should be combined information about the patient that will guide proper patient selection.

Conclusion:

I think we will go with 55 years as a cutoff point during the time of Surge and overwhelmed healthcare system.

****Recommendation5***

*Age may be used to prioritize patient support on ECMO due to constraints of resources and overwhelm of the health care system. Therefore, the consensus amongst the panel was to accept 55 years as a cutoff point for age during this time of surge and 65 years for no-surge time.*

**D8Q1**

**
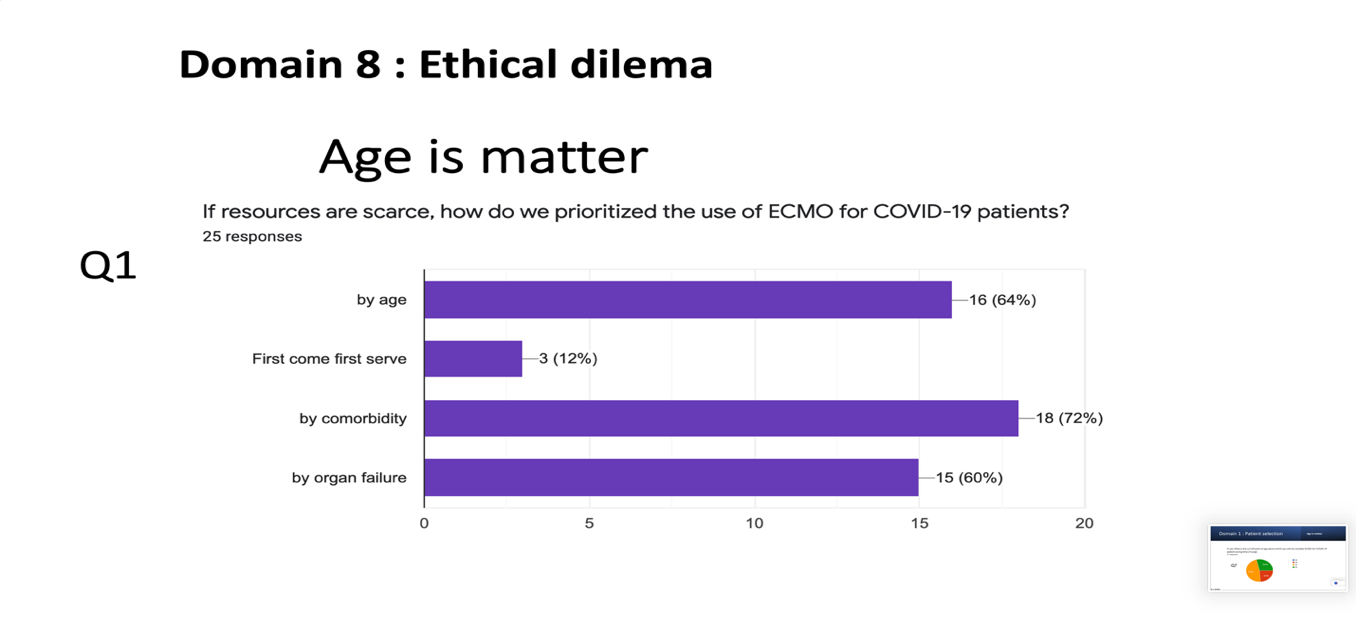
**

***Comments,***

**Dr. Ahmed Rabie**

Because age is very controversial, we bring a question from the domain of ethical dilemma…. we discuss age matters,

**Dr. Yatin Mehta:**

Yeah, it could always make sense because of resource constraints there. You have to consider both this and also when you are taking exams or recommendations or guidelines; the way to go is that you either suggest that means there's enough evidence for that or to suggest which is a milder recommendation or we suggest against if something is not to be done.

**Dr. Alain Combes:**

If you are facing an ethical dilemma because you have a scarce resource, then clearly you have to prioritize the patient by age, comorbidities, and organ failure; it's an interplay between all these three factors, and for example, patients with severe immunocompromised status clearly will not qualify in that situation because we know mortality is usually at least 70% in regular talks it may be even higher in that in that time so they clearly should not be provided at most support in this type of condition.

**Dr. Ahmed Rabie:**

The immunocompromised patient is considered comorbidities. So, that means you choose comorbidities to start with for periodization rather than age and organ failure

**Dr. Alain Combes:**

It's difficult to say by which criteria you will start, BUT what you might do here is to take the three or four cases vignettes showing different cases and explain why you chose one patient and not the others; clearly, we know that age has a major impact and age over 55 or 60 clearly in COVID-19 patients tested with much higher mortality, same for organ failure and same from commodities which are the most important. It's very difficult to answer that question

**Dr. Ahmed Rabie:**

If you are on call and the bedside doctor calls you to say that he has two patients in need of ECMO and gives you the criteria of both patients now, you have to decide as you have only one membrane. What would be your decision?

**Dr. Alain Combes:**

Yeah, this might be a solution to deal with that type of question

**Dr. Alia Abdelfattah:**

The suggestions come from our observation of our experience in the previous two years; we have to choose what we could extrapolate from our experience during these last two years. Comorbidities are very important and they combined with age, think it's very important

**Conclusion**

**Dr. Ahmed Rabie:**

OK, we all agreed that surveying age, comorbidities and organ failure to prioritize patients is scarce resources. We don't have a score and should have some advice for at least the SWAAC region to guide institutions to put their protocol and policy.

****Recommendation 6***

The ethical dilemma on the patient selection that arose due to scarce resources is a challenge; age, comorbidities, and organ failure may be used to prioritize patients with the interplay between these three factors to choose which factor used to start patient selection, for example, 57 years old healthy may be a good candidate than 25 years old immunocompromised patient with multiorgan failure while the same patient is not a good candidate for a 25-year-old diabetic patient. In the first case, prioritization starts with comorbidities first, while in the second case, it starts with age first.

**D1Q8**


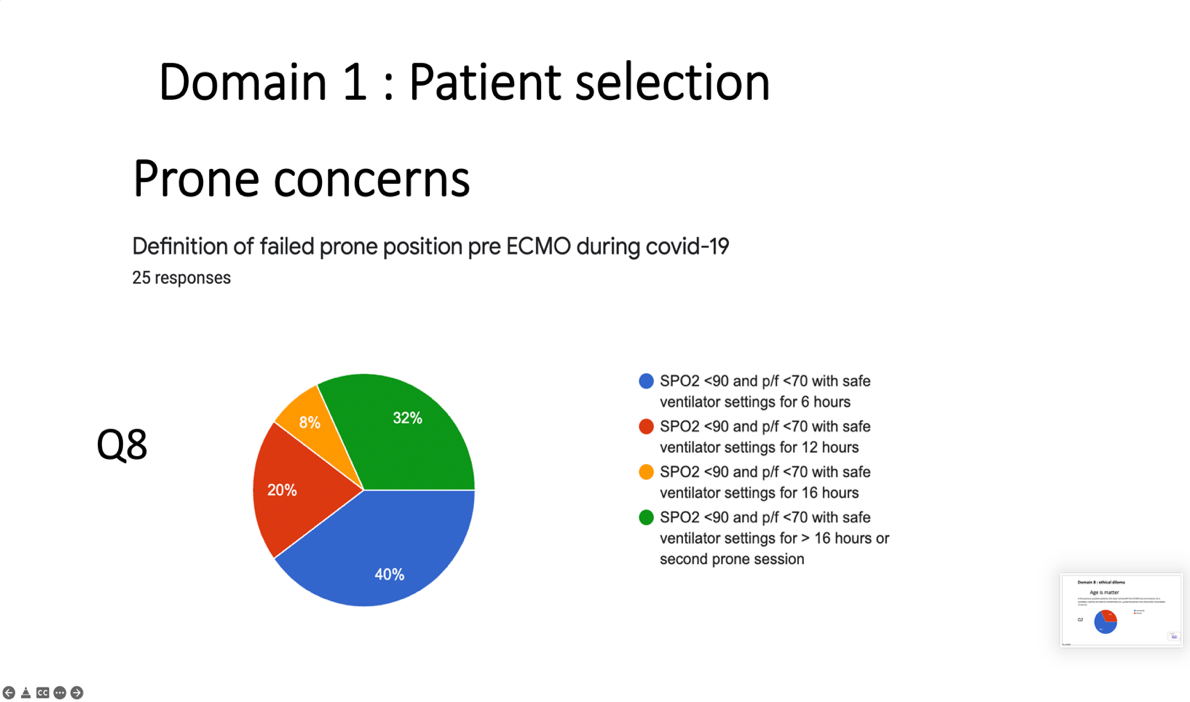


***Comments,***

In the definition of failed prone position therapy, I think we did not perform a good question for the other because we said that saturation below 90 and be a friendship below 70; we served in the setting for six hours, almost like the earlier criteria and saturation less than 90 for less than 12. 16 hours or more than 16 hours, we find that people put patients for 24 hours’ So what should we use for this to decide the patient? I think it's also controversy here 32 with more than 16 or other sessions 40 was with and it 20 with 12 and it was 16

**Dr Abdulrahman Alfares:**

So right, this is the problem with having strict numbers for decision-making. Maybe we should have the same criteria as we had regularly similar to the EOLIA criteria hours PO2 hypoxemia or hypercapnia despite the patient supine or prone, it should be the same and even when you go more towards prone positioning and failure of proning the definition for that is not clear or concise if you want to go back to the trial itself they had a specific definition for failure so I think we should stick with the known criteria that we choose for initiating which is already clear from the consensus guidelines elsewhere.

**Dr. Alain Combes:**

Well, it was shown that the improvement in the PF ratio during prone positioning was not predictive of mortality based on the results of the ~~PROCIVA~~ PROSIVA trial, the large trial which showed that there was a huge benefit of positioning a patient with prone. So, I guess it's difficult here to say what is a failure-prone because it's not because the patient is going to improve the P/F that we can say that it's actually a patient will have a success of prone position now unless the PF goes above 200 and there was massive recruitment, and this is the first issue herein. The second I do believe, as I mentioned before, that we should stick with the criteria we have right now is the earlier criteria and mentioned that the best results, at least in EOLIA, were for patients with the PF above the threshold, which was 66. So, well, I think we need to be printing here to stick with the EOLIA criteria and say that it's very difficult to quantify what clearly failed a prone positioning session.

**Dr. Mohamed Almaan:**

OK, in fact, what's more important is we do proneing mainly to recruit the lung, and what's more important is the CO2 level rather than the P/F ratio by itself and most importantly, we looked at the people who did crossover in the earlier they had a higher airway pressure so, I think it's not appropriate to make a decision upon P/F alone it's probably better to consider airway pressure and the CO2 clearance.

**Dr. Mohamed Azzam:**

In the beginning, when we put the criteria in Saudi Arabia, we were not sure whether ECMO patients were going to benefit or not, and we were afraid of the crisis that would happen in case if we opened doing ECMO liberally like before COVID. So, end of the criteria, the prone position was part of the selection criteria, so did you put the patient in a prone? Did you go through the steps of the management of ARDS. as I said, there is a periphery hospital that will call us sometimes on a patient who needs ECMO and still they did not go through the process. so it was part of putting people on the process so, they can manage the problem.

**Dr. Ahmed Rabie:**

We didn't reach an agreement about this, so we will hang this question until we discuss it again through grafting something, and we will see if people work.

***Conclusion:***

Discard this question

**D1Q10**

**
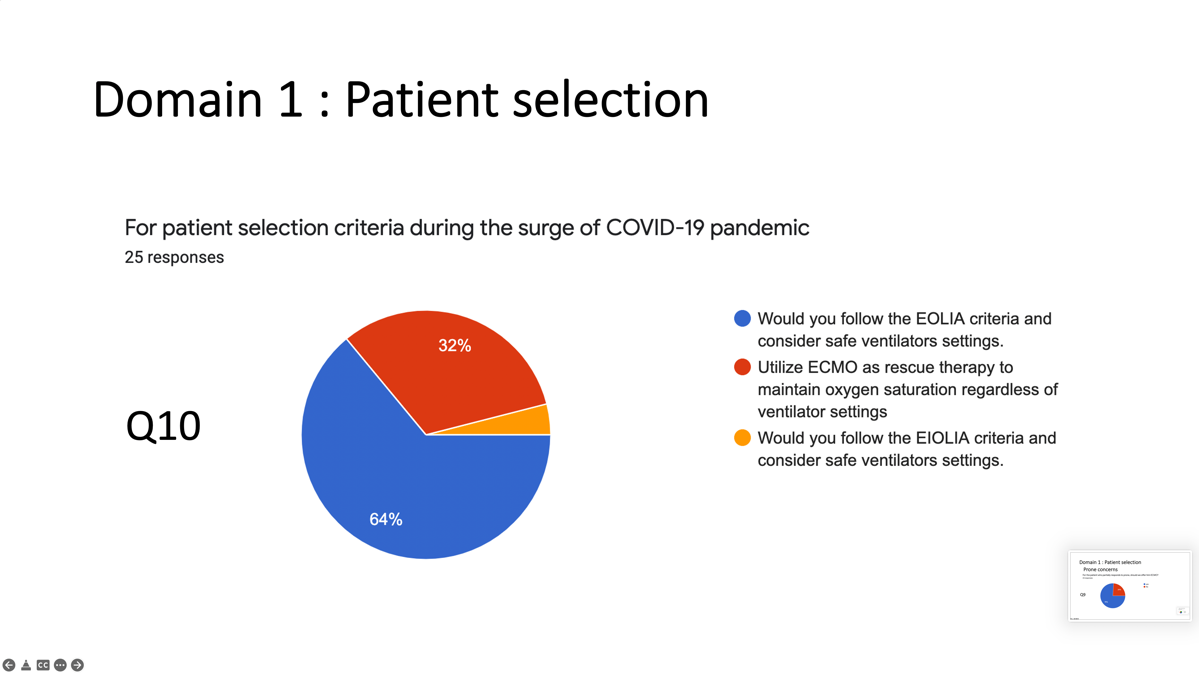
**

***Comments,***

**Dr. Ahmed Rabie:**

Here in front of you, there is a question for a patient selection criterion during the surge time of the COVID-19 pandemic, would you follow EOLIA criteria or as a rescue therapy to maintain oxygenation? 64 % for EOLIA and 32% only to rescue. So, did you mind putting this as a final agreement? In our practice during the pandemic, we have to use a prone rescue therapy only to avoid ECMO due to constraints of resources. This eventually leads to a poor outcome on ECMO, and I think this is also one of the factors that lead to poor results.

***Conclusion***,

All the panel agreed with the survey result to stick with EOLIA criteria and not to utilize ECMO as a rescue therapy to maintain oxygenation for better outcomes and allocation of resources.

******Recommendation 7***

*The insufficient practical experience of utilizing ECMO as rescue therapy for cases who cannot maintain oxygenation regardless of the ventilator setting due to resource constraints is inappropriate; it may be related to increased mortality in regions using this practice. Therefore, the panel recommends strict compliance with EOLIA criteria in ECMO patient selection and no need for deviations from the regular practice prior to COVID-19.*

**D1Q9**

**
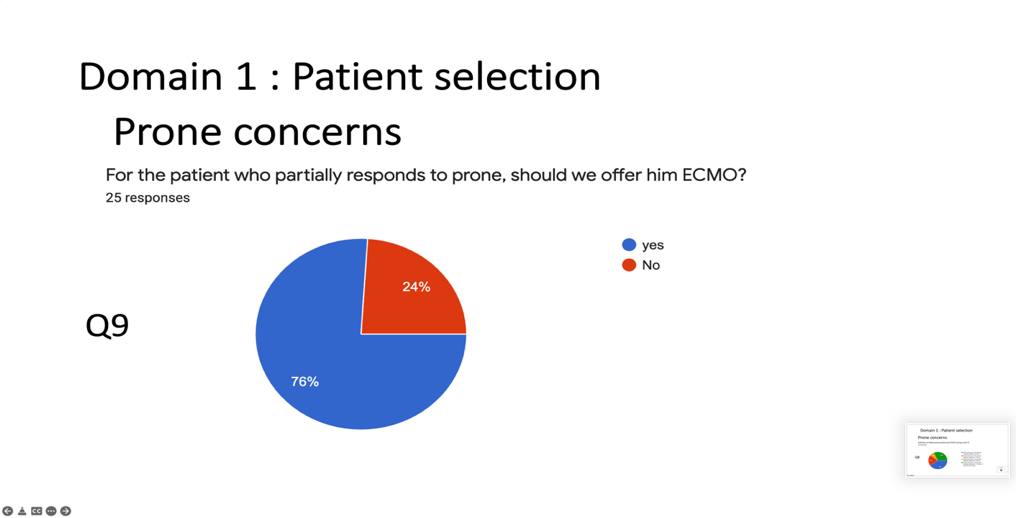
**

***Comments,***

**Dr. Ahmed Rabie:**

We have a kind of patients who partially responds to prone and worsening again when returned on supine position so those kind of people we used to try and try and try because we don't want to utilize our resources then eventually we find those patients became in a terrible shape with prolonged ventilation and the prolonged prone became not candidate for ECMO so we're asking here, and we agree that the partial responders to prone showed offered ECMO, survey result reached 76%.

****Recommendation 8***

*Patients commonly seen during the current COVID-19 pandemic who initially respond to prone and deteriorate on turning supine are considered partial prone position responders. Primarily, they did not improve with repeated prone positions, and the panel recommends offering them ECMO earlier. Future research to answer this question is recommended.*

**End of the meeting:**

**Dr. Mathew Paden**

We just had one thing to finish this, which is, that I think it is very important that you all are doing what you're doing, trying to come up with consensus definitions and consensus ways to manage. The other thing that I would remind you is as we talked about earlier Alain and his colleagues were able to do something very similar to this within Paris itself our Chilean colleagues around the world have been able to do it, use those documents in the things that the other folks have put together is there a method to take and adapt that for what is correct for the SWAAC-ELSO region but I think those are our excellent starting points and you have done a fantastic amount of work here, and I look forward to see how this progresses thank you for a very interesting session!

***Dr. Ahmed Rabie:***

Thank you, thank you all, thank you, we will terminate this phase, summarize, and will send you all to sit for another phase; most probably, it will not be face to face unless if we have any new events, if not we will make it as a zoom conference agree? OK!

(*) proposed recommendation not discussed during the meeting.

**Supplementary 4**

**Phase IIIb: Summary of the second in-person meeting's comments, discussions, and statement formulation suggestions.**


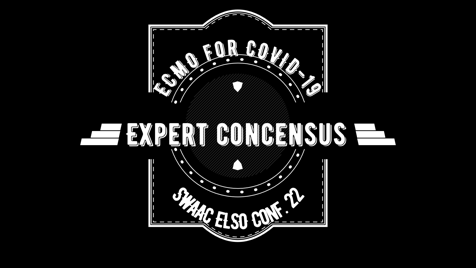


**Phase IIIb expert opinion consensus**

**ECMO for COVID-19**

**Where?**

The meeting was conducted during 16 April 2022 in Riyadh, Saudi Arabia.

**How?**

With hybrid face to face and virtually

**Who?**

- *Mentor:*

Dr. Mohamed Azzam.

- *Moderator:*

Dr. Ahmed Rabie.

- *Participants in person:*

Dr. Alyaa Elhazmi, Dr. Mostafa Rajab, Dr. Ayed Asiri, Dr. Mohamed Azzam, Dr. Ryan Abdullah, Dr. Mohamed Foteih

- *Participants virtually:*

Dr. Pranay Oza, Dr. Huda Alfoudri, Ahmed Labib, Velia Marta Antonine, Mrs., Monika Tulkas, Dr. Abdulrahman Alfares Dr. Giles Peek

**Why?**

The meeting objectives are to discuss the survey results conducted in phase II and come up with recommendations that help institutions and decision makers in countries included in SWAAC-ELSO region put their protocols of ECMO utilization during current pandemic.

**Faculty of the consensus:**

*SWAAC Authors,*

Ahmed Rabie, Akram Abdelbary, Alyaa Elhazmi, Yatin Mattha, Pranay Oza, Huda Alfoudri, Abdulrahman Alfares, Ahmed Labib. Mohamed Azzam.

*Non-SWAAC Authors,*

### Robert Bartlett, Mark Ogino, Alain Combes, Daniel Brodie, Lakshmi Raman, Giles Peek, Ryan Barbaro, Kiran Shekar, Bishoy Zakhary, Graeme MacLaren, Ram Ramanathan

Velia Marta Antonine, Matthew Paden.

*Guest Authors,*

Aliaa Abdelfattah, Mohamed Almaan, Hussam Bahaldeen, Mostafa Rajab،Ayed Asiri, Ali Albashabshi, Grace van Leeuwen, Monika Tulkas, Dr. Ryan Abdullah, Dr. Mohamed Foteih

**The start of the meeting:**

**Mohamed Azzam:**

Open the meeting with greeting and thank the attendants.

D2, Q1.

**Dr. Ahmed Rabie:**

Read the results of the survey analysis, which reflect the opinion of 25 responders of the consensus faculty.

**Comments:**

After discussing the results of the questions related to anticoagulants utilization during COVID-19 the panel agree that the questions are not comprehensive to this topic

***Conclusion,***

The questions results are not accepted by the panel and decision is to discard it and preparing a statement in this topic to be discussed next meeting

***D2Q3***

***Conclusions,***

*68 % for considering pre intubation noninvasive support is fair to be accepted, and it is better to keep further discussion for more controversial questions. However, it is worth differentiating between noninvasive mechanical ventilation and HFNC in discussing this concern or future research investigations.*

****Recommendation 3***

*The panel agrees that the number of days on NIMV or HFNC may be related to the patient outcome on ECMO; however, no evidence is available to date supporting its use in ECMO patient selection, therefore it should not be considered individually in discussing COVID-19 patient selection for ECMO at the meantime. Instead, we recommend future prospective or observation studies to answer this critical question and differentiate between NIMV and HFNC in designing future research.*

**D1Q4**

**
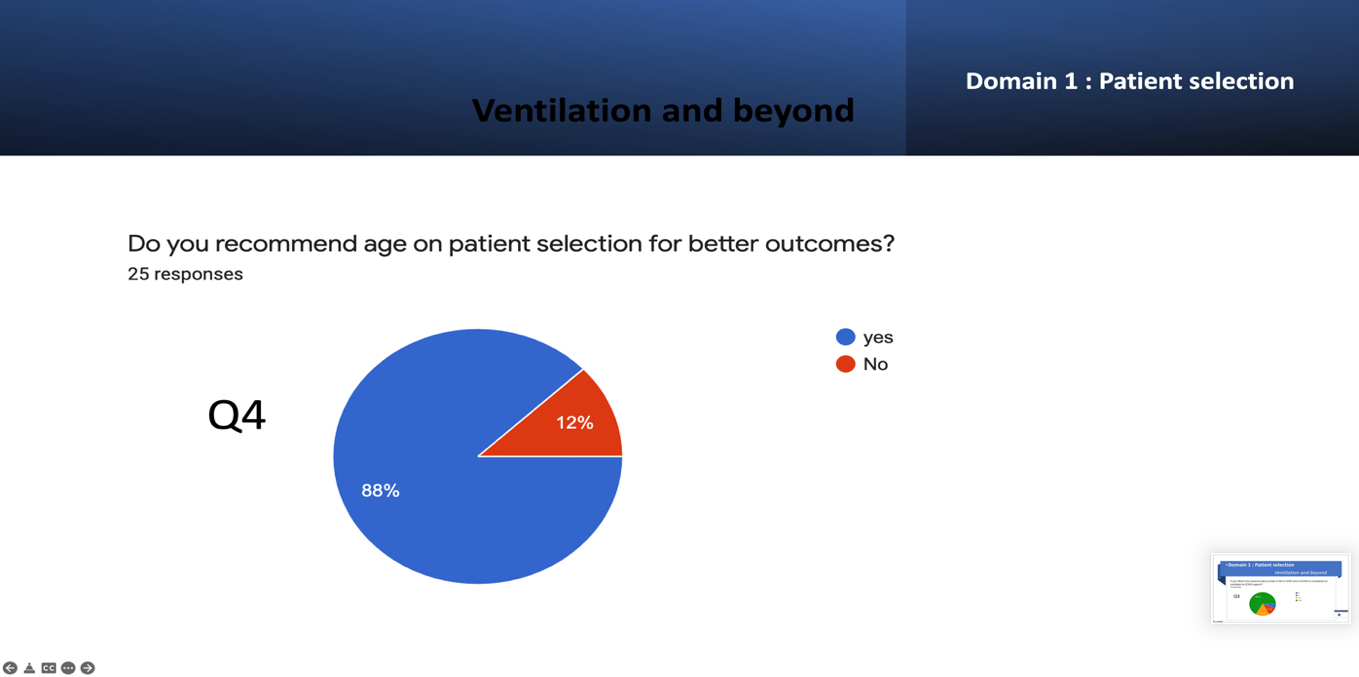
**

***Conclusion,***

All accepted the survey result that age could guide patient selection for a better outcome.

****Recommendation 4***

*Age has relation to patient’s outcomes and may be considered during patient selection for a better outcome.*

**D1Q5,6,7**


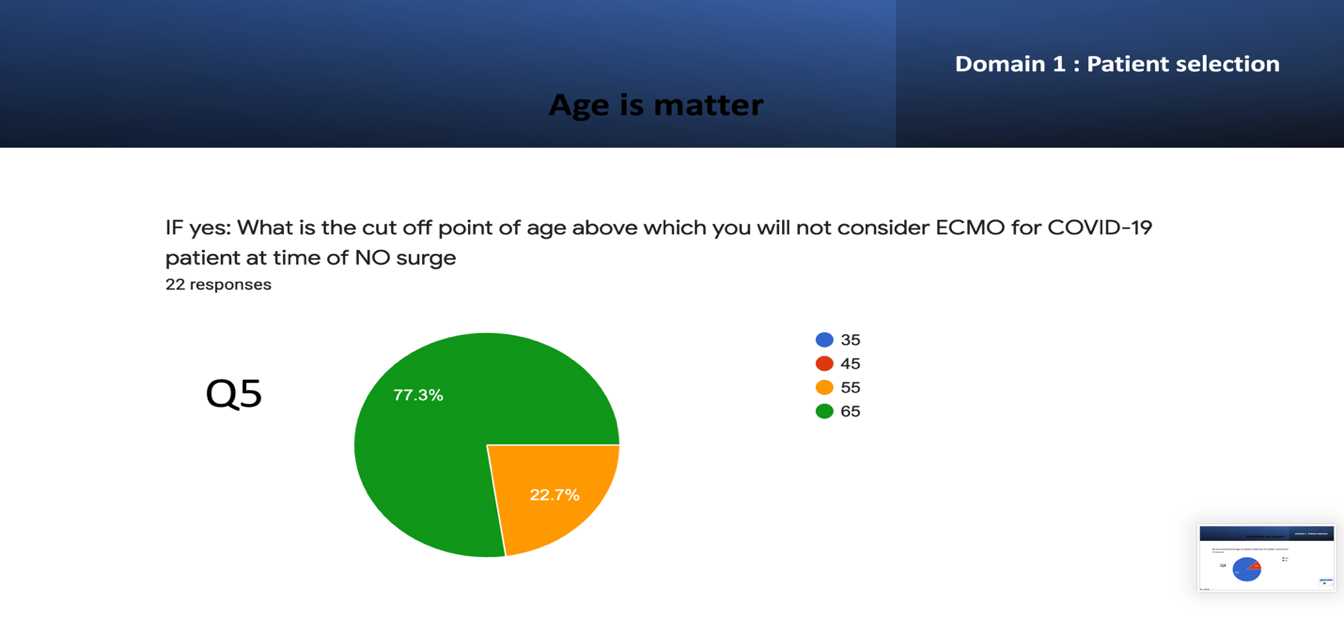


**
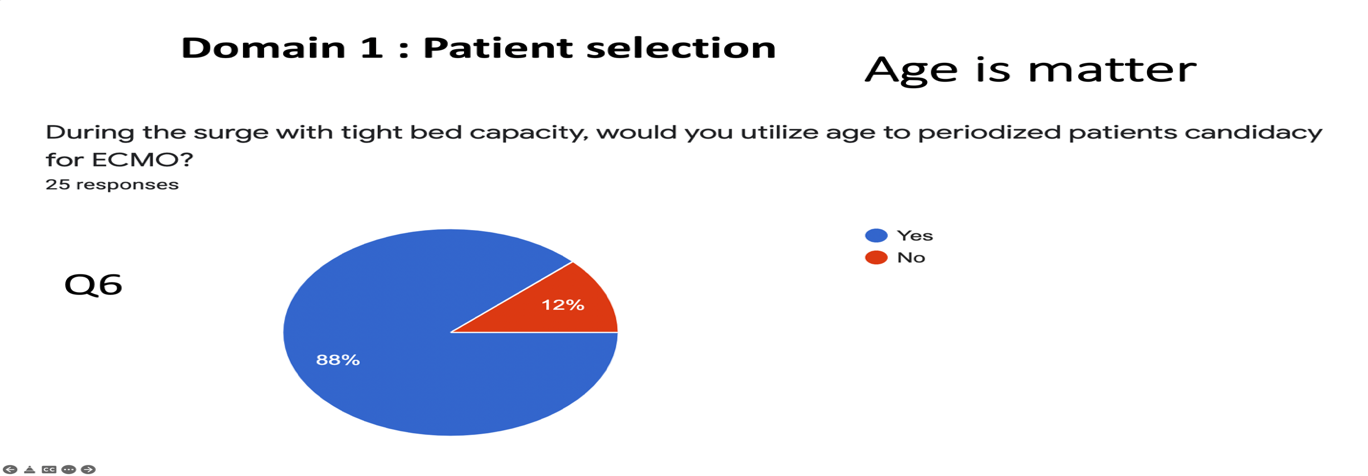
**

***
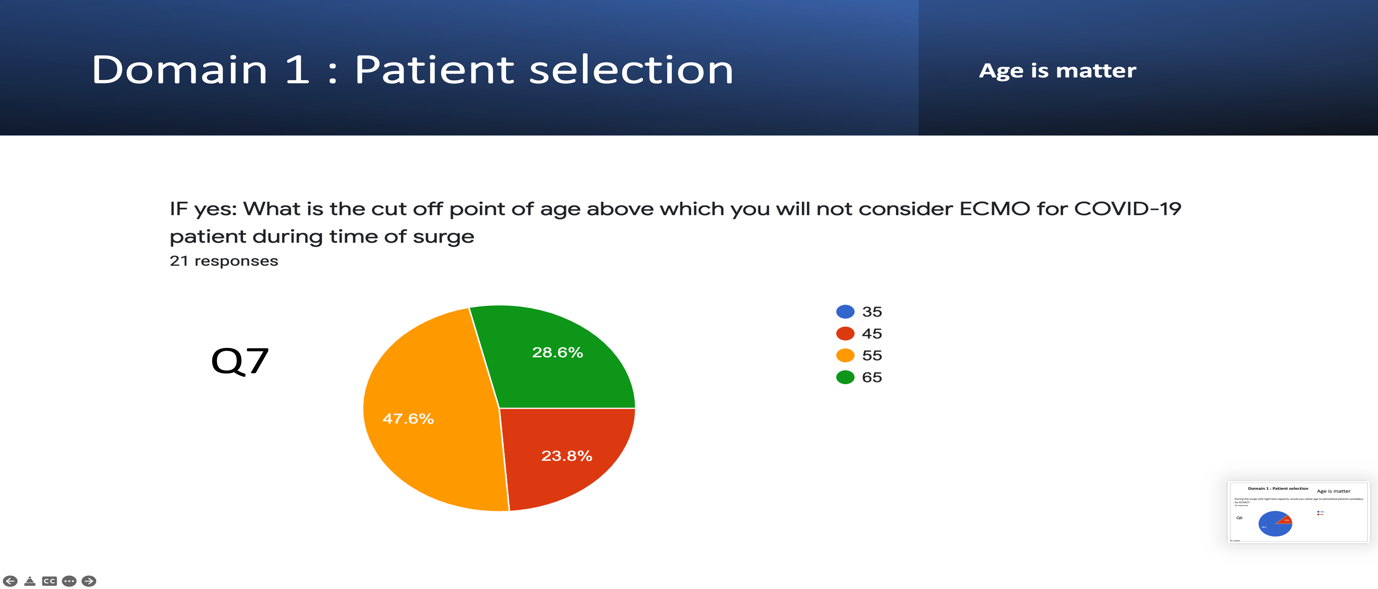
***

So, this is actually what we will try to write and deliver that every question we have answered is not to be used individually for proper patient selection; it should be combined information about the patient that will guide proper patient selection.

Conclusion:

I think we will go with 55 years as a cutoff point during the time of Surge and overwhelmed health care system

****Recommendation5***

*Age may be used as a factor to prioritize patient support on ECMO due to constraints of resources and overwhelm of the health care system. Therefore, the consensus amongst the panel was to accept 55 years as a cutoff point for age during this time of surge and 65 years for no-surge time.*

**D8Q1**

**
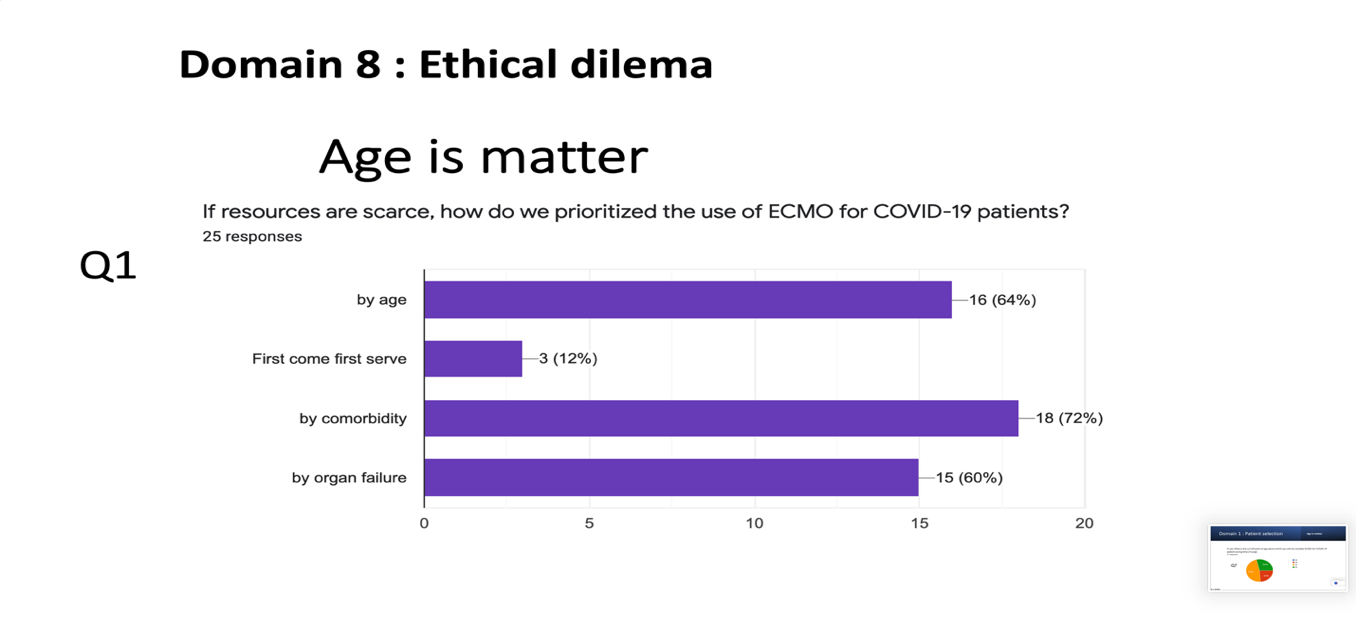
**

**Dr. Ahmed Rabie:**

OK we are all agreed that surveying age, comorbidities and the organ failure to prioritize patient if scarce resources. We don't have a score and we should have some advice for at least SWAAC region to guide institutions to put their protocol and policy.

****Recommendation 6***

Ethical dilemma on the patient selection that raised due to scarce resources is challenging for decision-makers; surveying age, comorbidities, and organ failure may be used to prioritize patients with the interplay between these three factors to choose which factor used to start with on patient selection, for example, 57 years old healthy may be a good candidate than 25 years old immunocompromised patient with multiorgan failure while the same patient is not a good candidate if compared to 25 years diabetic adult, the first case prioritization starts with comorbidities first while the second case starts with age first.

**D1Q8**


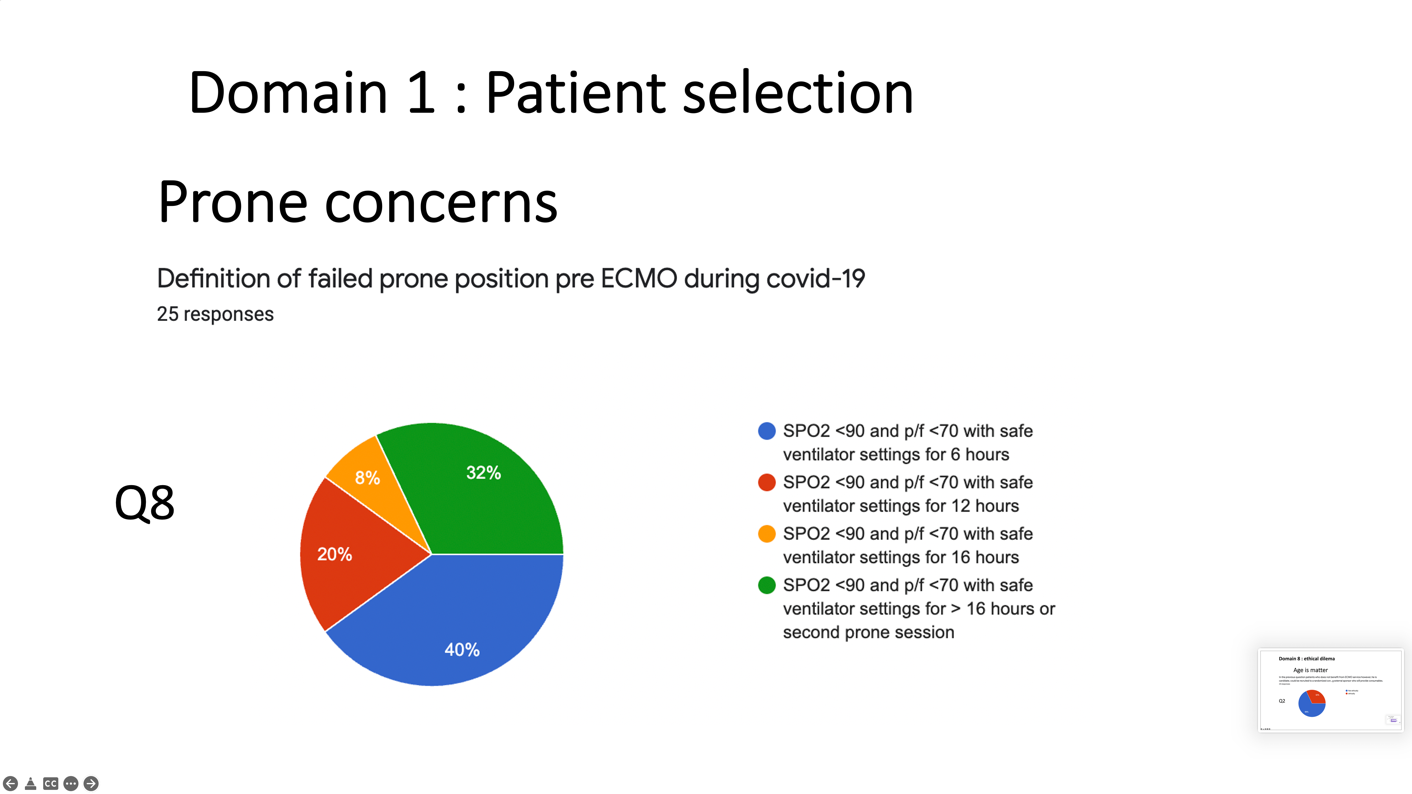


**D1Q10**

**
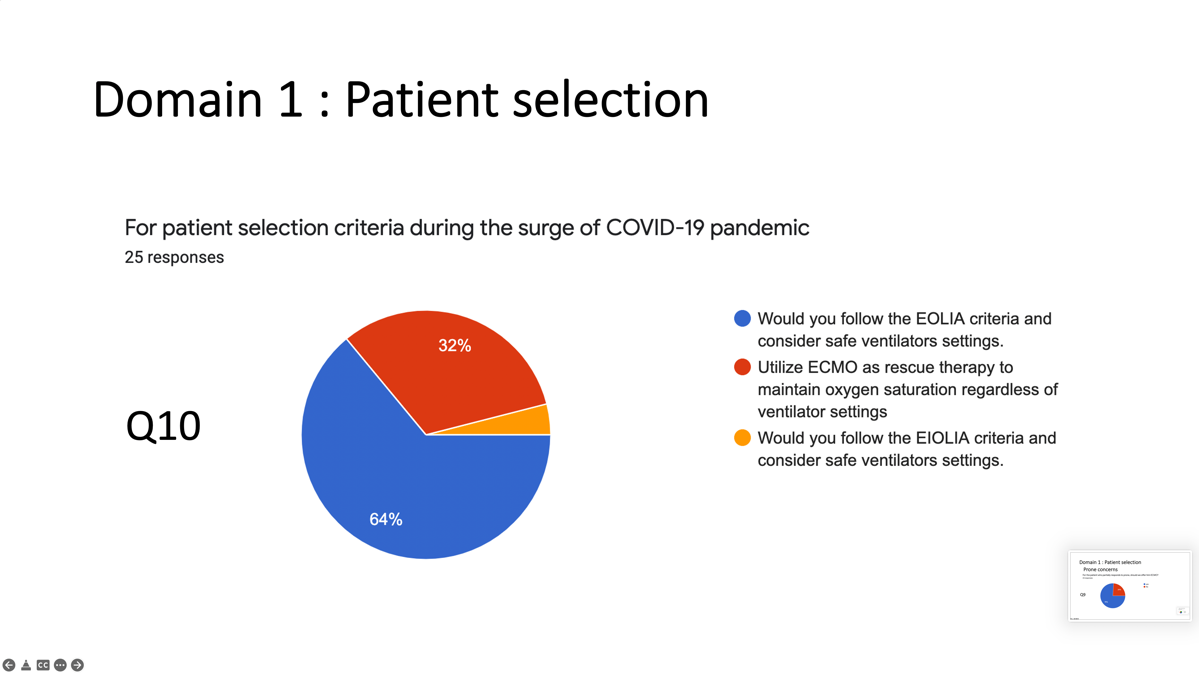
**

***Conclusion***,

All the panel agreed with the survey result to stick with EOLIA criteria and not to utilize ECMO as a rescue therapy to maintain oxygenation for better outcome and allocation of resource

******Recommendation 7***

*The insufficient practical experience of utilizing ECMO as rescue therapy for cases who cannot maintain oxygenation regardless of the ventilator setting due to resources constraints is inappropriate; it may be related to increased mortality in regions using this practice. Therefore, the panel recommends strict compliance with EOLIA criteria in ECMO patient selection and no need for deviations from the regular practice prior to COVID-19.*

**D1Q9**

**
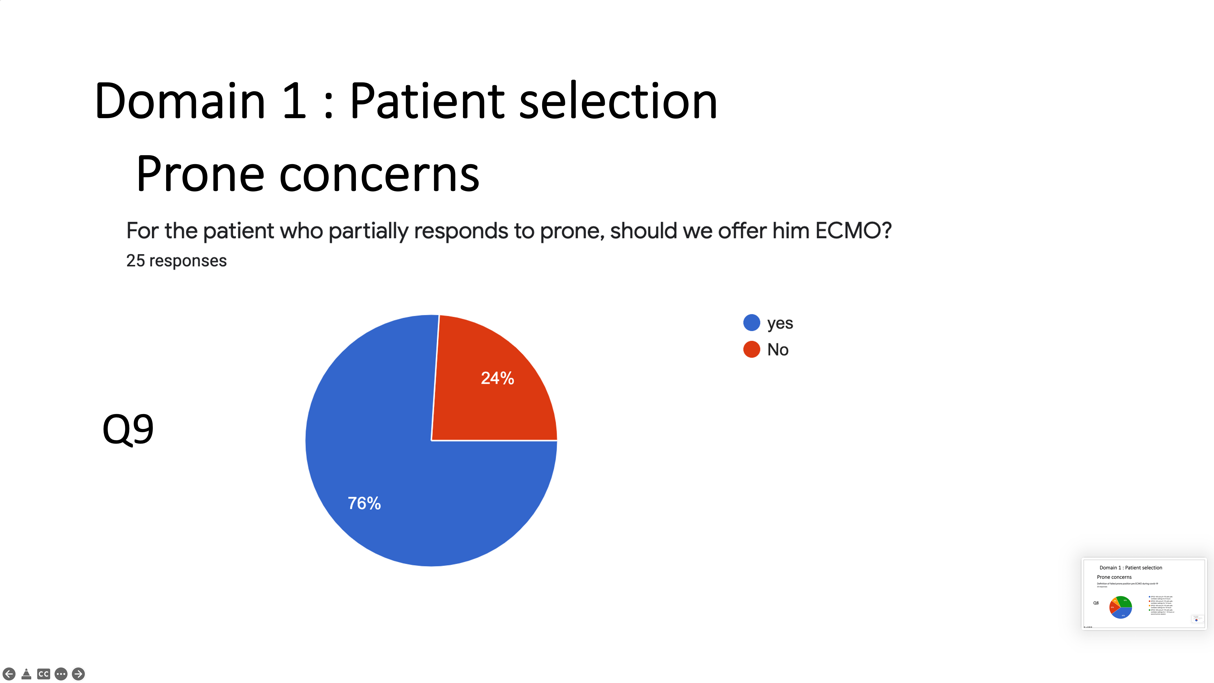
**

***Comments,***

**Dr. Ahmed Rabie:**

We have a kind of patients who partially responds to prone and worsening again when returned on supine position so those kind of people we used to try and try and try because we don't want to utilize our resources then eventually we find those patients became in a terrible shape with prolonged ventilation and the prolonged prone became not candidate for ECMO so we're asking here, and we agree that the partial responders to prone showed offered ECMO, survey result reached 76%.

****Recommendation 8***

*Patients commonly seen during the current COVID-19 pandemic who initially respond to prone and deteriorate on turning supine are considered partial prone position responders. Primarily, they did not improve with repeated prone positions, and the panel recommends offering them ECMO earlier. Future research to answer this question is recommended.*

**End of the meeting:**

***Dr. Ahmed Rabie:***

thank you, thank you all, thank you, we will terminate this phase, summarize, and will send you all to sit for another phase; most probably, it will not be face to face unless if we have any new events, if not we will make it as a zoom conference agree? OK!

(*) proposed recommendation not discussed during the meeting.


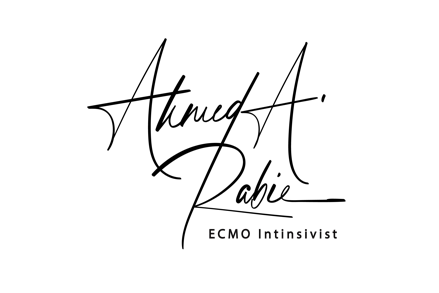


**Supplementary 5**

**Round 1, R1 voting analysis.**


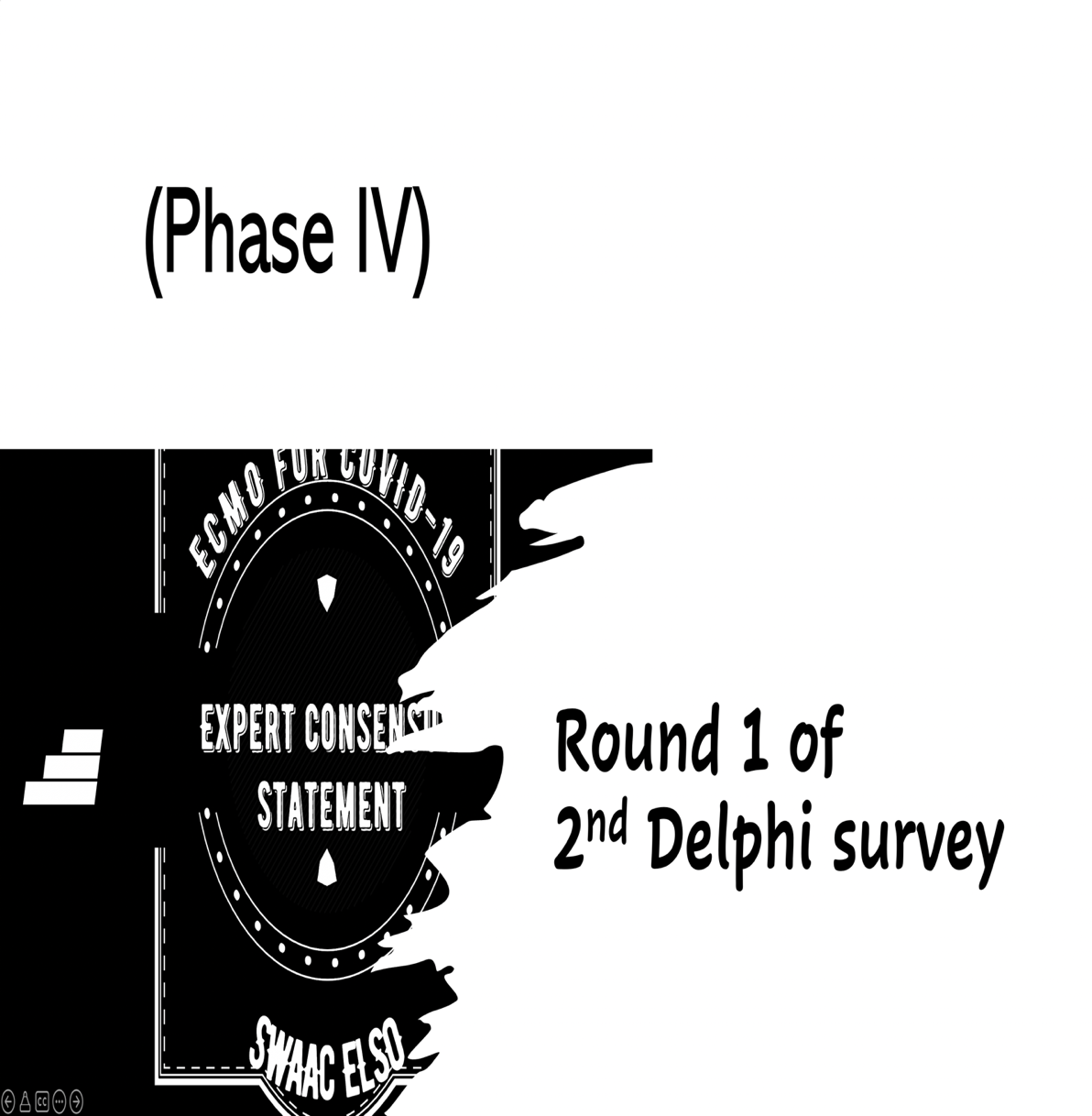


**Results of** (Phase IV. R1)

- 11 statements showed strong consensus.
- 4 statements showed weak consensus.
- weak consensus statements will be modified according to comments sent for second round.


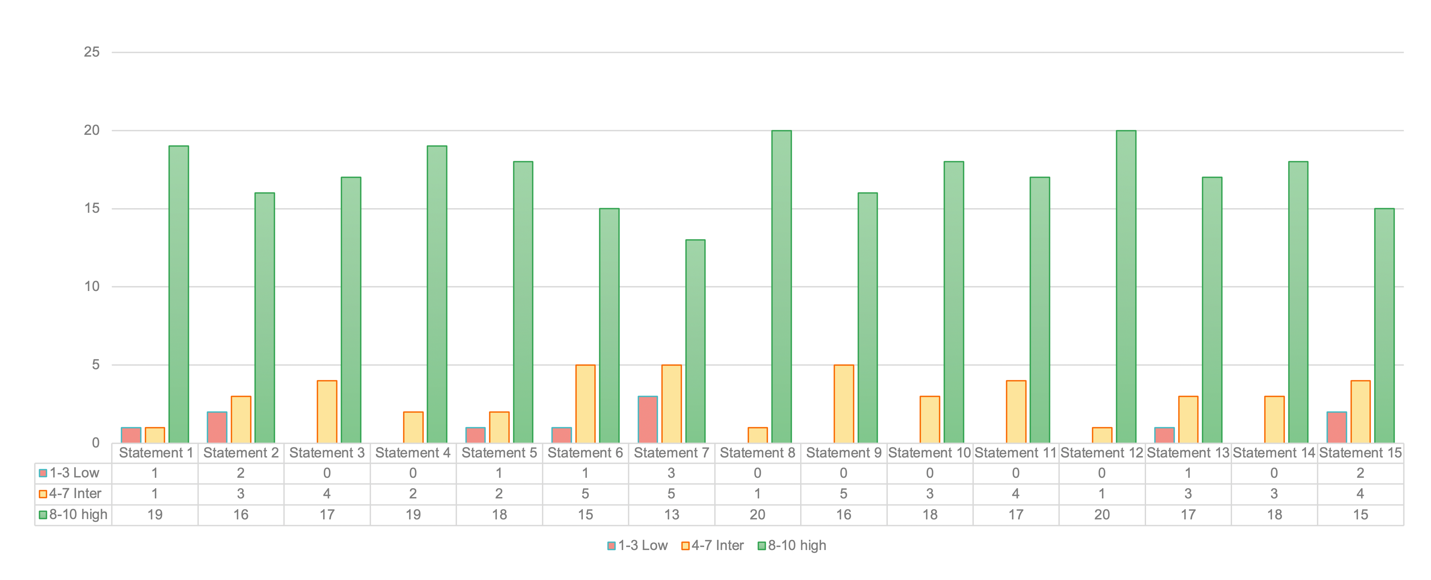


***Figure 2.*** *Number of votes according to level of agreement for the statements (21 total votes)*


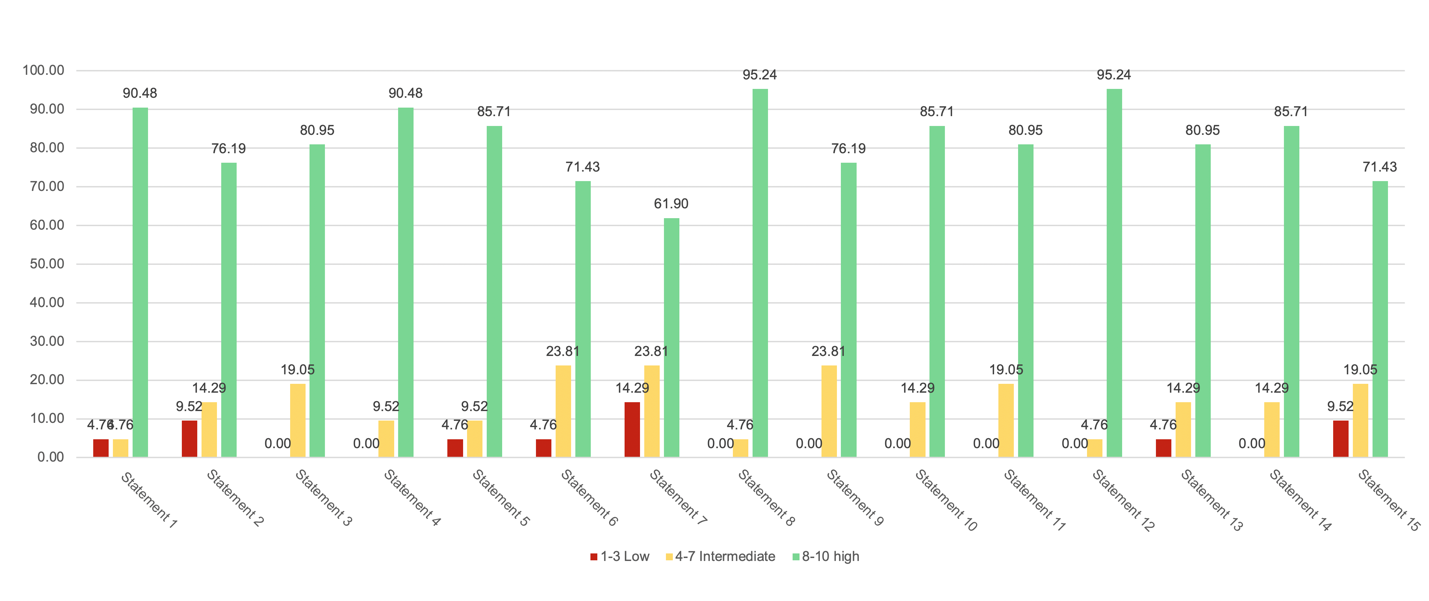
*Percentage.*

***Figure 2.*** *Number of votes according to level of agreement for the statements (21 total votes)*

*Percentage.*

**Analysis of R1 voting on expert statements of ECMO for COVID-19**


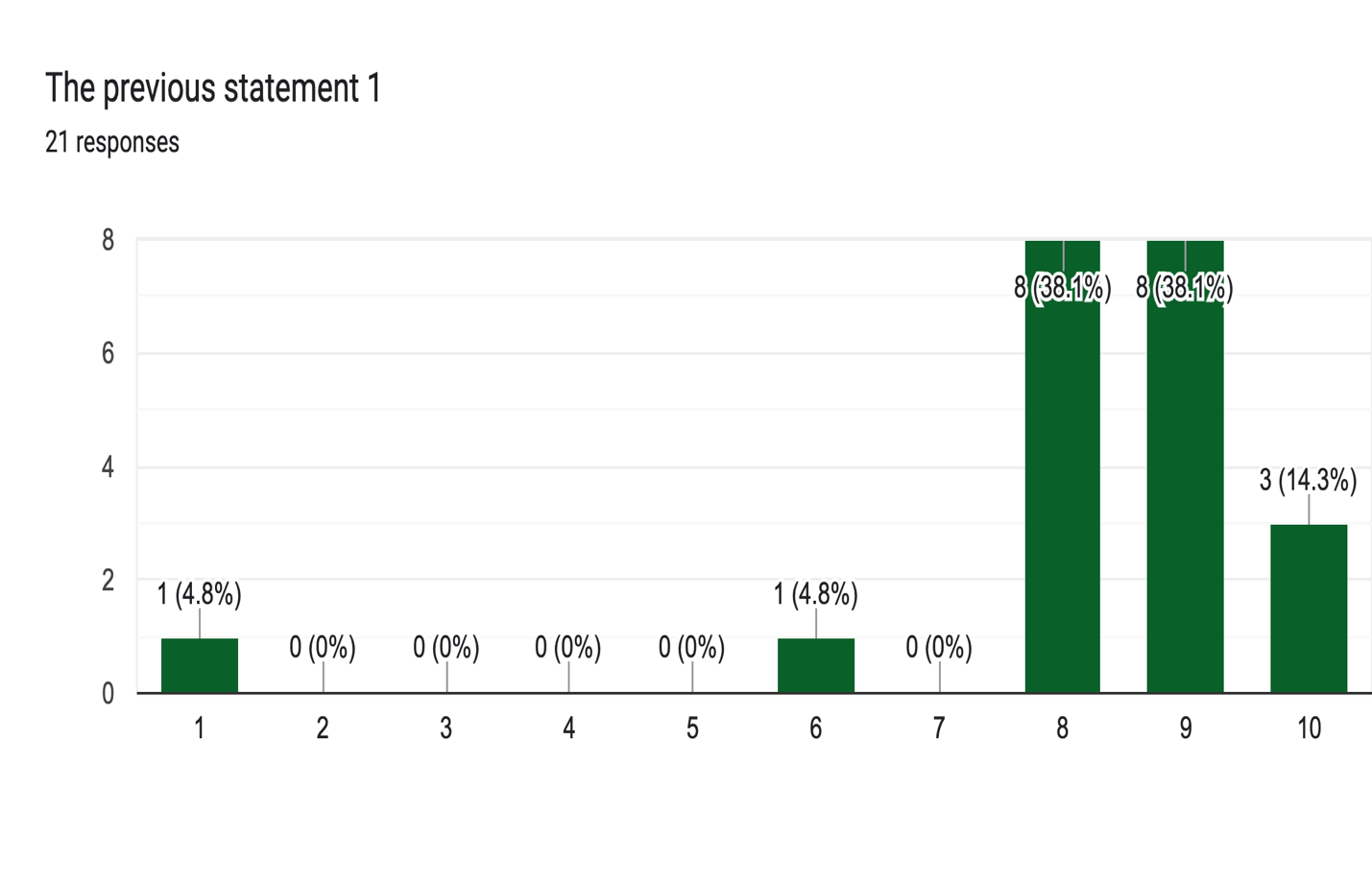


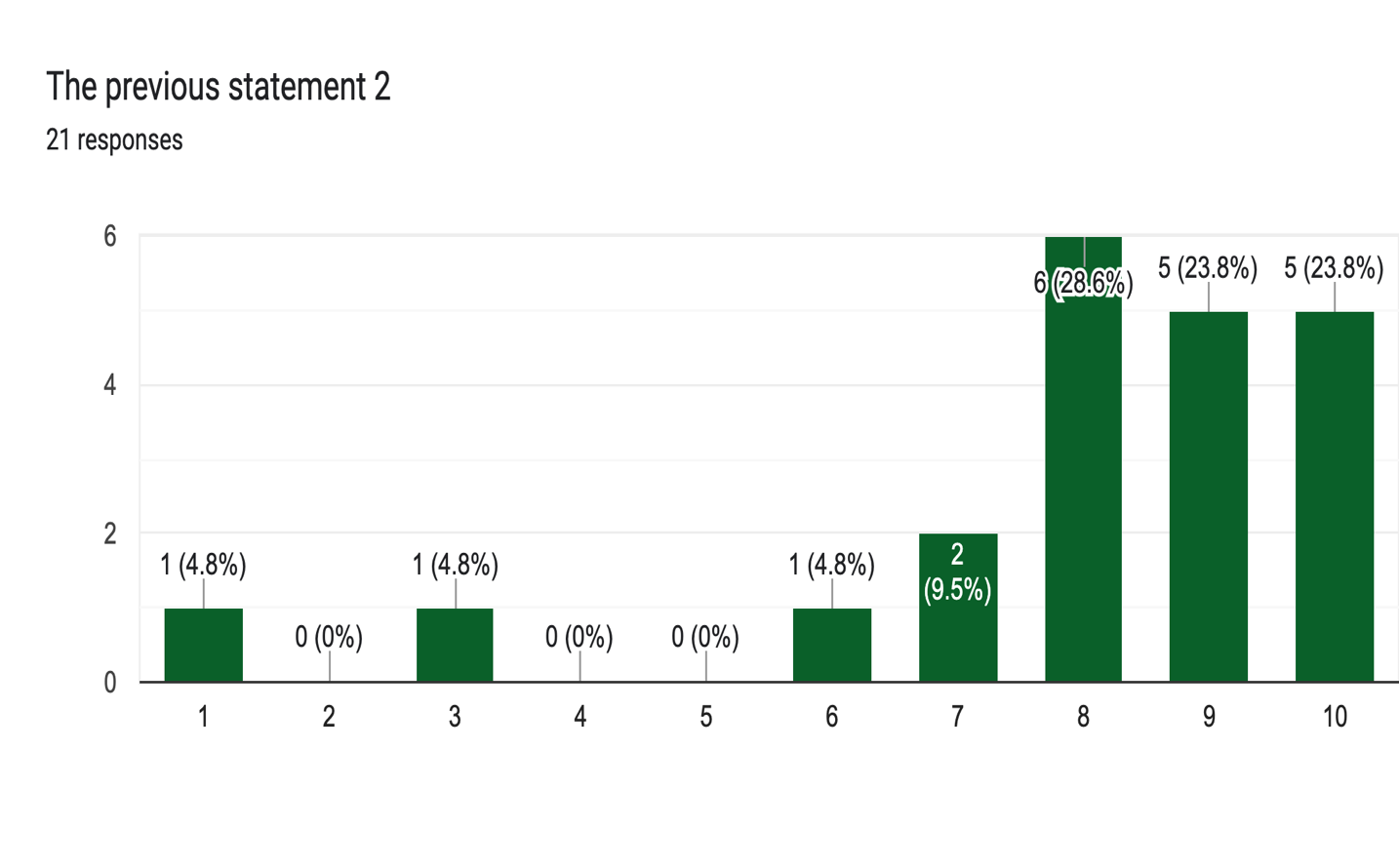


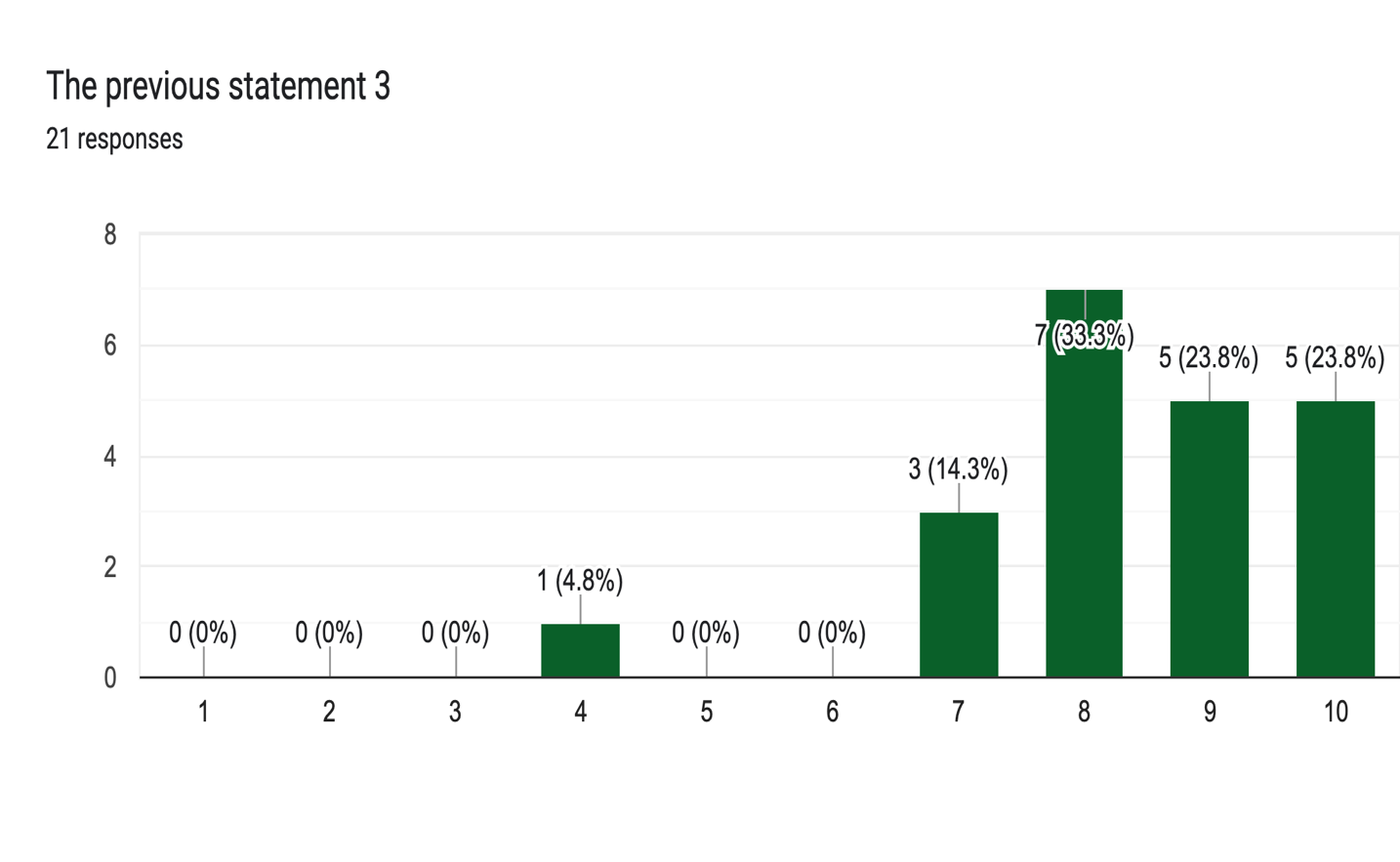


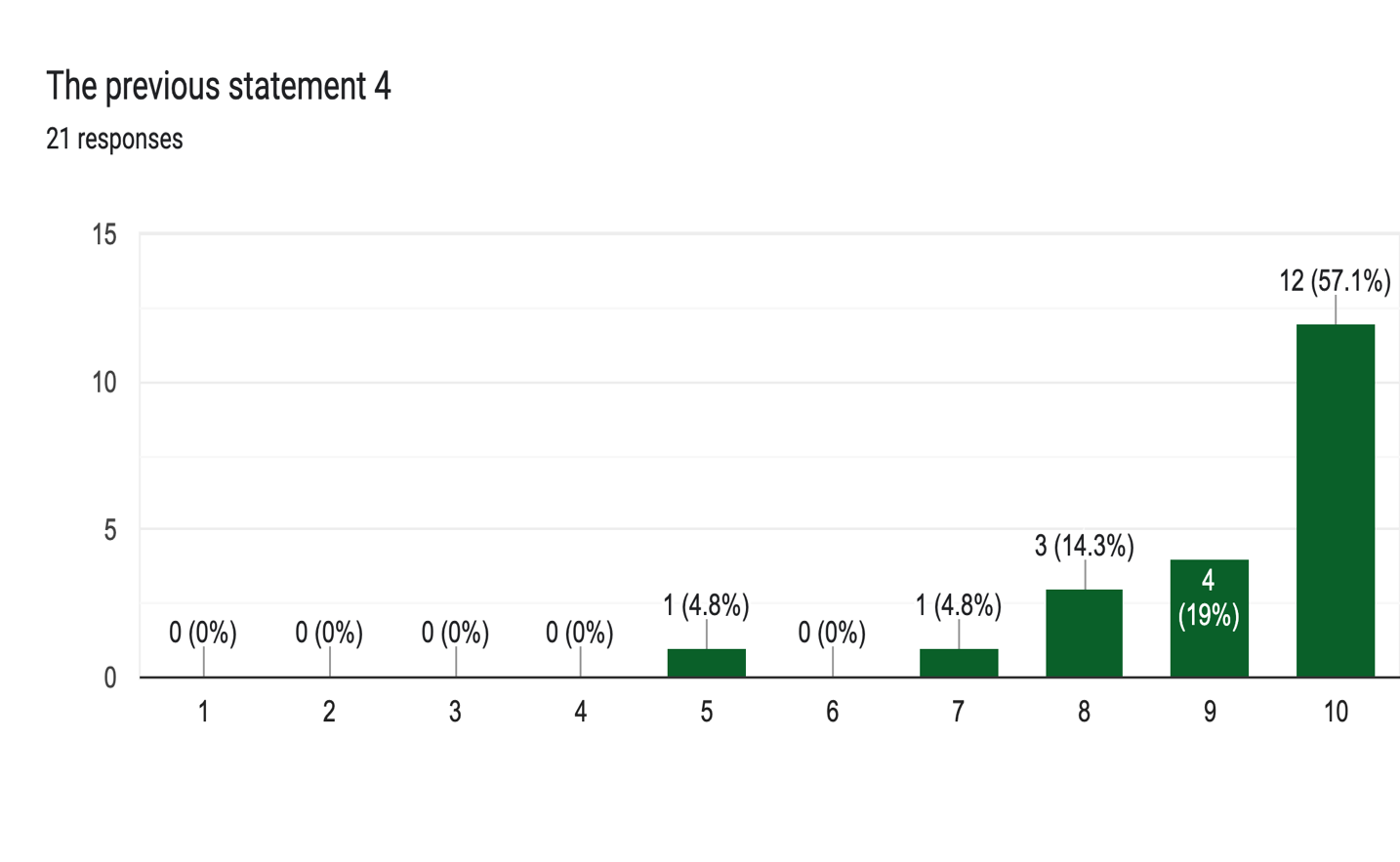


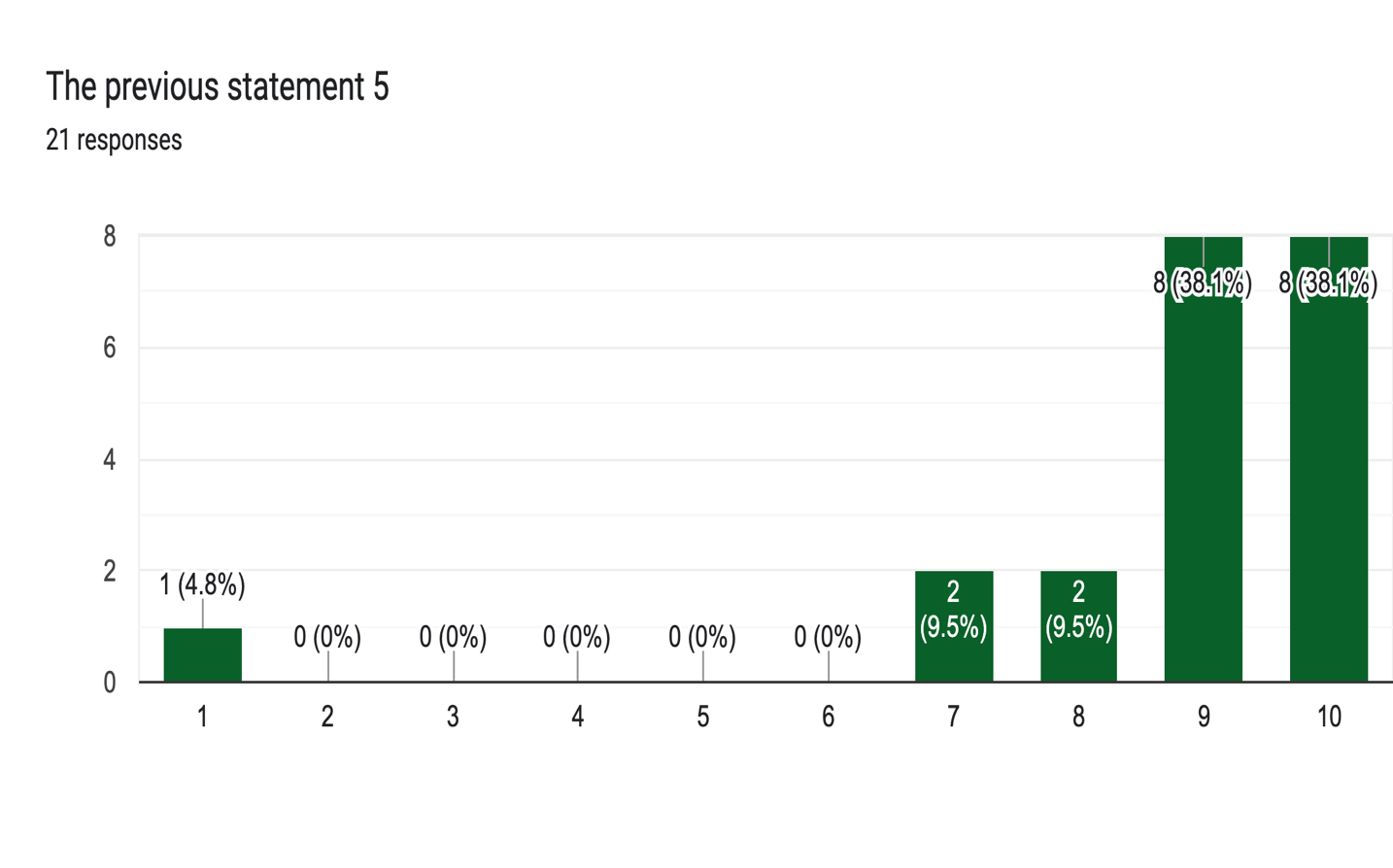


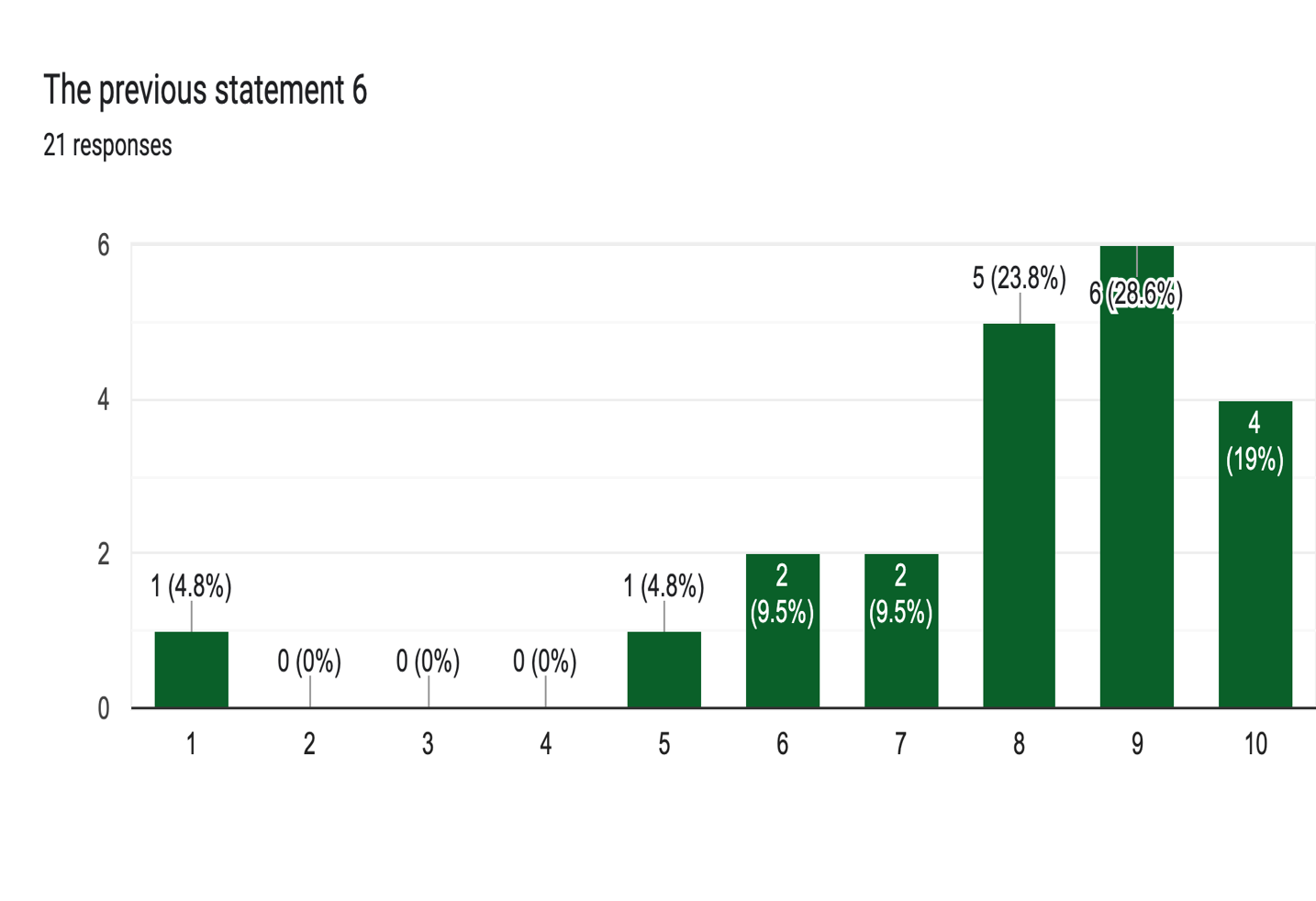


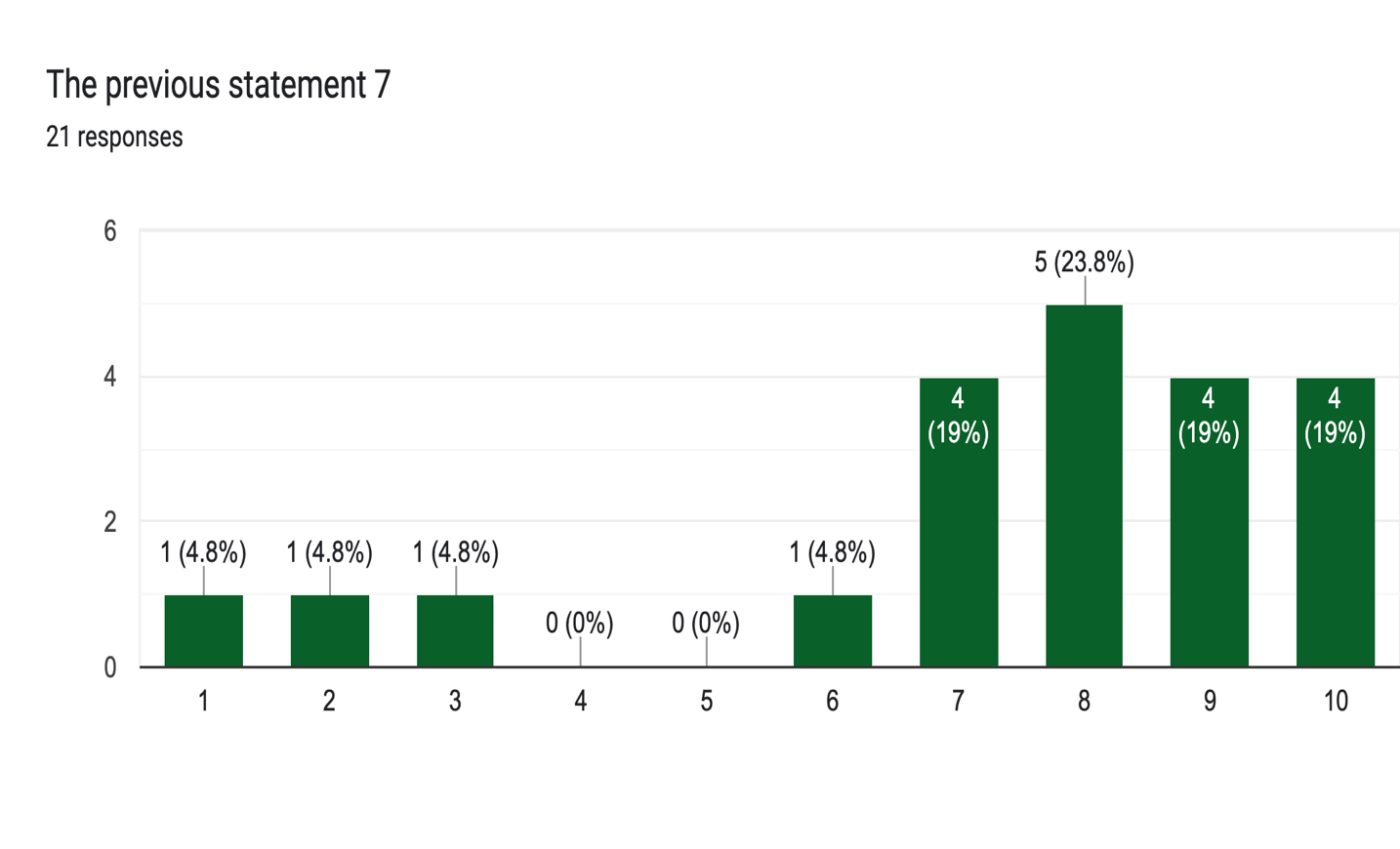


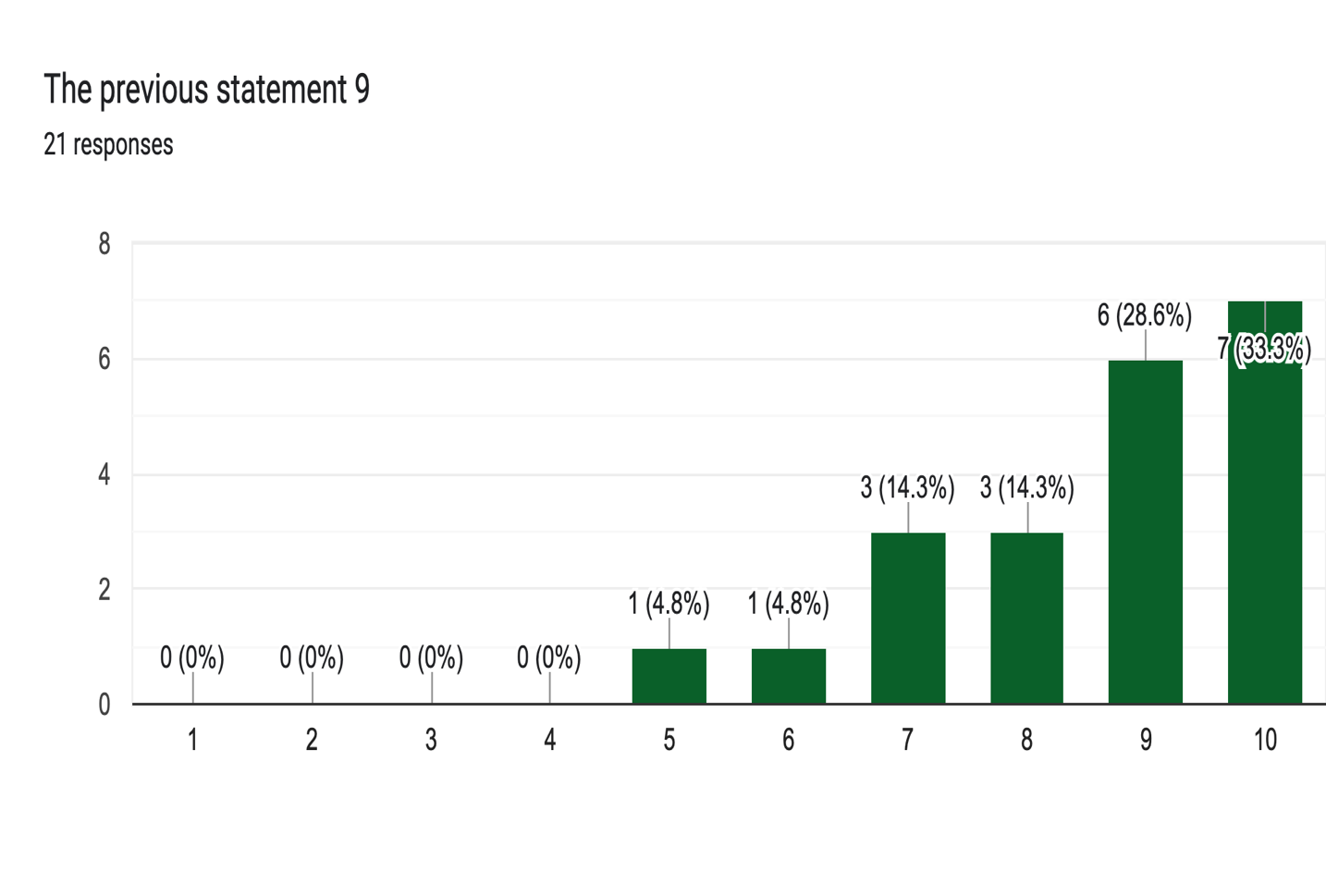


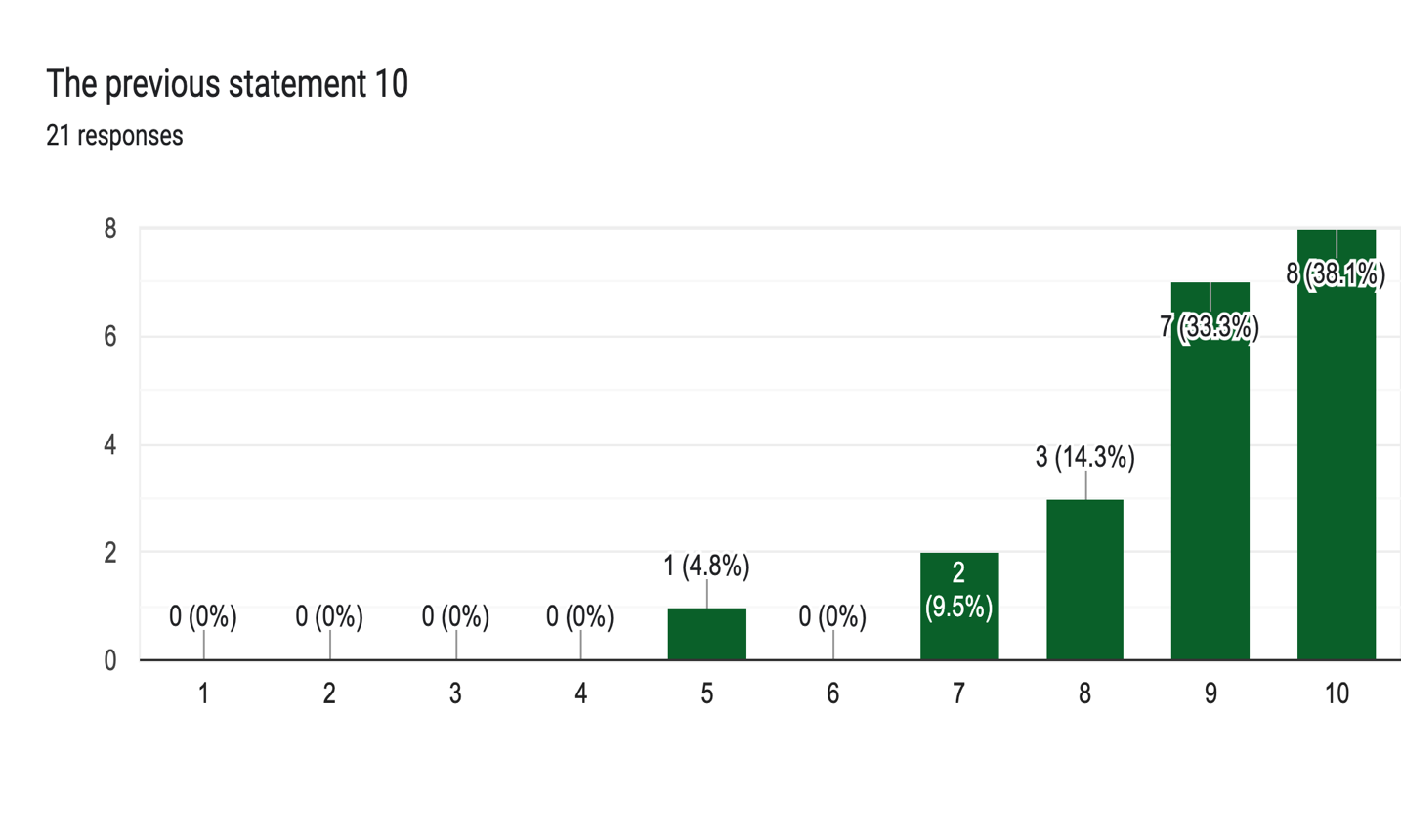


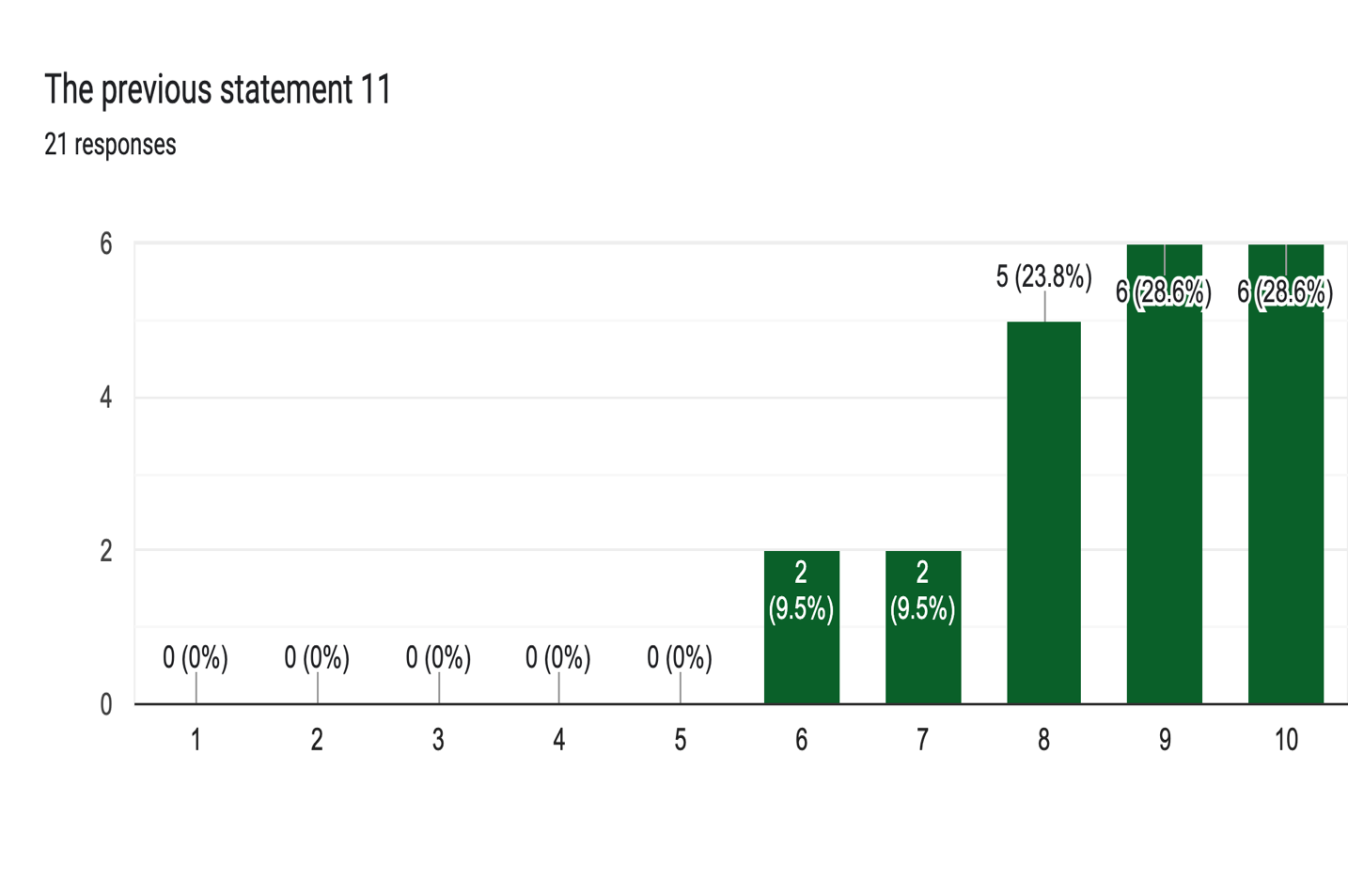


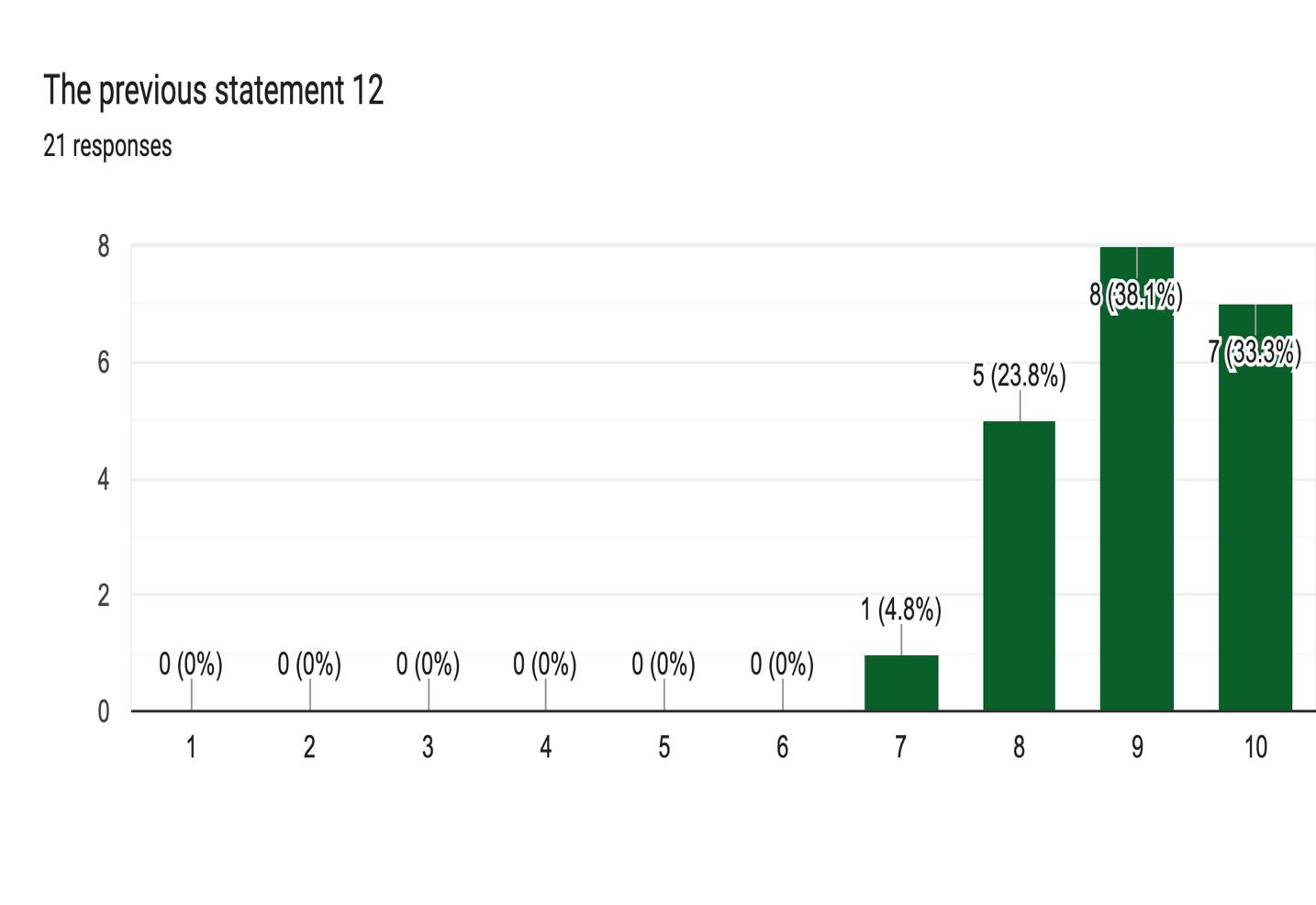


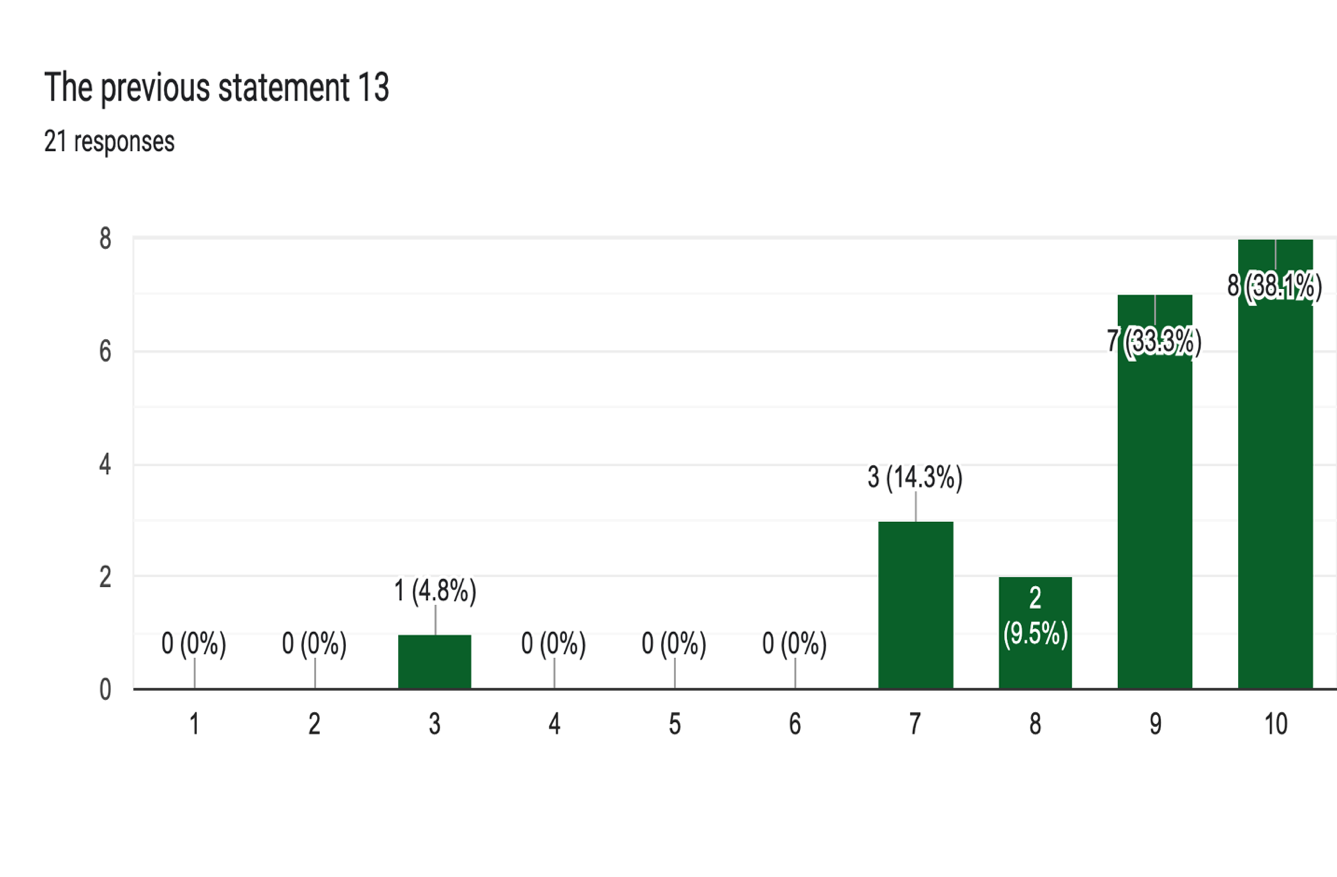


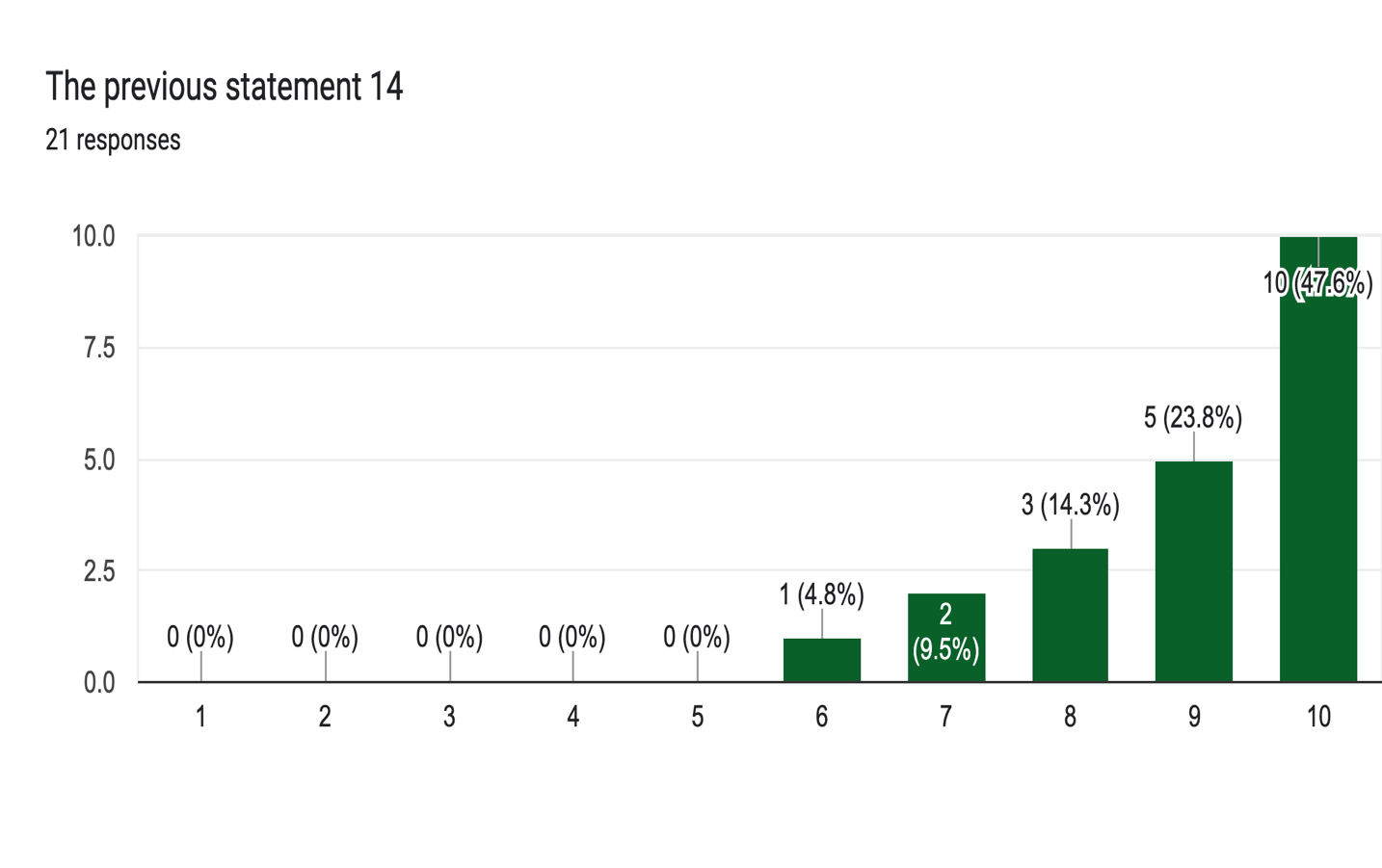


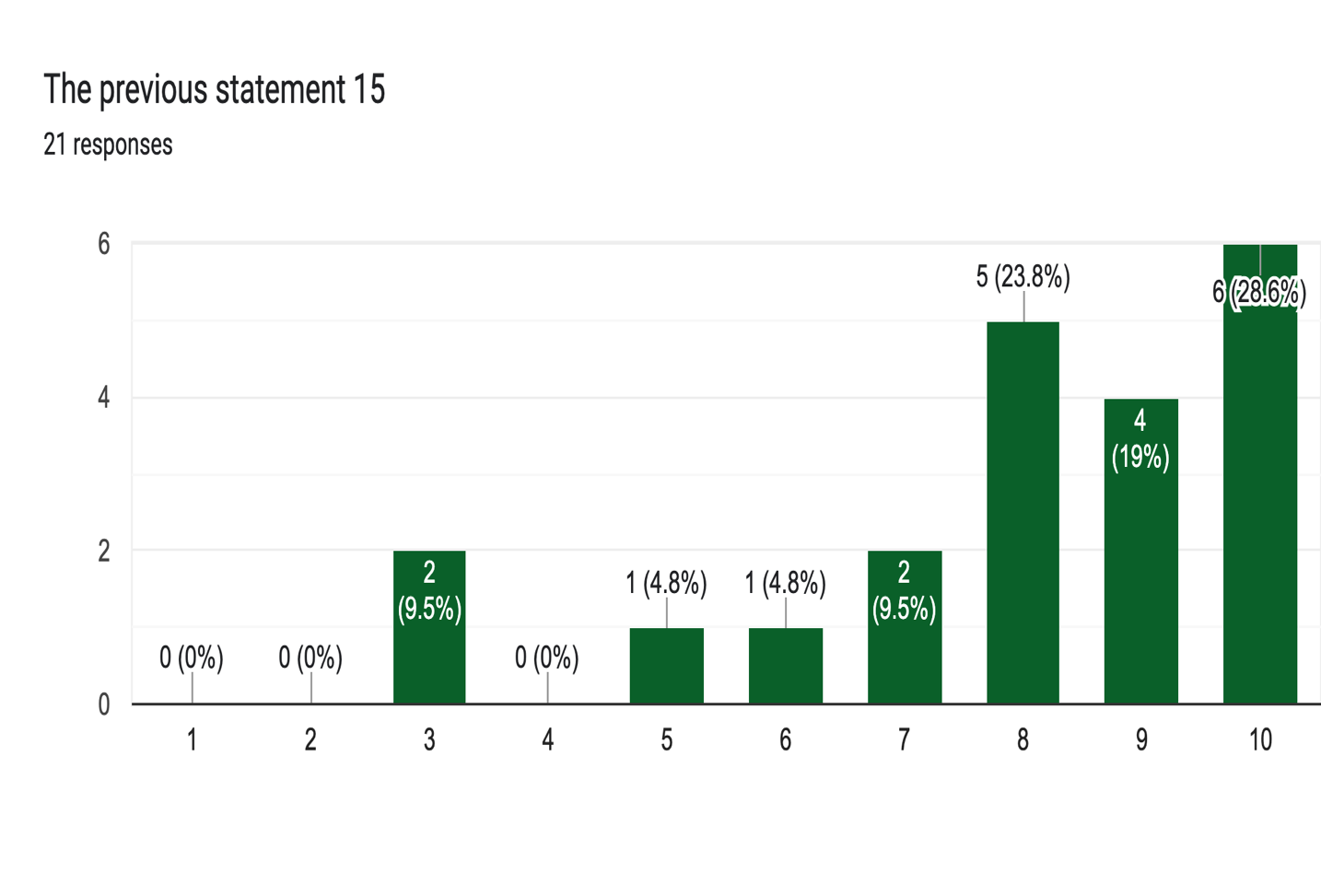


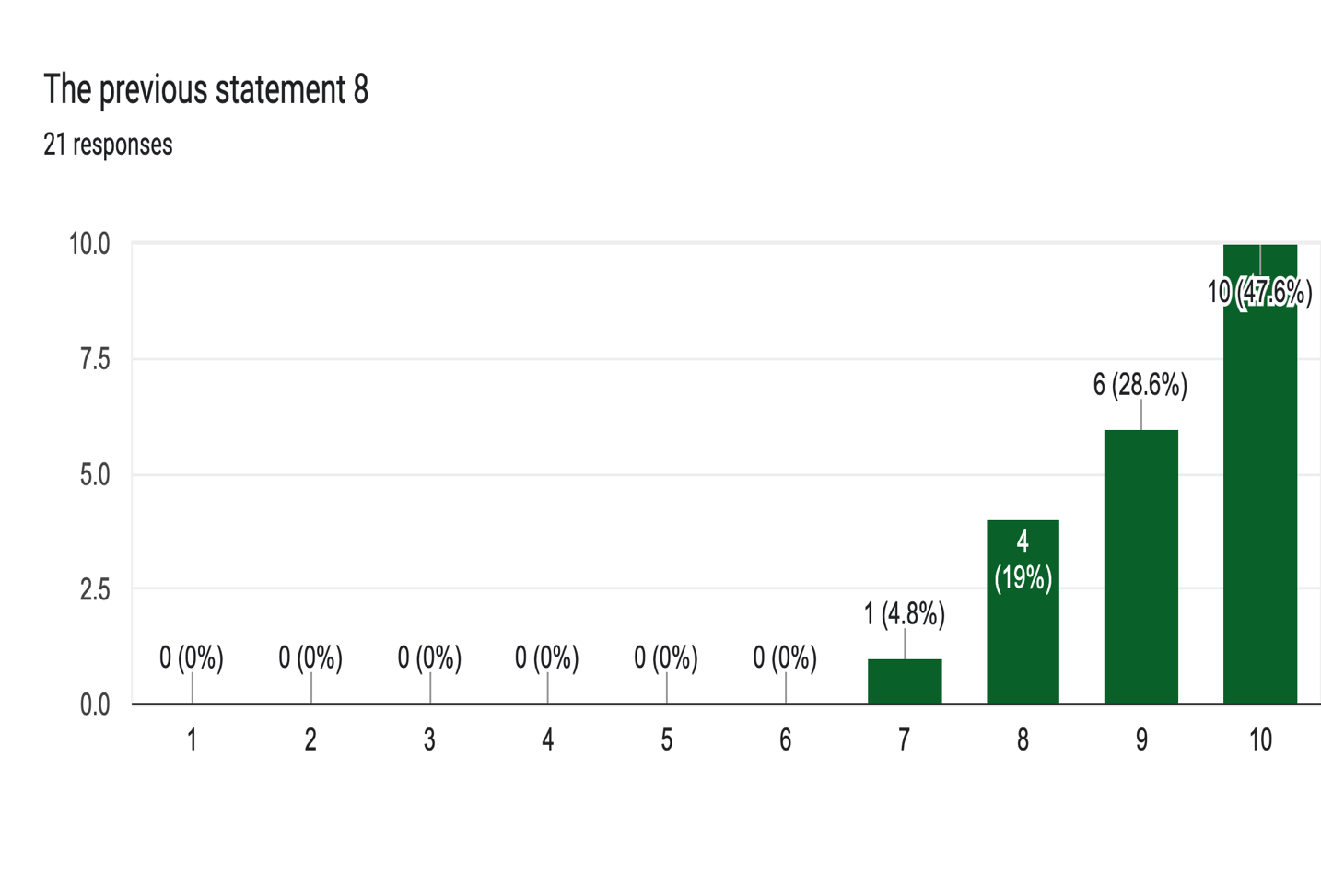


**Supplementary 6**

**Round 2, R2 voting analysis.**

**Results of R2 voting:**

- Four weak statements were modified from R1, and the faculty vote anonymously with 95% response rate 20/21.
- Phase IV completed.
- **Strong statements (>80%)**
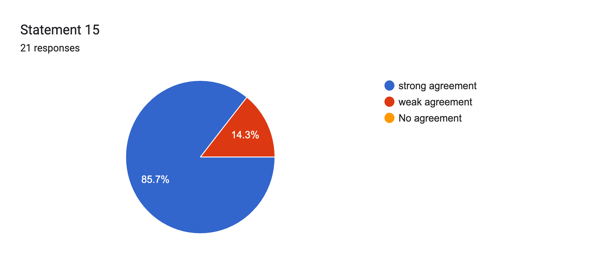

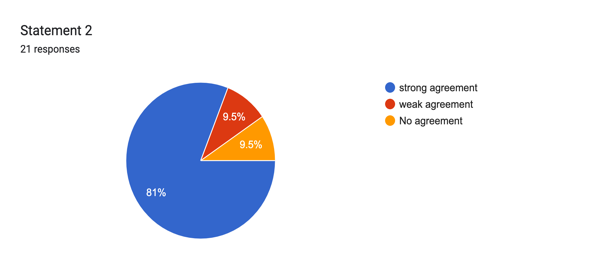

- **Weak statements (70-80%)**


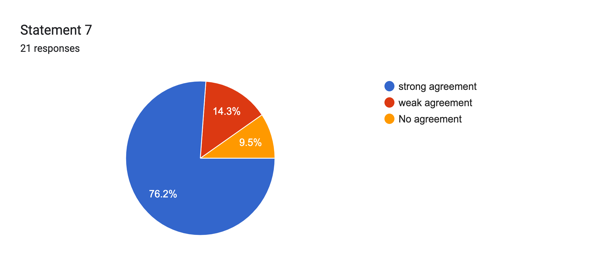

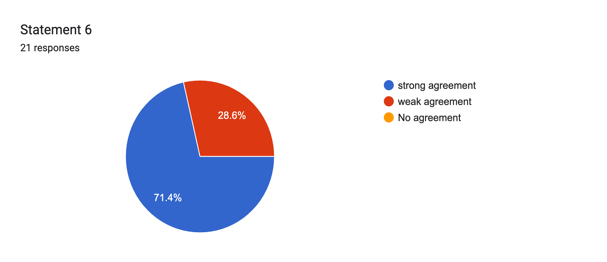


**Results of phase IV (voting phase)**

- 13 statements with strong consensus.
- 2 statements with weak consensus
-
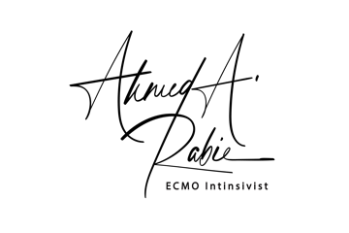
3 statements with no consensus

**Supplementary 7**

**Authors' contribution during different phases.**

|  | **Phase I** | **Phase II** | **Phase IIIa** | **Phase IIIb** | **Phase IIIc** | **Phase IV** | **Phase V** |
| --- | --- | --- | --- | --- | --- | --- | --- |
| **Number of total authors included in the phase** | 22 | 22 | 24 | 15 | 20 | 21 | 13 |
| **Main authors** | 22 | 22 | 16 | 10 | 17 | 21 | 13 |
| **Guest authors** | no | no | 8 | 5 | 3 | no | no |
| **Location** | email | Google form survey | Hybrid Riyadh | Hybrid Riyadh | Hybrid London | Web based | Zoom and email |
| **Achievements** | Expert composition and 52 questions were gathered that are controversy | 39 questions evaluated for validation validated (table 1) | 15 statements were formulated.  3 validated questions or topics could not be formulated into a statement | | | 13 strong statements  2 weak statements | pre-publication manuscript |
| **Authors' contributions (guest authors highlighted with yellow)** | AR, AE, AA, MHA, and AL. | AR, AE, AA, MHA, AL, DB, AC, BZ, GP, GM, RPB,MVA, KS, AAF, PO, YM, HA, KR, MO, LR, MP and RB. | AR, AE, AA, MA, AL, AC, GM,MVA, AAF, PO, YM, HA, MO, LR, MP.AAF, MA, HB, MR, YA, AAB,GVL, MT | AR, AE, MHA, AL, BZ, GP,MVA, AAF, PO, YM, HA,  AYA,MR, RA, MF, MT | AR, AE, AA,MHA, AL, BZ, GP, RPB, MVA, KS, AAF, PO, HA, KR, MO, LR, and RB.  MT,GVL, AAB | AR, AE, AA,MHA, AL, DB, AC, BZ, GP, GM, RPB, MVA, KS, AAF, PO, YM, HA, KR, LR, MP and RB. | AR, AE, AA,MHA, AL, DB, AC, BZ, GP, GM, RPB, MVA and KS. |

**Supplementary 8**

**Flowcharts include the entire process of expert consensus statement progress.**


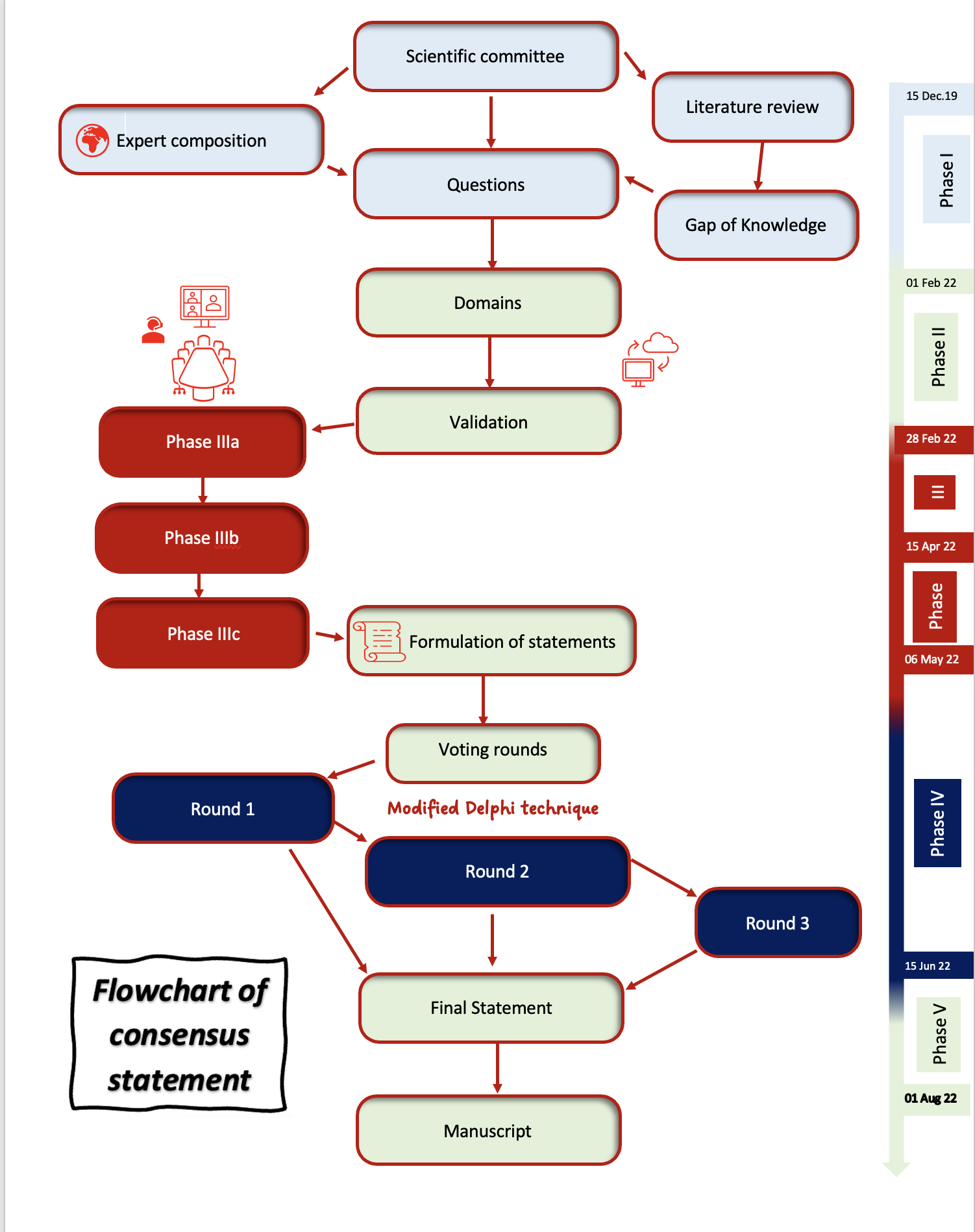


**Supplementary 9**

**The expert consensus statement logo.**


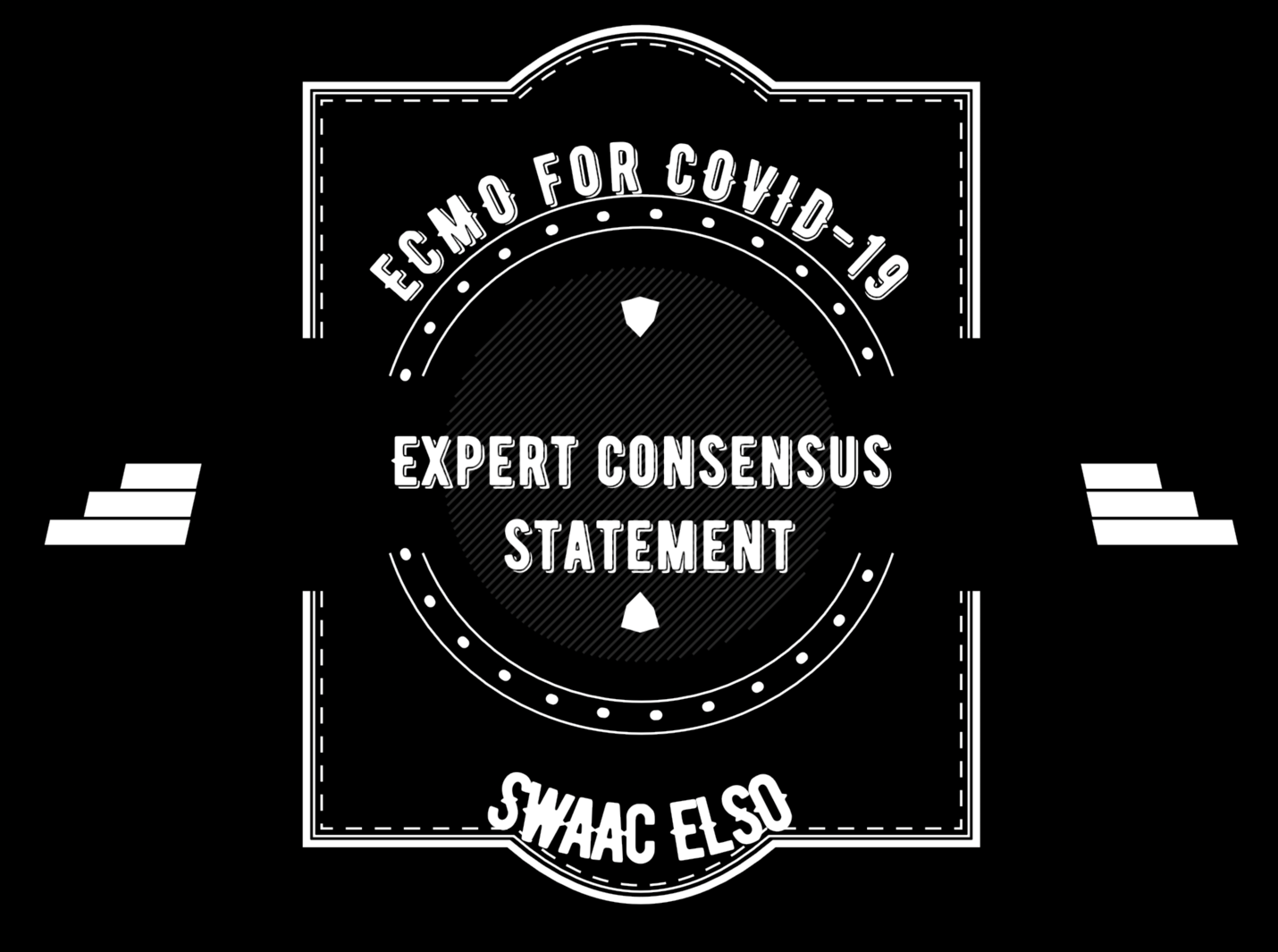

Supplement: Supplementary file 1 — Additional file 1: Expert consensus statements’ meeting minutes, validation of the questions, statement formulation and analysis of the faculty's responses. [file 13613_2023_1126_MOESM1_ESM.docx]
